# Insights into the Mechanistic Basis of Plasmid-Mediated Colistin Resistance from Crystal Structures of the Catalytic Domain of MCR-1

**DOI:** 10.1038/srep39392

**Published:** 2017-01-06

**Authors:** Philip Hinchliffe, Qiu E. Yang, Edward Portal, Tom Young, Hui Li, Catherine L. Tooke, Maria J. Carvalho, Neil G. Paterson, Jürgen Brem, Pannika R. Niumsup, Uttapoln Tansawai, Lei Lei, Mei Li, Zhangqi Shen, Yang Wang, Christopher J. Schofield, Adrian J Mulholland, Jianzhong Shen, Natalie Fey, Timothy R. Walsh, James Spencer

**Affiliations:** 1 School of Cellular and Molecular Medicine, University of Bristol, Bristol BS8 1TD, UK; 2 Institute of Infection and Immunity, Cardiff University, Cardiff CF14 4XN, UK; 3 School of Chemistry, University of Bristol, Bristol BS8 1TS, UK; 4 Beijing Advanced Innovation Center for Food Nutrition and Human Health, College of Veterinary Medicine, China Agricultural University, Beijing, China; 5 Diamond Light Source, Harwell Science and Innovation Campus, Didcot OX11 0DE, UK; 6 Department of Chemistry, University of Oxford, Oxford OX1 3TA, UK; 7 Department of Microbiology and Parasitology, Faculty of Medical Science, Naresuan University, Phitsanulok, 65000, Thailand

## Abstract

The polymixin colistin is a “last line” antibiotic against extensively-resistant Gram-negative bacteria. Recently, the *mcr-1* gene was identified as a plasmid-mediated resistance mechanism in human and animal Enterobacteriaceae, with a wide geographical distribution and many producer strains resistant to multiple other antibiotics. *mcr-1* encodes a membrane-bound enzyme catalysing phosphoethanolamine transfer onto bacterial lipid A. Here we present crystal structures revealing the MCR-1 periplasmic, catalytic domain to be a zinc metalloprotein with an alkaline phosphatase/sulphatase fold containing three disulphide bonds. One structure captures a phosphorylated form representing the first intermediate in the transfer reaction. Mutation of residues implicated in zinc or phosphoethanolamine binding, or catalytic activity, restores colistin susceptibility of recombinant *E. coli*. Zinc deprivation reduces colistin MICs in MCR-1-producing laboratory, environmental, animal and human *E. coli*. Conversely, over-expression of the disulphide isomerase DsbA increases the colistin MIC of laboratory *E. coli*. Preliminary density functional theory calculations on cluster models suggest a single zinc ion may be sufficient to support phosphoethanolamine transfer. These data demonstrate the importance of zinc and disulphide bonds to MCR-1 activity, suggest that assays under zinc-limiting conditions represent a route to phenotypic identification of MCR-1 producing *E. coli*, and identify key features of the likely catalytic mechanism.

Antibiotic resistance, particularly in Gram-negative bacteria (GNB) is a serious and growing global threat. GNB constitute a growing proportion of nosocomial infections, contributing to increased morbidity and mortality in vulnerable patient groups such as surgery, transplant and chemotherapy patients^1^. Enterobacteriaceae like *Escherichia coli* and *Klebsiella pneumoniae* are versatile pathogens causing sepsis, wound, surgical site, respiratory, urinary tract and device-associated infections^2^,^3^. Treatment of GNB infections is complicated by their intrinsic resistance to many antibiotic classes and ready acquisition of resistance to additional agents^4^. Widespread dissemination of plasmids containing multiple resistance determinants has eroded treatment options leaving few reliable antibiotics for empiric therapy, a situation exacerbated by the continuing shortage of new antibacterials effective against GNB^5^.

The polymixin colistin is a key therapeutic for GNB infections as the spread of mobile antibiotic resistance increases treatment failure for third generation cephalosporins or carbapenems^6^. Until recently colistin resistance in Enterobacteriaceae was considered uncommon, arising largely from chromosomal mutations in *K. pneumoniae* strains^7^. However, recently a plasmid-encoded colistin resistance determinant, MCR-1, was identified in an animal-associated *E. coli* strain, and subsequently found on multi-resistance plasmids from animal, retail meat and human *E. coli* and *K. pneumoniae*^8^. MCR-1 dissemination, which is already global^9^, now threatens the continuing effectiveness of colistin against GNB infections. In particular, GNB carrying plasmids co-expressing MCR-1 and carbapenemases alongside other resistance determinants may be effectively untreatable with conventional antibiotics^10^,^11^. MCR-1 confers resistance by modifying the colistin target, catalyzing transfer of phosphoethanolamine (PEA) onto the glucosamine saccharide of lipid A in the bacterial outer membrane (Fig. 1)^8^. This reduces the net negative charge of the lipid A head group and, consequently, colistin binding^12^. The catalytic mechanism of MCR-1 (and other bacterial PEA-transferases) remains to be established. Here we present two crystal structures of the soluble, catalytic domain of MCR-1, revealing this to be a zinc metalloprotein containing three intramolecular disulphide bonds. Colistin susceptibility assays support the proposed importance of both zinc and disulphide bond formation to MCR-1 activity, while density functional theory models of key states on the proposed reaction pathway indicate that both mono- and di-zinc forms of the enzyme may be able to support PEA transfer.

## Results

### Crystal Structure of MCR-1 Catalytic Domain

**Figure 2:**
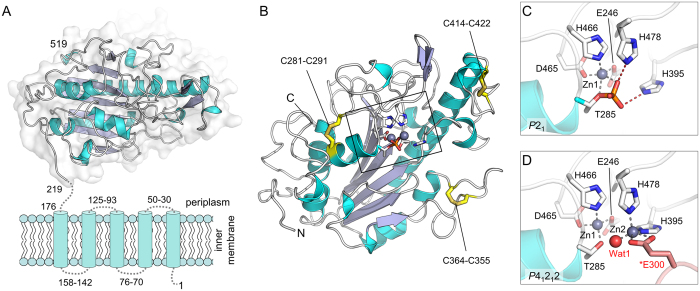
Structure of MCR-1 Periplasmic Domain. (**A**) Organisation of MCR-1 showing 5 predicted membrane-spanning α-helices and the soluble periplasmic domain (residues 219–541) crystallised here. (**B**) Overall fold of MCR-1 catalytic domain. Intramolecular disulphide bonds are labeled and metal centre (active site) is boxed. (**C**) Active site of *P*2_1_ crystal form showing single bound zinc ion (grey sphere) and phosphorylation of Thr285. (**D**) Active site of *P*4_1_2_1_2 crystal form showing dinuclear zinc centre and coordination of Zn2 by residue Glu300 (pink) from an adjacent molecule in the crystal lattice.

Bacterial lipooligosaccharide PEA-transferases such as MCR-1 are organised into two domains: an N-terminal inner membrane-bound domain predicted to contain 5 transmembrane α-helices and a soluble, periplasmic domain containing the putative catalytic centre^8^,^13^,^14^,^15^. Hence, as a first step towards understanding the molecular mechanism of MCR-1 mediated colistin resistance, we sought to obtain high-resolution structural information for the MCR-1 catalytic domain. To facilitate such studies, we engineered MCR-1 to lack the five transmembrane helices, generating a construct containing residues 219–541 (MCR1ΔTM) which was purified and crystallised. Phases calculated from datasets collected at or near the zinc X-ray absorption edge ultimately yielded structures for two different crystal forms in the space groups *P*4_1_2_1_2 (1.75 Å resolution) and *P*2_1_ (1.55 Å resolution) (Supplementary Table S1). The two structures are essentially identical (RMSD 0.37 Å over 311 Cα atoms, Supplementary Figure S1) and reveal an overall α-β-α fold, containing three intramolecular disulphide bonds, typical of alkaline phosphatase or sulphatase enzymes, with the N-terminus indicating the likely position of the five transmembrane helices (Fig. 2A,B). Both space groups contain an identical dimeric form, through interactions between monomers in the asymmetric unit (*P*2_1_) or symmetry-related molecules (*P*4_1_2_1_2) (Supplementary Figure S2). However, size exclusion chromatography experiments show MCR1ΔTM is monomeric in solution (Supplementary Figure S2), leading us to conclude that this dimeric form is unlikely to be physiologically relevant.

Both structures contain clear electron density for bound metal ion(s) in the putative active site, assigned as zinc on the basis of the presence of excess zinc in the purification buffers and a strong signal at the zinc absorption edge in X-ray fluorescence scans of MCR-1 crystals (Supplementary Figure S3). The consistency with DFT-optimised structures (see below) also supports their identification as zinc ions. However, the two crystal forms differ in zinc content, with the *P*2_1_ structure containing one zinc ion (Zn1), coordinated by Asp465, His466, Glu246 and Thr285 in a tetrahedral geometry (Fig. 2C, Supplementary Table S2), while the *P*4_1_2_1_2 structure contains density for an additional, also tetrahedral, zinc site (Zn2) coordinated by His395, His478, a water molecule (Wat1) and Glu300 from a symmetry related molecule (Fig. 2D, Supplementary Table S2). Furthermore, the active site of the *P*2_1_ structure contains electron density corresponding to the presence of a phosphate group attached to Thr285, the conserved residue believed to act as the acceptor for the PEA group during the transfer reaction^13^,^15^. This apparent autophosphorylation was confirmed by mass spectrometry of MCR1ΔTM crystallisation samples, which showed a mixed population of proteins with a mass difference (78 Da) consistent with the presence of phosphorylated and non-phosphorylated forms (Supplementary Figure S4).

The MCR-1 active site occupies a shallow depression on a relatively flat face of the molecule, potentially permitting binding of the relatively large lipid A headgroup in more than one orientation and enabling modification at more than one position^16^. Plots of surface hydrophobicity and charge distributions (Supplementary Figure S5) show this region to be both more hydrophobic and less charged than the rest of the protein, consistent with a location adjacent to the membrane surface from which the substrates are expected to protrude. In addition to Thr285 and the residues that make up the Zn1 site, the active site region contains a number of residues conserved between bacterial PEA-transferases^8^. In particular, in addition to their possible roles as Zn2 ligands (*P*4_1_2_1_2 structure), in the *P*2_1_ structure both His395 and His478 are well positioned to interact with phosphorylated Thr285, whilst the proximity of Lys333 to Thr285 suggests a possible role for this residue in substrate (PEA) binding. We also note the presence of a strong hydrogen bond between ND1 of His478 and OE1 of the conserved Glu468, an interaction that could plausibly orient or polarise this histidine as part of a mechanistic role.

### Requirements for MCR-1 Activity

**Figure 3:**
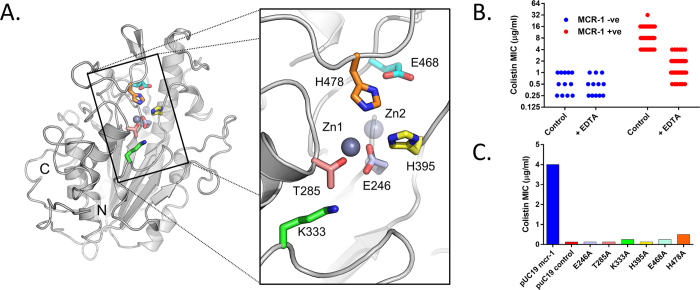
Effect of Mutation and Zinc Deprivation upon MCR-1 Activity. (**A**) MCR-1 active site (*P*2_1_ crystal form) with colours identifying positions of alanine substitutions. (**B**) Effect of zinc deprivation on colistin Minimal Inhibitory Concentrations (MICs) for MCR-1-negative (12 isolates; blue) and MCR-1-expressing (68 isolates; red) *E. coli*. MICs were determined by broth microdilution in the presence and absence of EDTA (250 μg/ml) as described. (**C**) Colistin MICs (determined by agar dilution, data shown are modes for two independent experiments, each performed in duplicate) for alanine substitutions at positions shown in (**A**).

The crystallographic results indicate that MCR-1 is a zinc metalloprotein containing at least one zinc ion, with both a conserved Thr residue (Thr285) able to autophosphorylate in recombinant *E. coli* and a network of intramolecular disulphide bonds. We therefore sought to test the hypotheses that zinc is important to the activity of MCR-1 in the bacterial host, that conserved amino acids participate in zinc/substrate binding or in the phospho(ethanolamine) transfer reaction, and that disulphide bond formation in the periplasm is important to MCR-1 activity. We evaluated the effects of zinc deprivation, modification of specific amino acids (Fig. 3A), or enhanced disulphide bond formation, upon MCR-1 activity *in situ* as measured by colistin minimal inhibitory concentrations (MICs) for *mcr-1*-positive bacteria. Removal of zinc through inclusion of the chelator EDTA in MIC experiments reduced the colistin MICs of laboratory *E. coli* expressing full-length recombinant MCR-1 from 2 μg/ml to that of vector-only controls (0.25 μg/ml). Profound reductions in colistin MIC (up to 5 dilutions) on EDTA exposure were also observed when these experiments were extended to a panel of 68 *E. coli* strains of environmental, animal and human origins (Fig. 3B, Supplementary Figure S6, Supplementary Table S3) supporting a requirement for zinc (or possibly other divalent cations) in MCR-1 function. Importantly, EDTA treatment had little effect upon the growth or colistin susceptibility of a panel (12 strains including one type strain) of *mcr-1* negative *E. coli* collected during the same sampling operations. In the absence of EDTA, these negative control samples varied in their colistin susceptiblity (MICs ≤ 0.25 to 1 μg/ml) up to levels at which significant reductions in MIC are easily measurable. However, for these strains increases in colistin susceptibility on EDTA treatment were at most one dilution, indicating that EDTA is not affecting membrane permeability to colistin, and that MIC reductions in MCR-1-positive strains are rather due to a loss of MCR-1 activity.

These results imply that divalent cations, specifically zinc, are important to MCR-1 activity. This inference is further supported by the observation that replacement of the zinc ligand Glu246 by alanine reduces the colistin MIC of recombinant *E. coli* to that of vector-only control (Fig. 3C, Supplementary Table S4), an effect equivalent to substitution of the acceptor threonine (Thr285). The effects of mutations at other active site residues are however more variable. Whilst replacement of the conserved His395, part of the Zn2 site (*P*4_1_2_1_2 structure), reduces colistin MIC to basal levels, substitutions of the adjacent His478, or its hydrogen-bonding partner Glu468, give smaller reductions (3 to 4 dilutions). Similarly, the alanine mutation of Lys333 substantially reduces colistin MIC, but not to basal levels. Thus, at positions other than His395, alanine mutants away from the Zn1 site retain some ability to protect expressing strains from colistin action. Hence, while important, these residues are not absolutely essential to MCR-1 function. Conversely, enhanced disulphide bond formation through overexpression of the periplasmic dithiol-disulphide oxidoreductase DsbA has been shown to increase expression and activity of *N. meningitidis* LptA in recombinant *E. coli*^17^. We therefore tested whether DsbA co-expression could also increase MCR-1 activity, and established that this led to a modest but reproducible increase in the colistin MIC for MCR-1-expressing *E. coli* TOP10 (from 4 to 8 μg/ml). Taken together, these data indicate that zinc, conserved active site residues and disulphide bond formation are all important to the structure and activity of MCR-1.

### Density Functional Theory Models of MCR-1-catalysed PEA Transfer

Mechanistic proposals for phosphoryl transfer by e.g. alkaline phosphatase typically involve two^18^ or three^19^ metal ions. Whilst our structures unambiguously identify a zinc site (Zn1) in MCR-1 adjacent to the essential Thr285, the Zn2 site in the *P*4_1_2_1_2 crystal form involves crystallographic contacts and may not be relevant in solution. This possibility is further supported by another recently released crystal structure of the MCR-1 catalytic domain with phospho-Thr285 (PDB 5K4P^20^) that was crystallised from conditions of high zinc concentration contains zinc ions in the Zn1 site and at a number of additional positions, of which none is directly equivalent to the Zn2 site identified here. Accordingly, to examine the likely zinc stoichiometry required for PEA transfer, and as a preliminary study of MCR-1 mechanism, we used computational density functional theory (DFT)^21^,^22^ to investigate the mono- and di-zinc forms of MCR-1, corresponding to the *P*2_1_ and *P*4_1_2_1_2 crystal structures, respectively, with the aim of testing the extent to which each can support the phosphoryl transfer reaction (i.e. addition of PEA to Thr285).

**Figure 4:**
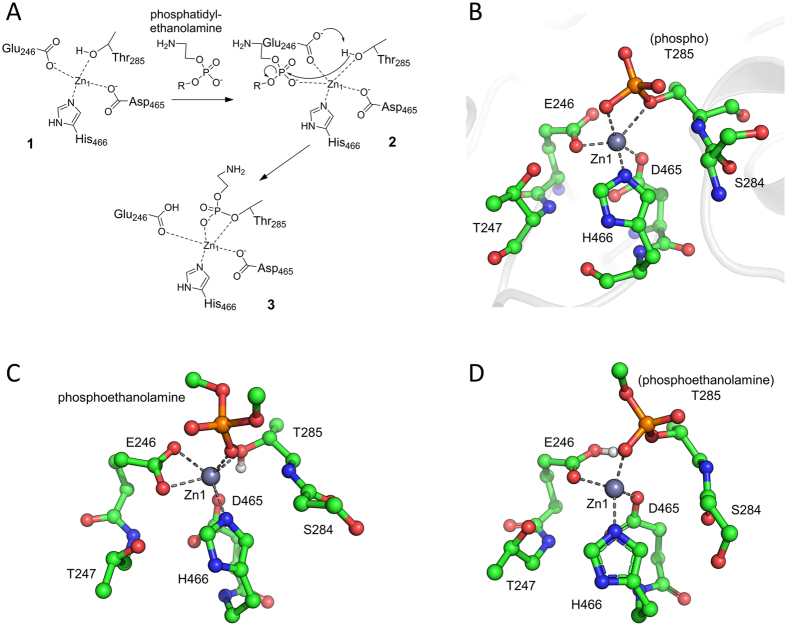
Density Functional Theory (DFT) Modelling of the MCR-1 Active Site (Mono-zinc form). (**A**) Possible mechanism for phosphoethanolamine addition (**2**) to MCR-1 Thr285 of mono-zinc MCR-1 (**1**) to form Thr285 adduct (**3**). (**B**) MCR-1 active site crystal structure (*P*2_1_ form) showing residues used in cluster model. (**C**) DFT-optimised geometry of phosphoethanolamine non-covalently bound to MCR-1 active site (**2** in panel (**A**) above). (**D**) DFT-optimised geometry of MCR-1 active site in the phosphoethanolamine-bound form (**3** in panel (**A**) above). (See Supplementary Information for full computational details and a discussion of results).

DFT calculations were performed on cluster models for the MCR-1 metal centre derived from the two structures, using simplified models, i.e. substituting [P(O)O(OMe)_2_]^−^ in the place of the phosphatidylethanolamine (substrate 1) and [P(O)(OMe)OO]^2−^ for the lipid A (substrate 2) substrates (procedures are fully described in Supplementary Information). Minimum-energy structures could be located for all intermediates along putative mechanistic pathways requiring either one (Fig. 4A) or two (Supplementary Figure S9A) zinc ions. For the mono-zinc mechanism, a model containing Glu246, Thr285, Asp465 and His466, with both Glu246 and Asp465 deprotonated, provided the best agreement with the experimental crystal structure (Supplementary Table S6). The coordination geometry observed about the zinc centre in this model is maintained throughout the postulated mechanism (Supplementary Table S7), indicating this to be structurally reasonable. Both Glu246 and Asp465 are geometrically accessible to act as general bases to activate Thr285, and the intermediates generated by proton transfer to Glu246 and Asp465 (1Zn-3E, 1Zn-D, Scheme S1, Supplementary Information) were isoenergetic within the limitations of the computational approach used (Supplementary Table S8), indicating that either residue may activate Thr285.

Similar to the mono-zinc mechanism above, the gas-phase optimised geometries for the di-zinc metal centre remain reasonable, and consistent with the crystal structure, around both zinc ions throughout the postulated reaction pathway (Supplementary Table S7), confirming that this putative mechanism can be accommodated by the crystallographically observed active site. However, unlike the case for the mono-zinc mechanism, activation of Thr285 by proton transfer to Asp465 resulted in a significant structural change in the active site that involves dissociation of this residue from the Zn1 metal ion. In contrast a smaller structural change was required for proton transfer to Glu246, and a transition state could be located for reaction of Glu246-activated Thr285 with the phosphate diester substrate 1. The relative potential energies (Supplementary Table S10) calculated for the di-zinc mechanism were considerably higher than those obtained for the mono-zinc reaction (Supplementary Table S8A), although there was considerable sensitivity to the solvation model and constraints used. Nevertheless, these initial calculations indicate that a mono-zinc mechanism for PEA transfer may be feasible, and demonstrate that the structures presented here can serve as suitable starting points for a more extensive computational exploration of MCR-1 mechanism.

## Discussion

The initial identification of *mcr-1* in an *E. coli* strain of animal origin in China has prompted extensive analyses of new and existing bacterial strain collections that have established this gene to have a wide geographic distribution in human, animal and environmental *E. coli*^9^. Given this, and the increasing importance of colistin as a last resort antibiotic for infections by multiresistant strains of GNB, we have sought to investigate the molecular basis for MCR-1 activity as a first step towards understanding its likely impact across the range of GNB and identifying possible routes to overcoming its activity. Although full elucidation of the means by which MCR-1 modifies bacterial LPS requires structural description of the complete protein, including the membrane-spanning domain, the high-resolution structures, and associated microbiological and computational data, that we have been able to obtain provide important information that permits some conclusions to be drawn regarding the structural basis for MCR-1 activity.

Consistent with previously recognised sequence relationships^8^, structure-based searches^23^ identify clear similarity of both overall fold and active site architecture between MCR-1 and other characterised bacterial PEA-transferases (*Neisseria meningitidis* LptA^15^ (catalytic domain, PDB 4KAV, 40% sequence identity, RMSD 1.9 Å over 302 Cα); *Campylobacter jejuni* EptC^13^ (catalytic domain, 4TN0, 41% sequence identity, RMSD 1.5 Å, 284 Cα)). This relationship further extends to other putative bacterial PEA-transferases as well as sulphatases and phosphatases (choline sulphatase (PDB 4UG4, RMSD 2.9, 270 Cα), arylsulphatase (3ED4, RMSD 3.2, 259 Cα), N-sulphoglucosamine sulphohydrolase^24^ (4MIV, RMSD 3.0, 262 Cα) and alkaline phosphatase (2×98, RMSD 3.0, 229 Cα)). Thus, like LptA and EptC, MCR-1 can be assigned as a member of the alkaline phosphatase (AP) metalloenzyme superfamily. Moreover, both comparisons with previous structures, and our observation of phosphorylation in one of our two structures, clearly identify Thr285 as the likely nucleophile and PEA acceptor in the catalytic mechanism.

Characterised AP enzymes vary in the nature and number of metal ions required for activity and, consequently, active-site architecture differs substantially between those of known structure^25^. Our structural comparisons identify the ligands to the MCR-1 Zn1 site, Asp465, His466, Glu246 and Thr285, to be well conserved between MCR-1, LptA and EptC and between MCR-1 and other AP family members, although the identity of the nucleophile differs between enzymes. However, less complete conservation is evident when comparing the putative Zn2 sites- while the Zn2 ligands His395 and His478, and the adjacent Glu468, are also well conserved between MCR-1, LptA and EptC, these positions vary in less closely related family members such as choline sulphatase (Supplementary Figure S7) suggesting that an intact Zn2 site is not a prerequisite for activity in all such enzymes. It is also notable that the Zn2 site is not occupied in the structure of EptC, while for LptA Zn2 was observed only after exposure to high concentrations of zinc^15^.

Similarly, a recently released structure of the MCR-1 catalytic domain (PDB 5K4P^20^, (Supplementary Figure S8), crystallised in the presence of 0.2 M ZnS0_4_, contains a total of 10 zinc ions bound to one MCR-1 molecule, including 4 in the active site pocket, and differs from the structures presented here specifically at the Zn2 site. While the overall folds of both the *P*2_1_ and *P*4_1_2_1_2 structures are very similar to that of 5K4P (RMSDs 0.286 Å (324 Cα) and 0.432 Å (311 Cα), respectively), only the Zn1 site is similar in all three structures (Supplementary Figure S8). In 5K4P additional zinc ions in the Zn2 site (ZnB, ZnC and ZnD) are coordinated by His395 and the phosphate group of phospho-Thr285 (ZnB); by a network of water molecules (ZnC); and His478 and Glu405 from an adjacent molecule in the crystal lattice (ZnD). In comparison to the phosphorylated *P*2_1_ structure (Supplementary Figure S8) additional zinc binding causes rotation of the Thr285 phosphoryl group and loss of interaction of His478 with the phosphoryl-oxygen. This also results in a shift in the position of ZnD in comparison to Zn2 in our MCR 1-*P*4_1_2_1_2 structure (Supplementary Figure S8). These comparisons indicate first that the conformations of active site residues in MCR-1 are sensitive to conditions of high zinc content (as in 5K4P), demanding caution when making any mechanistic interpretation, and, more importantly, that available structural data do not clearly define a unique Zn2 site in MCR-1. Taken together, these data make uncertain the role of this metal ion in the PTE transfer reaction. Nevertheless, the effect of alanine substitutions at His395 and His478 on colistin susceptibility of producer strains indicates that, irrespective of their role in possible metal binding, both of these residues are important to the structure or activity of MCR-1. Similar effects were observed for the equivalent mutations of EptC^13^. This conclusion is strengthened by the observation that mutation of Glu468, a residue positioned to interact with the His478 side chain and whose functional role has to date not been investigated, is also deleterious to MCR-1 function.

Notwithstanding the differing metal contents of MCR-1 structures, the presence of zinc in a conserved site adjacent to phosphorylated Thr285 provides strong evidence for its importance to MCR-1 function. This conclusion is supported by microbiological experiments in which the effect of the chelator EDTA upon colistin MIC was investigated for a collection of *E. coli* strains. These included samples from hospitalised human patients (China) and healthy human volunteers (Thailand); faeces from chickens (China, Thailand) and other farmed and domestic animals (Thailand); and the wider environment (primarily water sources (reservoirs, rivers, canals; Thailand; Supplementary Table S3)). For MCR-1-positive strains clear reductions in colistin MIC were observed on EDTA treatment, supporting the conclusion that zinc is required for MCR-1 activity in the host. Interpretation of these experiments could be complicated by the known effect of chelators such as EDTA upon the Gram-negative outer membrane. EDTA treatment sensitises *E. coli* and other GNB to colistin, as well as a range of antibiotics that fail to penetrate Gram-negative cells, most likely due to sequestration of bound magnesium ions that normally hold together LPS within the outer membrane^26^. While we cannot entirely rule out such effects, control experiments support the contention that the observed differences in colistin susceptibility relate to loss of MCR-1 activity rather than non-specific changes in outer membrane integrity. Specifically for MCR-1 negative *E. coli* with elevated colistin MICs (0.5 or 1.0 μg/ml), EDTA reduced these values by at most one dilution, compared to changes of up to five dilutions in MCR-1 positive isolates, suggesting that in these strains non-specific effects alone cannot account for the observed differences. These results justify further evaluation of this approach as a possible route to phenotypic detection of MCR-1 production in *E. coli*.

AP mechanism has been extensively studied for a number of family members, and, prior to this study, crystal structures were available for two known PEA-transferases (LptA and EptC). However, to date the catalytic mechanism of these enzymes, or other known PEA-transferases of this family, has been little explored. Accordingly, as an initial investigation of the mechanism of PEA transfer, we used DFT to construct a series of models representing possible states along the PEA transfer pathway catalyzed by MCR-1 in both mono- and di-zinc forms. Interestingly, while plausible structural models could be generated for states along both of these pathways, energies for species along the mono-zinc pathway were consistently lower than those obtained for the di-zinc mechanism. While calculated energies remained sensitive to the model setup (for details see Supplementary Information), these data support the hypothesis that a single zinc ion may be sufficient to support at least the first stage of the MCR-1 catalysed reaction, i.e. PEA transfer onto Thr285. Importantly, the crystal structures that we present here will facilitate the more extensive computational studies required to address this and other mechanistic proposals. In the AP catalysed reaction, the likely role of the second (Zn2) zinc ion is to orient the incoming substrate for attack by the nucleophile (MCR-1 Thr285), stabilising the resulting transition state and activating an incoming water molecule that degrades the covalent intermediate^27^. Mono-zinc family members such as phosphonate monoester hydrolase (PMH) can fulfil some of these functions by substitution of basic residues for zinc in the absence of a second metal site^28^. Thus, it is plausible that MCR-1 and other LPS PEA-transferases function with a single zinc equivalent. However, we cannot rule out the previously raised possibility^15^ that substrate binding, particularly of the lipid A acceptor, provides the extra coordination necessary to bind an additional zinc ion and that a second metal is required to complete the PEA transfer reaction.

In summary, our data provide near-atomic resolution structures of the MCR-1 catalytic domain that define the architecture of the protein and identify key features of its activity. Zinc, disulphide bonds and conserved active site residues, including zinc ligands, the acceptor Thr285 and additional positions adjacent to the metal centre, are all important to MCR-1 function in *E. coli*. In particular, investigation of the effect of EDTA on colistin susceptibility in producer strains supports this approach as potentially suitable for phenotypic discrimination of MCR-1 producing *E. coli*. DFT calculations indicate that, in contrast to the majority of alkaline phosphatase (AP) type enzymes, MCR-1 may require only a single zinc equivalent to catalyse the PEA transfer reaction. These data identify MCR-1 (and other related bacterial PEA-transferases) as a distinct subset within the AP enzyme family and provide starting points for more extensive investigations of MCR-1 mechanism and inhibition, in particular for structure-based design of inhibitors for use in potential colistin-based combination therapies.

## Methods

### Expression of the MCR-1 catalytic domain

To express the soluble domain of MCR-1 lacking the five predicted transmembrane (TM) helices^8^ (MCR1ΔTM), codons 219–541 were synthesised (Eurofins) and subcloned into the pOPINF^29^ T7 expression vector with forward and reverse primers (MCR-1^soluble^ F and MCR-1^soluble^ R, Supplementary Table S5)). The resultant plasmid, pOPINF-MCR1ΔTM, encodes for N-terminally His_6_-tagged protein cleavable with 3 C protease. *E. coli* SoluBL21 (DE3) cells (Genlantis) bearing pOPINF-MCR1ΔTM were grown at 37 °C in 2xTY media containing 50 μg ml^−1^ carbenicillin to A_600_ 0.6, when IPTG was added to a final concentration of 0.5 mM, and cell growth continued for 18 h at 18 °C. Cells harvested by centrifugation (6500 × *g*, 10 min) were resuspended in 50 mM HEPES pH 7.5, 500 mM NaCl, 2 mM β-mercaptoethanol, 0.1 mM ZnCl_2_, EDTA-free protease inhibitor (Roche), and broken by two 30 000 psi passages through a cell disruptor. All subsequent steps were performed at 4 °C. Unbroken cells were removed by ultracentrifugation at 100,000 × g for 1 h and supernatant (plus 10 mM imidazole) incubated 2 h with Ni-NTA resin (Qiagen). Protein-bound resin was washed in Buffer A (50 mM HEPES pH 7.5, 400 mM NaCl, 0.1 mM ZnCl_2_, 1 mM TCEP) plus 10 mM imidazole then in Buffer A plus 20 mM imidazole, and protein eluted in 50 mM HEPES pH 7.5, 200 mM NaCl, 10 μM ZnCl2, 400 mM imidazole, 1 mM TCEP. Imidazole was reduced to 10 mM in an Amicon 10-kDa centrifugal filter. The tag was removed by 3 C protease cleavage overnight and capture on Ni-NTA resin. Protein was subsequently loaded onto a Superdex 75 column equilibrated in 50 mM HEPES pH 7.5, 150 mM NaCl, 100 μM ZnCl_2_. Peak fractions were concentrated to 16 mg/mL.

### Crystallisation, Structure determination and Analysis

The MCR-1 soluble domain was crystallised by sitting drop vapour diffusion at 18 °C in MRC 96-well crystallisation plates (Molecular Dimensions). *P*4_1_2_1_2 crystals formed by mixing 400 nL protein with 200 nL crystallisation reagent [0.1 M MOPS/HEPES-Na pH 7.5, 10% w/v PEG 20000, 20% v/v PEG MME 550, 0.02 M 1,6-hexanediol, 0.02 M 1-butanol, 0.02 M 1,2-propanediol, 0.02 M 2-propanol, 0.02 M 1,4-butanediol, 0.02 M 1,3-propanediol]. *P*2_1_ crystals formed by mixing 300 nL protein with 600 nL crystallisation reagent (0.2 M Lithium chloride, 0.1 M sodium acetate pH 5.0, 20% w/v PEG6000). Drops were equilibrated against 50 μl crystallisation reagent. Crystals grew to maximum size in 14 days. For freezing, crystals were transferred into cryoprotectant (reservoir solution plus 20% glycerol) before harvesting with a cryo-loop and flash-freezing in liquid nitrogen.

Diffraction data were collected at 100 K on beamlines I03 (Zn-edge datasets) and I04–1 or I02 (native datasets) at Diamond Light Source, UK. An X-ray fluorescence scan around the Zn-edge (9626 to 9685 eV) clearly showed a peak corresponding to the presence of zinc in the crystal. The structure was therefore determined using the Single-wavelength Anomalous Dispersion (SAD) method by combining four 2.5 Å resolution isomorphous datasets collected at the Zn-edge (wavelengths in Table S1). The datasets were indexed and integrated using XDS^30^ and combined and scaled using Aimless^31^ in CCP4^32^. Experimental phases, a density modified map and an initial model were calculated using AutoSol^33^ in Phenix^34^, with one molecule in the asymmetric unit and two zinc sites identified. Figure of merits were 0.31 and 0.66, before and after density modification. The model was further built using AutoBuild^35^ in Phenix. This initial structure was then used as a search model for molecular replacement with Phaser^36^ to phase a 1.75 Å resolution P4_1_2_1_2 dataset indexed and integrated using DIALS, as implemented in Xia2^37^, and scaled and merged in Aimless. The 1.55 Å resolution *P*2_1_ native dataset was processed using the Xia2 pipeline (indexed and integrated using XDS and scaled using Aimless). Phases were calculated by molecular replacement with Phaser, using the *P*4_1_2_1_2 structure as a starting model. Structures were completed in both cases by iterative rounds of manual model building in Coot^38^ and refinement in Phenix. Structure validation was assisted by Molprobity^39^ and Phenix.

Co-ordinates and structure factors have been deposited with the Protein Data Bank (www.rcsb.org/pdb) with accession numbers 5LRM and 5LRN.

### Analytical Size Exclusion Chromatography

Purified protein used for crystallographic studies was analysed by size exclusion chromatography. Protein was loaded on to a Superdex 75 column equilibrated in 50 mM HEPES pH 7.5, 150 mM NaCl, 100 μM ZnCl_2_. Elution volume was compared against the elution volume of standards (GE Healthcare) to determine molecular weight. Column void volume was determined by loading blue dextran 2000.

### Mass Spectrometry of Purified Recombinant MCR-1

Mass spectrometry measurements were carried out as previously described^40^. Briefly, electrospray ionization (ESI) -spectra were acquired in the positive ion mode using a Waters LCT Premier instrument equipped with a TOF analyser. An LCT Premier mass spectrometer (Waters) was coupled to an Agilent 1100 Series HPLC using a Chromolith^®^ FastGradient RP-18 endcapped column equipped with a 50-2 HPLC column, made of monolithic silica (C18, 2 × 50 mm, macropores with 1.6 μm diameter, Merck). The instrument was connected to a CTC-autosampler inlet system. A multi-step gradient over 10 min was run (solvent A 94.9% H_2_O/5% CH3CN/0.1% formic acid, solvent B 99.9% CH_3_CN/0.1% formic acid; 0-1 min 5% B for equilibration, followed by a linear gradient to 100% B over 4 min, then 100% B for an additional 3 min, followed by a linear gradient over 2 min back to 5% B to re-equilibrate the column) to separate the protein samples at flow rates of 0.4 ml/min for the first 5 min and then 1.0 ml/min for the remaining time. The electrospray ionisation source used a capillary voltage of 3.2 kV and cone voltage of 25 V. Nitrogen was used as the nebuliser and desolvation gas at a flow rate of 600 l/h. Protein typically eluted as a peak between 3 and 5 min under these conditions. Calculated masses were obtained using the ExPasy ProtParam tool (http://web.expasy.org/protparam/).

### Construction of MCR-1-expressing Plasmid for Susceptibility Testing

Susceptibility testing was carried out on full-length MCR-1 constitutively expressed from the pUC19 vector^41^. The *mcr-1* coding region with its own promoter (156 bp) and downstream sequence (111 bp) was PCR amplified from plasmid pHNSHP45^8^ using Q5 High-Fidelity DNA Polymerase (New England Biolabs) and mcr-1 1893 F and mcr-1-1893 R (Supplementary Table S5). The fragment was phosphorylated, gel purified, digested with XbaI and EcoRI and cloned into XbaI and EcoRI-digested pUC19 to create plasmid pUC19-mcr-1. The integrity of the construct was confirmed by DNA sequencing.

### Construction of MCR-1 Site-Directed Mutants

MCR-1 mutants were generated in pUC19-mcr-1 using the QuikChange II XL Site-Directed mutagenesis kit (Agilent Genomics) with a modified protocol (reaction cycles increased to 21) and primers as specified in Supplementary Table S5.

### Co-expression of MCR-1 and *E. coli* DsbA

The *mcr-1* containing fragment was excised from plasmid pUC-19-mcr-1 by double digestion with EcoRI and XbaI and ligated into plasmid pSU18^42^ to generate plasmid pSU18-mcr-1. pSU18-mcr-1 was transformed into *E. coli* TOP10 (Invitrogen), purified and its integrity verified by double restriction digestion and DNA sequencing. A 627 bp fragment corresponding to the *dsbA* open reading frame from *E.coli* MG1655 (NCBI reference sequence: NC_00913.3) was synthesised (Thermo-Fisher). The fragment was inserted into pBAD-HisA (Invitrogen) using the HindIII and NcoI restriction sites to create plasmid pBAD-dsbA which was transformed into *E. coli* TOP10 (Invitrogen). pBAD-dsbA was purified from transformants and its integrity verified by double restriction digestion and DNA sequencing. Plasmids pSU18-mcr-1 and pBAD-dsbA were transformed into *E. coli* TOP10 and recombinants selected on LB agar plates containing chloramphenicol (25 mg/L) and ampicillin (100 mg/L). The plasmid complement of transformants was verified by PCR and double restriction digestion. Cells containing plasmids pSU18-mcr-1 and pBAD-dsbA were tested for colistin susceptibility by agar dilution as described below.

### Antimicrobial susceptibility Testing

#### Agar Dilution Method

Minimal inhibitory concentrations (MICs) of colistin for *E. coli* TOP10 cells expressing wild-type MCR-1 and mutants in pUC19, and co-expressing MCR-1 and *E. coli* DsbA, were assayed by the agar dilution method, according to the European Committee on Antimicrobial Susceptibility Testing (EUCAST). Briefly, bacterial suspensions of optical density corresponding to a 0.5 McFarland Standard (approximately 1.5 × 10^8^ CFU/mL) were prepared for the tests and used to inoculate Mueller-Hinton (Becton Dickinson, USA) agar plates supplemented with colistin at concentration ranges of 0.125–128 mg/L. *E.coli* strain ATCC 25922 was used for quality control purposes. Two independent experiments, each with samples in duplicate, were carried out.

#### Broth Microdilution Method

The effect of EDTA upon the colistin susceptibility of laboratory, environmental, veterinary and clinical *E. coli* isolates was assessed by broth microdilution according to the European Committee on Antimicrobial Susceptibility Testing (EUCAST). Details of bacterial strains used in these experiments are given in Table S3. Briefly, cells were resuspended to an optical density corresponding to a 0.5 McFarland standard (approximately 1.5 × 10^8^ CFU/mL) and used to inoculate 96-well microtitre plates containing cation-adjusted Mueller-Hinton medium (Becton Dickinson, USA) at colistin concentrations from 0.25 to 128 μg/mL. EDTA was added to experimental wells to a final concentration of 250 μg/ml EDTA. Plates were incubated overnight at 37 °C and optical density read on a plate reader at 492 nm. Experiments were carried out in duplicate; for strains isolated in China (Table S3) the experiment was repeated three times.

### Density Functional Theory Calculations

Starting geometries were generated from X-ray crystal structures by inclusion of residues within approximately 5–10 Å of the zinc ions, and subsequent manual reduction to include relevant residue side chains and loops. Residues were truncated at an alpha carbon and saturated with hydrogen atoms; hydrogen atoms were also added manually to satisfy the valencies of all other atoms in the model. Optimisations were performed using six cartesian constraints on alpha carbons for intermediates in which His395 and His478 were included and four where they were excluded. All optimisations were performed using Jaguar version 8.5^43^, with the B3LYP hybrid functional^44^,^45^,^46^,^47^ using loose convergence criteria. Test calculations on other complexes using the more stringent default convergence criteria did not lead to significant changes in energies, bond lengths, or angles, but were much more time-consuming. A relativistic effective core potential was used for the zinc atom(s) as incorporated into Jaguar’s LACV3P* basis set, and the Pople 6–31 G* basis set for all other atoms. Energies were calculated using single point calculations incorporating the Poisson–Boltzmann finite-element model of solvation^48^,^49^ as implemented in Jaguar. The dielectric constant was set to 4, as commonly used in the cluster model approach^50^ and the probe radius to 2 Å. Vibrational frequencies were not computed, and so the energetic data do not include a correction for zero-point energy, although we note that this would be expected to be quite small. In the absence of frequency calculations, stationary points have not been verified as minima.

#### Code Availability

Jaguar version 8.5 is available from Schrodinger, Inc., New York, NY. See https://www.schrodinger.com/jaguar for details.

#### Data Access Statement

Crystal structures are available from the Protein Data Bank (www.rcsb.org/pdb) with accession numbers 5LRM and 5LRN. All other data supporting this study are provided as Supplementary Information accompanying this paper.

## Additional Information

**How to cite this article**: Hinchliffe, P. *et al*. Insights into the Mechanistic Basis of Plasmid-Mediated Colistin Resistance from Crystal Structures of the Catalytic Domain of MCR-1. *Sci. Rep.* **7**, 39392; doi: 10.1038/srep39392 (2017).

**Publisher's note:** Springer Nature remains neutral with regard to jurisdictional claims in published maps and institutional affiliations.

## Supplementary Material

Supplementary Information

## Figures and Tables

**Figure 1:**
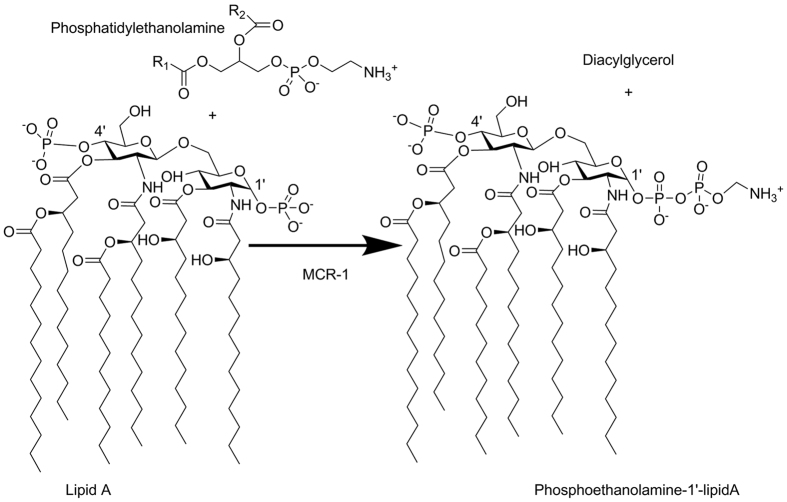
Phosphoethanolamine Transfer Reaction Catalysed by MCR-1. Panel shows hexa-acylated *E. coli* lipid A structure^51^ with 1′ and 4′ phosphate groups. MCR-1 catalyses transfer of phosphoethanolamine from a phosphatidylethanolamine donor substrate onto lipid A. Figure shows addition of phosphoethanolamine to the 1′ position of lipid A, consistent with the proposed activity of *Salmonella* PmrC^14^.
