# Supplementary material for: Insights into the Mechanistic Basis of Plasmid-Mediated Colistin Resistance from Crystal Structures of the Catalytic Domain of MCR-1
Source: Sci Rep. 2017 Jan 6;7:39392. doi: 10.1038/srep39392 (PMC5216409; doi:10.1038/srep39392)
Supplement: Supplementary Information [file srep39392-s1.pdf]

Supplementary Information for

Insights into the Mechanistic Basis of Plasmid-Mediated Colistin Resistance from the Crystal Structure of the Catalytic Domain of MCR-1

Philip Hinchliffe, Qiu E. Yang, Edward Portal, Tom Young, Hui Li, Catherine L. Tooke, Maria J. Carvalho, Neil G. Paterson, Jürgen Brem, Pannika R. Niumsup, Uttapoln Tansawai, Lei Lei, Mei Li, Zhangqi Shen, Yang Wang, Christopher J. Schofield, Adrian J Mulholland, Jianzhong Shen, Natalie Fey, Timothy R. Walsh and James Spencer.

Figures

Supplementary Figure S1. Superposition of the $P4_12_12$ and $P2_1$ MCR-1 structures.

Supplementary Figure S2. The MCR-1 crystallographic dimer.

Supplementary Figure S3. X-ray fluorescence scan of MCR-1 crystals.

Supplementary Figure S4. Mass Spectrum of the soluble MCR-1 domain used in crystallographic studies.

Supplementary Figure S5. Hydrophobicity and charge distribution over MCR-1.

Supplementary Figure S6. Colisitin concentration-killing curves against bacterial isolates.

Supplementary Figure S7. Active site architecture of MCR-1 compared with structurally related enzymes.

Supplementary Figure S8: Comparison of mono and di-zinc MCR-1 crystal structures with the multi-zinc MCR-1 structure 5K4P.

Tables

Supplementary Table S1. Data collection and refinement statistics

Supplementary Table S2. Zinc-coordination distances in Å

Supplementary Table S3. Details of Bacterial Strains used in Minimal Inhibitory Concentration (MIC) Experiments

Supplementary Table S4. Colistin MICs of *E. coli* TOP10 Expressing *mcr-1* Mutants

Supplementary Table S5. Primers used in this study

Computational Details

DFT Calculation Results

Including:

Supplementary Figure S9: Density Functional Theory (DFT) Modelling of MCR-1 Active Site (Di-Zn(II) form).

Supplementary Table S6a: Structural comparison of Phosphorylated mono-Zn(II) active site geometries (see later discussion of possible protonation states, Scheme S1 for labelling used).

Supplementary Table S6b. Structural comparison of Di-Zn(II) active site geometry (see Scheme S2 for details of intermediates).

DFT analysis of the mono-Zn(II) mechanism

Scheme S1: Mechanistic postulate for mono-Zn mechanism. 1Zn-2 to 1Zn-3D and 1Zn-3E proceed via transition states 1Zn-$TS1_D$ and 1Zn-$TS1_E$ respectively.

Supplementary Table S7: Key structural parameters for the mono-Zn mechanism active site (distances given in Å).

Supplementary Table S8: Calculated relative potential energies in kcal mol^{-1} for mono-Zn mechanism shown in Scheme S1.

DFT analysis of the Di-Zn(II) mechanism

Scheme S2: Mechanistic postulate for di-Zn mechanism. 2Zn-2 to 2Zn-3D and 2Zn-3E proceed via transition states 2Zn-$TS1_D$ and 2Zn-$TS1_E$ respectively.

Supplementary Table S9: Important structural parameters for the di-Zn mechanism active site (distances given in Å, see Scheme S2 for atom labelling).

Supplementary Table S10: Calculated relative potential energies in kcal mol^{-1} for di-Zn mechanism shown in Scheme S2

References

Data

Coordinates for all DFT-optimised geometries

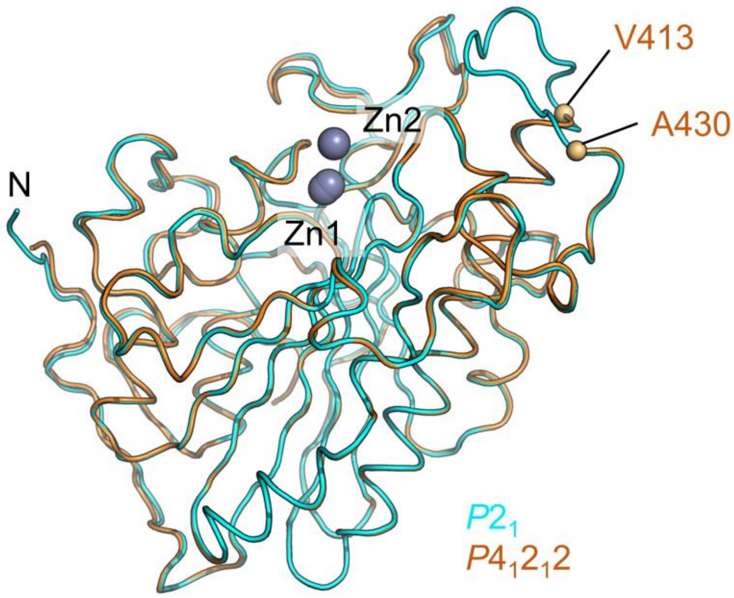

Supplementary Figure S1. Superposition of the $P4_12_12$ and $P2_1$ MCR-1 structures.

The $P4_12_12$ MCR-1 monomer (orange) is superposed to chain A of the $P2_1$ monomer (cyan). The N-termini are labelled and Zinc ions are shown as grey spheres and labelled Zn1 (common to both $P2_1$ and $P4_12_12$ structures) and Zn2 ($P4_12_12$ only). Residues between V413 and A430 in the $P4_12_12$ monomer could not be modelled due to poorly-defined electron density, while in the $P2_1$ structure this loop could be modelled into well-defined electron density.

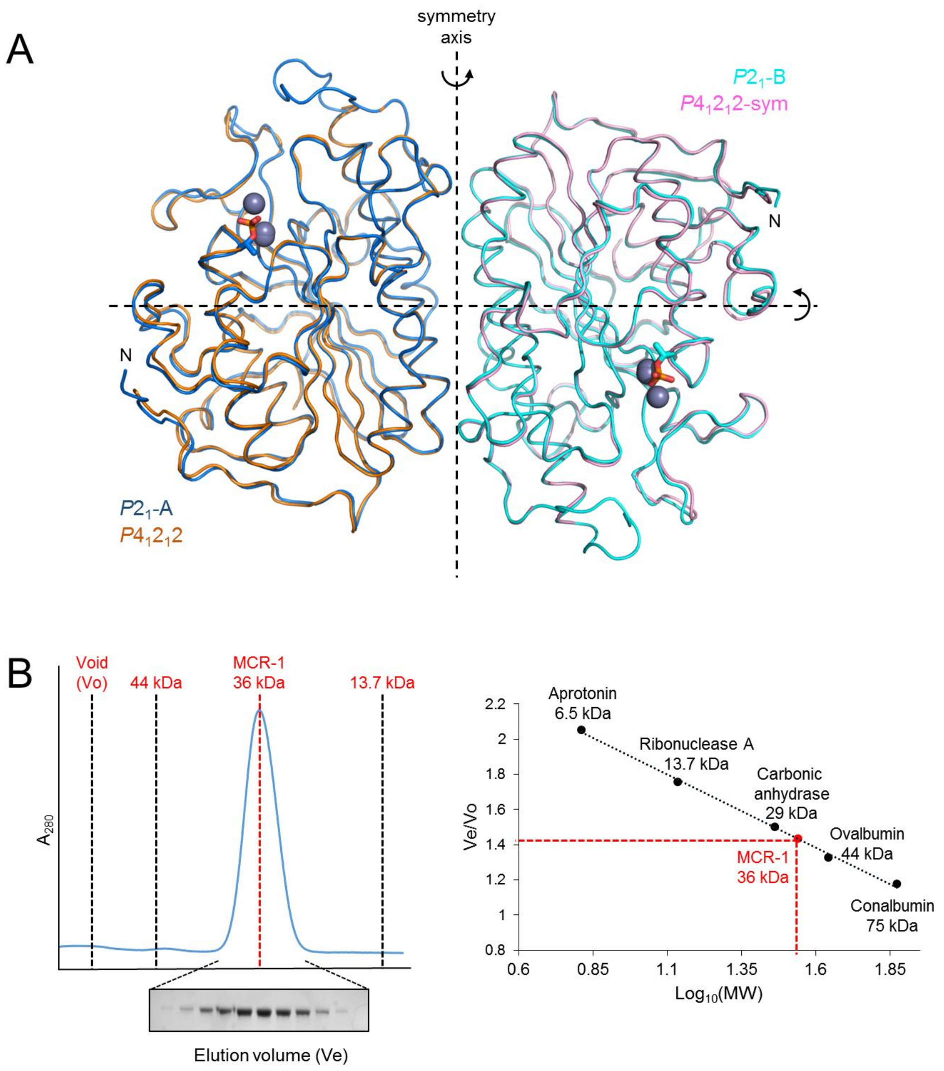

Supplementary Figure S2. The MCR-1 crystallographic dimer.

(A) Superposition of the *P*2$_1$ asymmetric unit (ASU) dimer (blue and cyan, chain A and B, respectively) with a dimer formed in the *P*4$_1$2$_1$2 crystal lattice (orange and pink for the ASU monomer and the symmetry related molecule, respectively). The two four-fold symmetry related dimers are essentially identical (RMSD=0.48 over 621 Cα). The Zinc atoms (grey spheres) and phosphothreonine residue (sticks) indicate the position of the active site.

(B) Size exclusion chromatography of purified MCR-1 catalytic domain. *Left*, absorption (280 nm) is shown in blue, with the peak marked by a red line. SDS-PAGE analysis of peak fractions is shown below. Dotted lines indicate the elution volume of the void and two standards (ovalbumin, 44 kDa and ribonuclease A, 13.7 kDa). The experiment was performed three times; data from a representative experiment are shown. *Right*, Calibration plot. The Log$_{10}$ of five molecular weight standards (black circles) is plotted against Ve/Vo (elution volume divided by column void volume). The line of best fit (R^2 = 0.998) was then used to calculate the apparent molecular weight of MCR-1 (red circle). This showed that the catalytic domain of MCR-1 elutes at an apparent molecular mass of 36 kDa, consistent with the monomeric form.

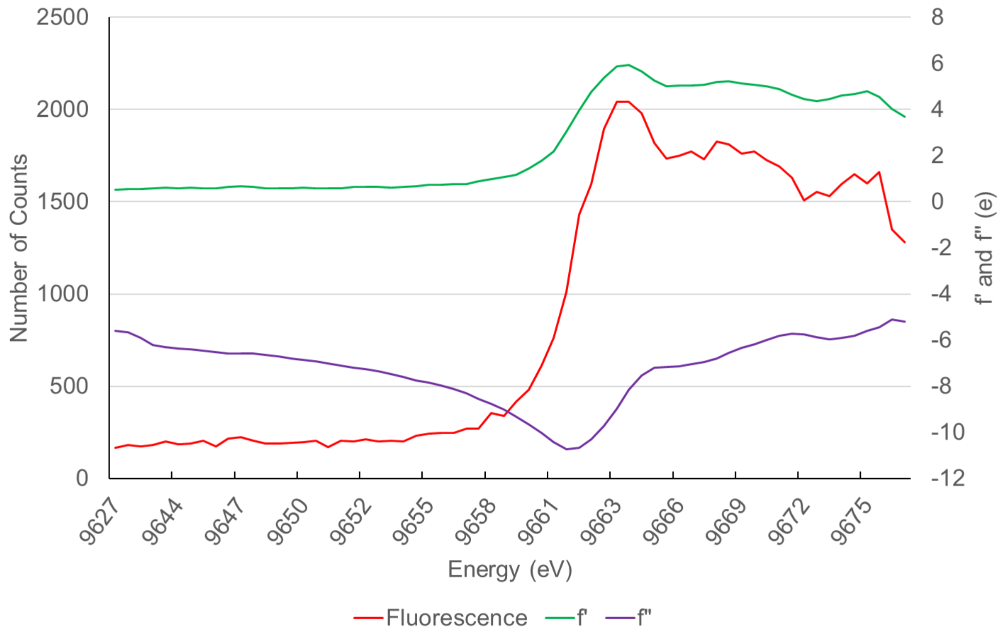

Supplementary Figure S3. X-ray fluorescence scan of MCR-1 crystals.

MCR-1 crystals ($P4_12_12$ form) were washed in zinc-free cryoprotectant before being looped and flash frozen in liquid nitrogen. The crystal was scanned (Diamond beamline I03) between the energies 9626 and 9685 eV. Measured fluorescence is shown as a red line and f' and f'' values are shown as green and purple lines, respectively. The peak was measured at 9664.040039 eV (f': 5.92 / f'': -8.15 e), consistent with a zinc signal. The scan was measured once from the single crystal used for multiwavelength data collection (see Supplementary Table 1 for details).

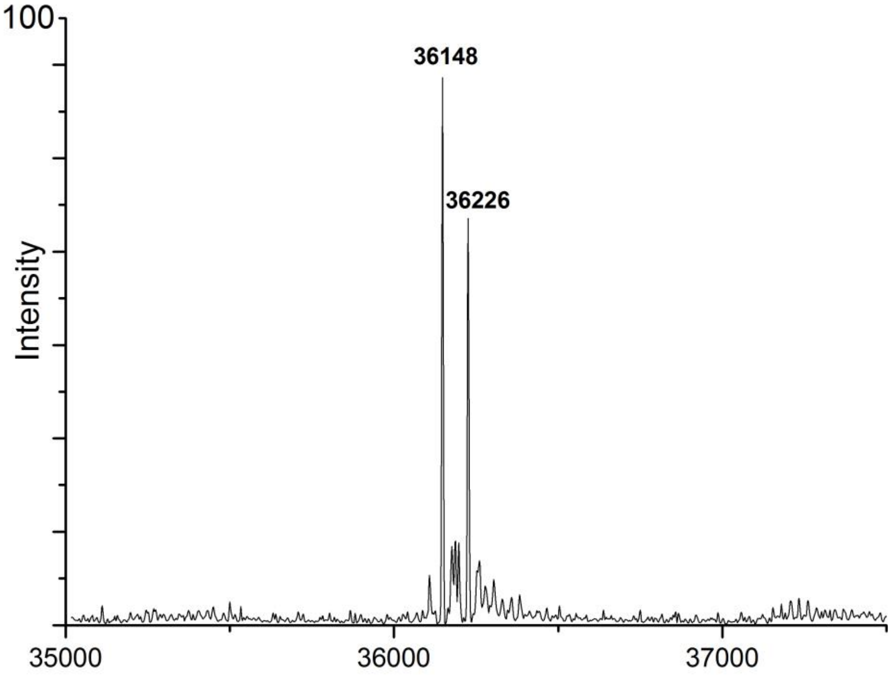

Supplementary Figure S4. Mass Spectrum of the soluble MCR-1 domain used in crystallographic studies.

The electrospray ionisation mass spectrometry (ESI-MS) spectrum of MCR-1 used in crystallographic studies revealed two peaks at 36148 Da and 36226 Da consistent with native (predicted mass from sequence 36 152 Da) and auto-phosphorylated protein, respectively. The experiment was performed three times; data from a representative experiment are shown.

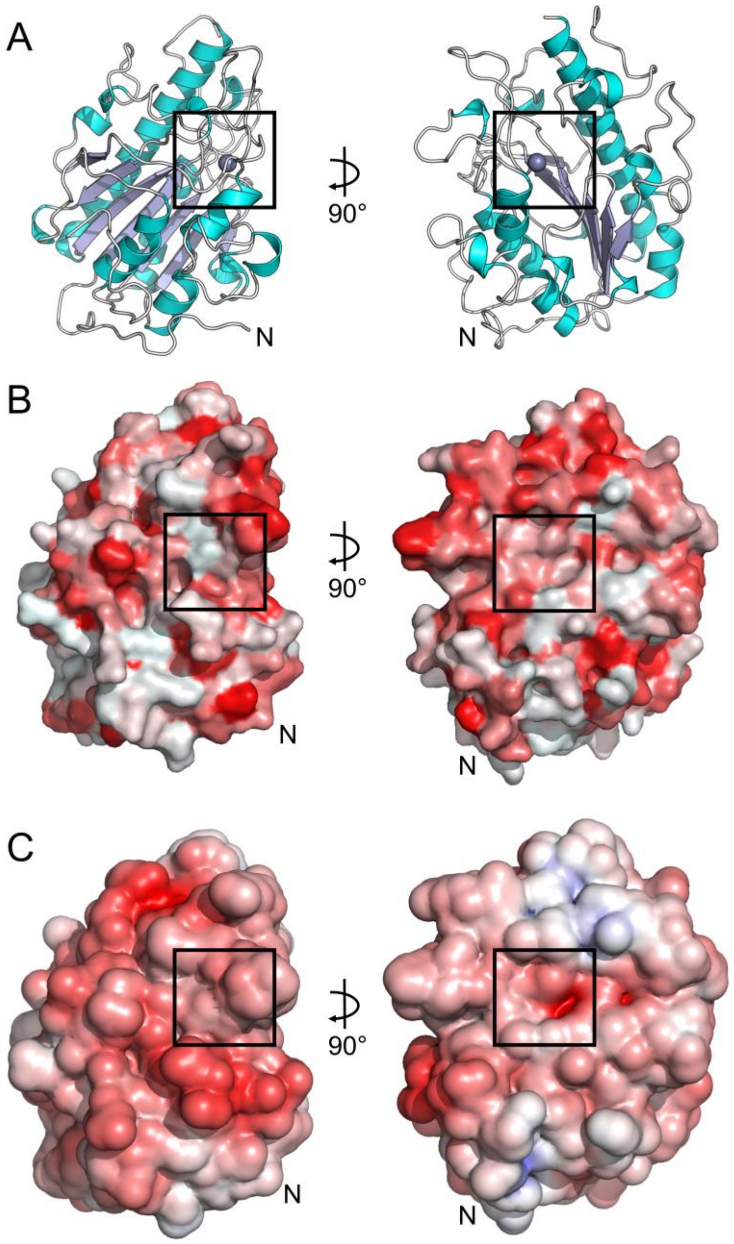

Supplementary Figure S5. Hydrophobicity and charge distribution over MCR-1.

(A) Two views (rotated by 90°) of the $P2_1$ structure of MCR-1, coloured as in main text Figure 2. The black box denotes the position of the active site.

(B) Hydrophobicity of the surface residues of the MCR-1 catalytic domain. View in the same orientation as (A) with the surface coloured white (hydrophilic) to red (hydrophobic) based on the Eisenberg hydrophobicity scale[1].

(C) Charge distribution over the surface of the MCR-1 catalytic domain. View in the same orientation as (A) with the surface shown with positively and negatively charged areas coloured blue and red, respectively. Electrostatic surface potentials were calculated using the program APBS[2] and contoured at ± 8 kT/*e*.

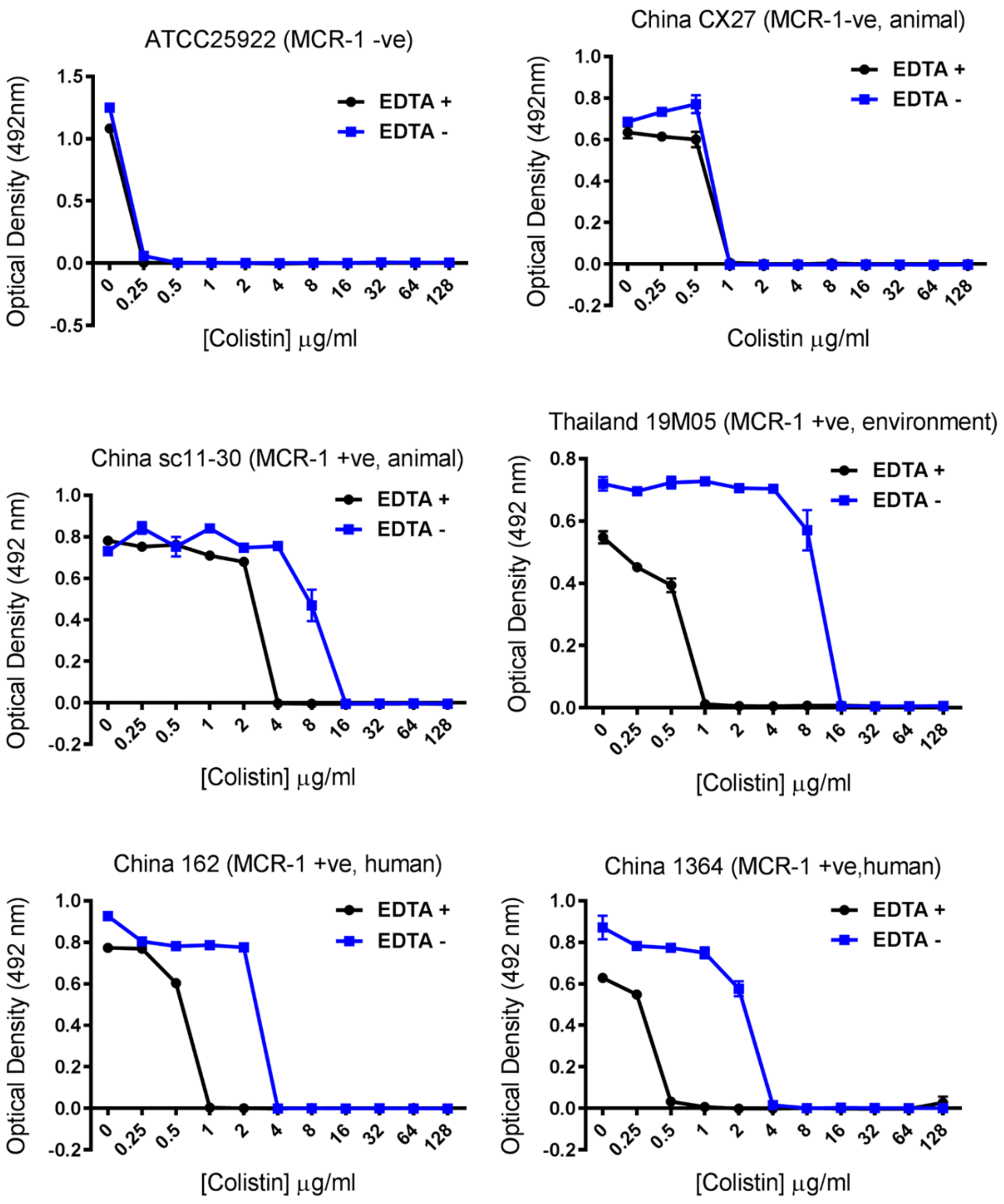

Supplementary Figure S6. Colisitin concentration-killing curves against bacterial isolates.

Effect of colistin concentration in the presence or absence of EDTA on the growth of representative *E. coli* strains either harbouring (MCR-1 +ve) or lacking (MCR-1 -ve) MCR-1. Data shown are from experiments performed in duplicate. Error bars represent standard deviations.

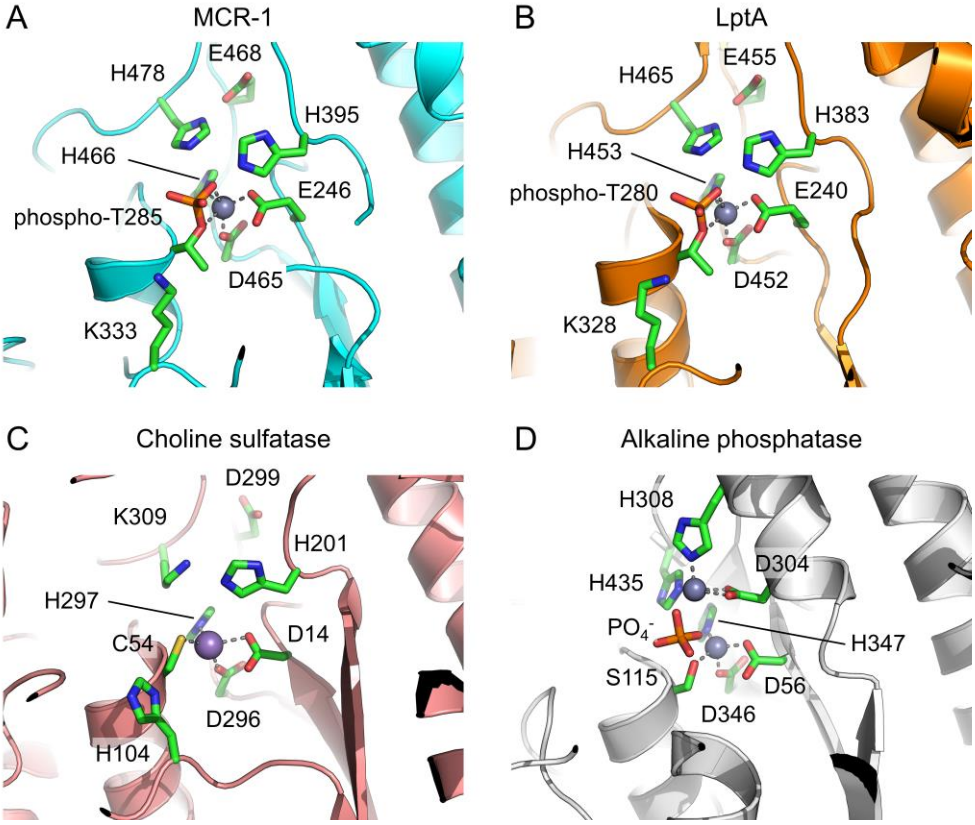

Supplementary Figure S7. Active-site architecture of MCR-1 compared with structurally related enzymes.

Representative structures from three different enzyme classes were superposed upon MCR-1 using secondary structure matching, and the active site of each is shown in the same orientation. Metals are shown as grey spheres and key active site residues (green sticks) are labelled. Metal coordination bonds are shown as grey dashes. Residues are numbered according to the respective PDB files.

(A) Mono-zinc MCR-1 (cyan, $P2_1$ form with phosphothreonine).

(B) Phosphoethanolamine transferase LptA from *Neisseria meningitidis* (PDB ID 4KAV[3], orange; monozinc, phosphothreonine form). RMSD to MCR-1 is 1.9 Å over 302 Cα residues, with 40% sequence identity. MCR-1 residues highlighted in (A) are all strictly conserved in LptA.

(C) Choline sulfatase from *Sinorhizobium melliloti* (PDB ID 4UG4, light red). RMSD to MCR-1 is 2.9 Å over 270 Cα residues with 16% sequence identity. The structure contains a single manganese site coordinated by C54, D14, D296 and H297. H201, K309, D299, and H104 structurally align with the MCR-1 residues H395, H478, E468, K333, respectively.

(D) Alkaline phosphatase from *Halobacterium salinarum* (PDB ID 2X98[4], grey). RMSD to MCR-1 is 3.0 Å over 229 Cα residues with 12% sequence identity. A single zinc site is coordinated by S115, D346, D56 and H347 and a free phosphate group (PO_4^-). The second zinc site (coordinated by H308, H435 and D304) is structurally distinct from the second zinc site of MCR-1 which is coordinated by H478 and H395 (see main text Figure 2C).

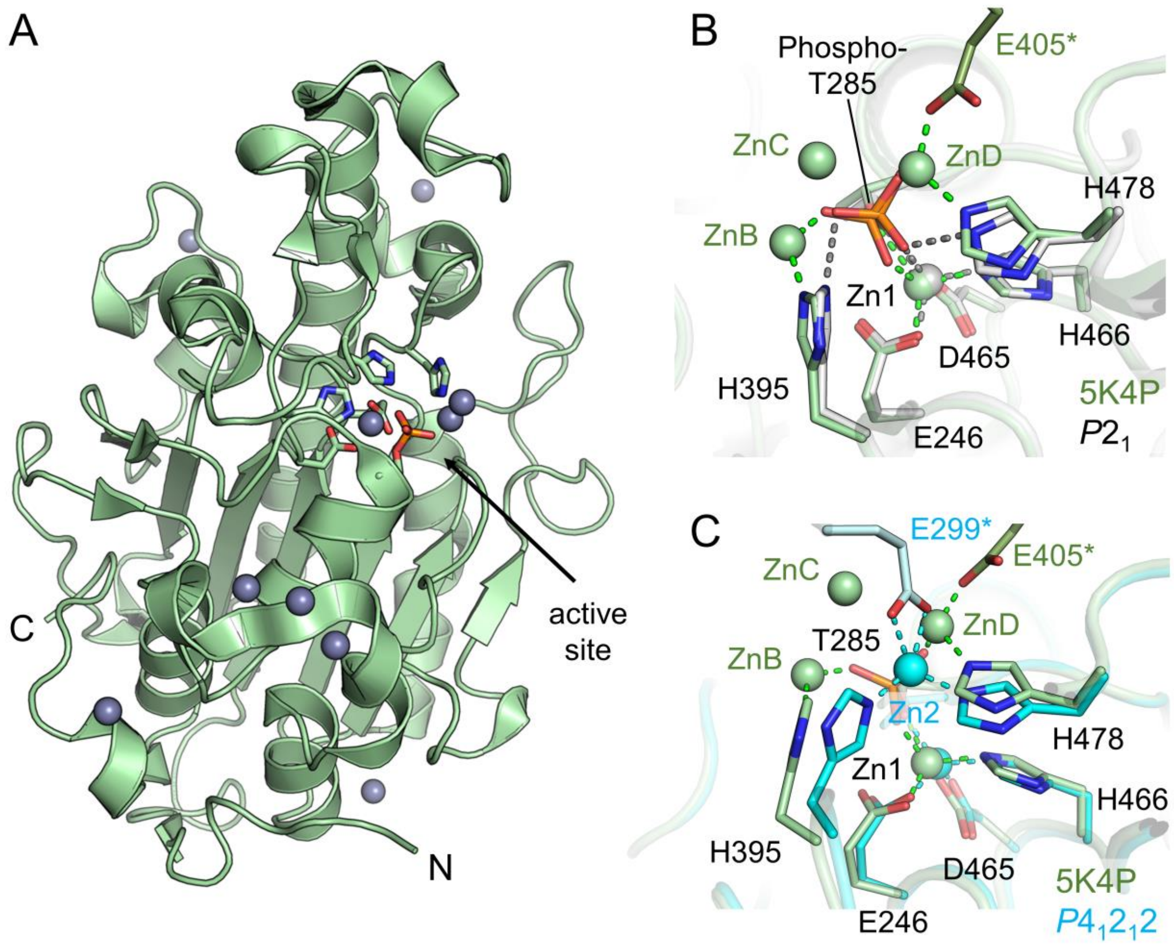

Supplementary Figure S8: Comparison of mono and di-zinc MCR-1 crystal structures with the multi-zinc MCR-1 structure 5K4P.

A. Overall fold of multi-zinc MCR-1 (green, PDB 5K4P), with the ten zinc sites shown as grey spheres, and zinc-binding residues as sticks. B. Close-up view of the active site of a superposition of monozinc ($P2_1$) MCR-1 (grey) with 5K4P (green). The 5K4P zinc ions (green spheres) are labelled Zn1, ZnB, ZnC and ZnD. The $P2_1$ zinc ion (grey spehere) is labelled Zn1. C. Close-up view of the active site of a superposition of dizinc ($P4_12_12$) MCR-1 (cyan) with 5K4P (green). 5K4P zinc ions are coloured and labelled as B. The P41212 zinc ions (cyan speheres) are labelled Zn1 and Zn2.

Supplementary Tables

Supplementary Table S1. Data collection and refinement statistics

	Zn-edge	di-zinc native	monozinc/phosphothreonine native
**Data collection**			
Space group	$P4_12_12$	$P4_12_12$	$P2_1$
No. of datasets	4	1	1
Molecules/ASU	1	1	2
Cell dimensions			
a, b, c (Å)	48.59, 48.59, 246.79	49.07, 49.07, 244.23	52.20, 82.33, 81.74
α, β, γ (°)	90.00, 90.00, 90.00	90.00, 90.00, 90.00	90.00, 104.42, 90.00
Wavelength(s) (Å)	1.282, 1.282, 1.2825, 1.2822	0.97833	0.97831
Resolution (Å)*	28.54 – 2.50 (2.60 – 2.50)	48.85 – 1.75 (1.79 – 1.75)	50.56 – 1.55 (1.59 – 1.55)
R_{pim}	0.032 (0.155)	0.029 (0.512)	0.097 (0.670)
$CC_{1/2}$	0.999 (0.990)	0.998 (0.925)	0.993 (0.519)
$I / \sigma(I)$	25.8 (10.4)	15.1 (2.3)	9.3 (1.9)
Completeness (%)	99.9 (100.0)	100.0 (99.6)	98.0 (96.7)
Redundancy	54.2 (54.7)	15.5 (14.8)	6.7 (6.8)
**Phasing Calculations**			
FOM[1]	0.31	-	-
FOM after DM[2]	0.66	-	-
**Refinement**			
Resolution (Å)	-	48.09 – 1.75	50.56 – 1.55
No. reflections	-	31329	94874
R_{work} / R_{free}	-	16.88 / 19.8	16.51 / 18.88
No. atoms	-		
Protein	-	2450	5147
Solvent	-	183	910
Zn	-	2	2
B-factors			
Protein	-	39.9	18.7
Solvent	-	46.0	33.9
Zn	-	36.2	14.3
R.m.s. deviations			
Bond lengths (Å)	-	0.011	0.011
Bond angles (°)	-	1.163	1.227
Ramachandran (%)			
Outliers	-	0.32	0.31
Favoured	-	97.09	98.02

*Values in parentheses are for highest-resolution shell.
[1]Figure of merit
[2]Figure of merit after density modification

Supplementary Table S2. Zinc-coordination distances in Å

		***P*4$_1$2$_1$2**		***P*2$_1$**	
**residue**	**atom**	**Zn1**	**Zn2**	**Zn1**	**phosphoThr285**
E300 (symmetry)	Oε2	-	2.12	-	-
	Oε1	-	2.31	-	-
Wat1	O	-	1.91	-	-
His395	Nε2	-	1.87	-	3.17
His478	Nε2	-	2.04	-	2.90
Glu246	Oε2	2.04	-	1.97	-
Asp465	Oδ1	1.99	-	2.00	-
Thr285	Oγ1	2.10	-	2.36	-
His466	Nε2	2.10	-	2.10	-

Supplementary Table S3: Details of Bacterial Strains used in Minimal Inhibitory Concentration (MIC) Experiments

Supplementary Table 3 is provided as an Excel spreadsheet (Table_S3.xlsx).

Supplementary Table S4. Colistin MICs of MCR-1 Mutants in *E. coli* TOP10

MICs were measured in agar dilution experiments as described in Methods. Data shown are modes for two independent experiments, each performed in duplicate

**Plasmid**	**Colistin MIC (μg/ml)**
pUC19	≤0.125
pUC19 *mcr-1*	4
pUC19 *mcr-1* E246A	≤0.125
pUC19 *mcr-1* T285A	≤0.125
pUC19 *mcr-1* K333A	0.25
pUC19 *mcr-1* H395A	≤0.125
pUC19 *mcr-1* E468A	0.25
pUC19 *mcr-1* H478A	0.5

Supplementary Table S5. Primers used in this study

Primer	Sequence 5' → 3'
MCR-1^{soluble} F	AAGTTCTGTTTCAGGGCCCGACGATCTATCACGCGAAAGATGCTGTAC
MCR-1^{soluble} R	ATGGTCTAGAAAGCTTTACCGGATGAAGGCGGTGCGATC
MCR-1 pSU18 F	TCGGTCATCTCGTCCGTTTG
MCR-1 pSU18 R	TTCTCACCCAGACTTTCGCC
MCR-1 1893 F	GCTCTAGATTCAGTATGGGATTGCG
MCR-1 1893 R	CGGAATTCTCATCTCAGCAAGTAG
MCR-1 E246A F	CGTCGTCGGTGCGACGGCACGCG
MCR-1 E246A R	CGCGTGCCGTCGCACCGACGACG
MCR-1 T285A F	CGTGCGGCACATCGGCGGCGTATTCTGTG
MCR-1 T285A R	CACAGAATACGCCGCCGATGTGCCGCACG
MCR-1 K333A F	GTGGCGTGATAATAATTCGGACTCAGCAGGCGTGATGGAT
MCR-1 K333A R	ATCCATCACGCCTGCTGAGTCCGAATTATTATCACGCCAC
MCR-1 H395A F	GCTGCACCAAATGGGCAATGCCGGGCCTGCG
MCR-1 H395A R	CGCAGGCCCGGCATTGCCCATTTGGTGCAGC
MCR-1 E468A F	GTTCTCACCCAGACTTGCGCCATGATCGCTGAC
MCR-1 E468A R	GTCAGCGATCATGGCGCAAGTCTGGGTGAGAAC
MCR-1 H478A F	GGCATTTGGCATACCAGCTAGATAGACACCGTTCTCACC
MCR-1 H478A R	GGTGAGAACGGTGTCTATCTAGCTGGTATGCCAAATGCC
DsbA F	AAAAGATTTGGCTGGCGCTG
DsbA R	TGGTATCCATACCCTGCGGA

Supplementary Text

Computational Details

Starting geometries were generated from the two crystal structures reported in this work, by consideration of residues within approximately 5–10 Å of the zinc ion(s), followed by further manual selection to identify and include relevant residue side chains and loops. Residues were truncated at the alpha carbon and saturated with hydrogen atoms; hydrogen atoms were also added manually to satisfy the valencies of all other atoms in the model and, where relevant, different protonation states were also considered computationally. Geometry optimisations were performed using six Cartesian constraints on the alpha carbon atoms of residues for intermediates in which His395 and His478 were included and four where they were not present (see below). We tested a smaller numbers of Cartesian constraints, but found the deviation from the crystallographically-observed geometries too large; further molecular dynamics (MD) studies of the wider protein environment will be needed to define how flexible the active site environment is likely to be.

For both pathways, the crystallographically observed geometry was modified in line with the reaction pathways shown in Schemes S1 and S2 below, simplifying the phosphoethanolamine by replacing substituents with methyl groups to reduce conformational freedom and computational costs, removing substrate 1 to generate Zn_1 and conversely introducing substrate 2 to generate Zn_4. Residues Glu246, Thr285, Asp465 and His466 were included in all calculations for both mono- and di-Zn, and His395 and His478 were introduced to complex Zn_4 for the mono-Zn route, whereas they were present throughout the di-Zn case. For the mono-Zn pathway, the complexes modelled thus comprised of between 78 and 127 atoms, while for the di-Zn pathway, this ranged between 100 and 112 atoms.

All geometry optimisations were performed using Jaguar[5], with the B3LYP hybrid functional[6-9], which has been shown to be usefully accurate for modeling reaction mechanisms involving zinc metalloenzymes, and other transition-metal-containing enzymes[10], using loose convergence criteria (5 times larger than default criteria). Test calculations on other complexes using the more stringent default convergence criteria did not lead to significant changes in energies, bond lengths, or angles, but were much more time-consuming. A relativistic effective core potential was used for the zinc ion(s) as incorporated into Jaguar's LACV3P* basis set, and the Pople 6-31G* basis set for all other atoms, with five spherical harmonic components of the polarization functions. Transition states were located in a range of ways, including using small models of phosphate diester dissociation

and stepwise distance scans to generate suitable starting geometries within the active site, as well as using quadratic synchronous transit calculations from suitable intermediates distorted to more closely resemble likely transition states; the mono-Zn TS1 was also used to inform the di-Zn search. Transition states were confirmed by frequency calculations, but, due to the Cartesian constraints used, some included additional low imaginary frequencies. Energies including continuum-dielectric medium (CDM) solvent effects were determined from single point calculations on these geometries, incorporating the Poisson–Boltzmann finite-element model of solvation[11,12] as implemented in Jaguar. The dielectric constant was set to 4, as commonly used in the cluster model approach[13] and the probe radius to 2 Å (labelled as CDM_protein). We also performed test calculations with solvent parameters corresponding to water (ε=80.37, probe radius =1.4 Å, labelled as CDM_water) applied to fragments which could be transported out of the active site and protein environment and fully solvated in an aqueous environment, namely substrate 1 and the $[MeO]^-$ leaving group. Unless otherwise stated, vibrational frequencies were not computed for stationary points, and so the energetic data do not include a correction for zero-point energy, although we note that this would be expected to be quite small. Dispersion corrections have also not been included to date and are unlikely to affect relative energies significantly[14]; the attractive interactions within the active sites modelled would perhaps also be exaggerated in cluster models such as this, as the "balancing interactions" with the wider protein and solvent environment are not likely to be fully captured by the CDM.

DFT Calculation Results

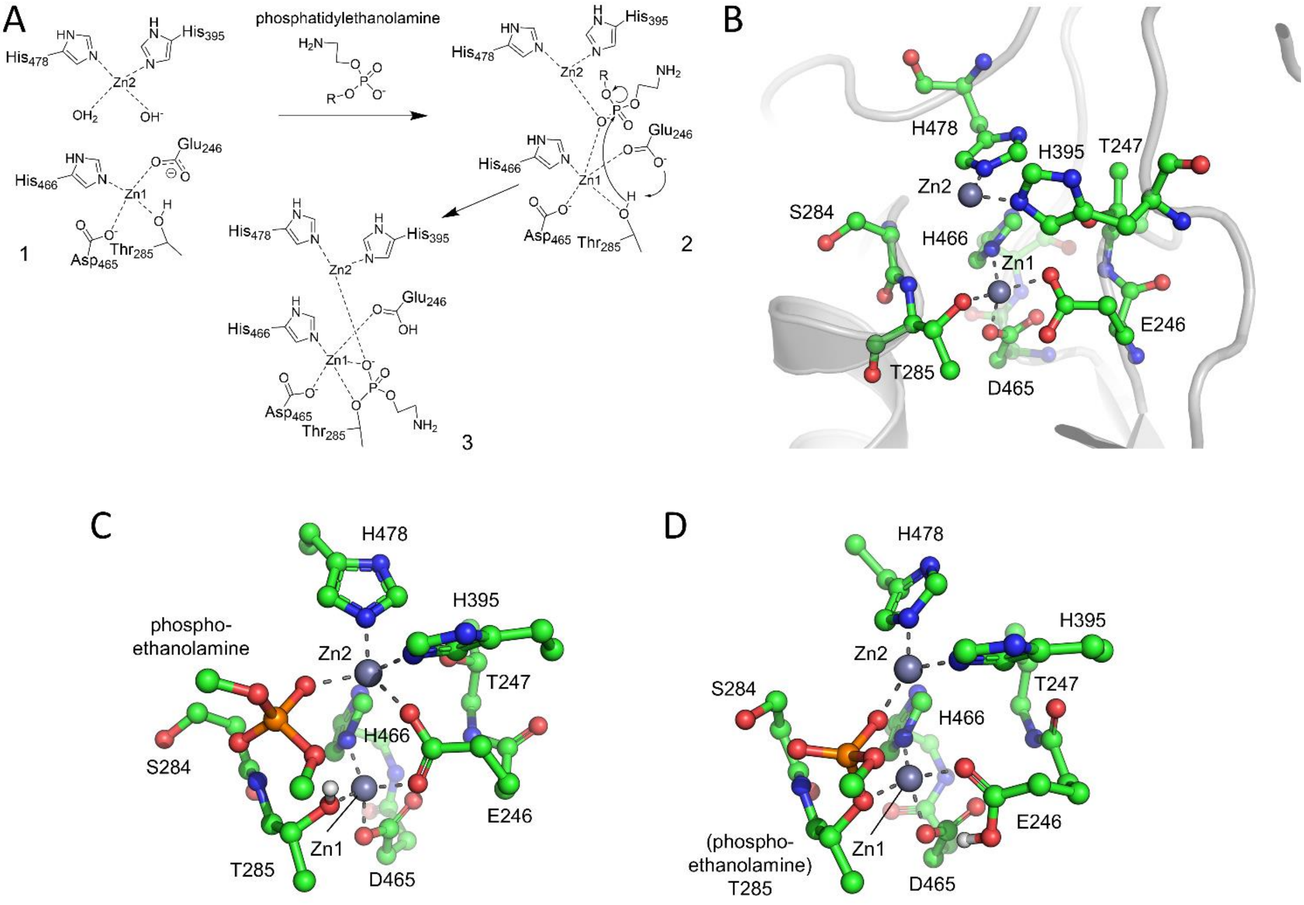

Supplementary Figure S9: **Density Functional Theory (DFT) Modelling of MCR-1 Active Site (Di-Zn(II) form).**

A. Possible mechanism for phosphoethanolamine addition (**2**) to MCR-1 Thr285 of di-Zn(II) MCR-1 (**1**) to form Thr285 adduct (**3**). B. MCR-1 di-Zn(II) active site ($P4_12_12$) crystal structure showing residues used in cluster model. Distances in Supplementary Table 2. C. DFT-optimised geometry of phosphoethanolamine non-covalently bound to MCR-1 di-Zn(II) active site (**2** in panel (A) above). Distances in Supplementary Table S9 (2Zn-2). D. DFT-optimised geometry of the MCR-1 di-Zn(II) active site in the phosphoethanolamine-bound form (**3** in panel (A) above). Distances in Supplementary Table S9 (2Zn-3E). A more complete discussion of the structural and computational features of this mechanism is in Supplementary Text below and Supplementary Tables S9 and S10.

Quantum chemical cluster approaches/active site models can provide useful insights into the mechanisms of enzyme-catalysed reactions[10,13], and were employed here to explore mechanistic postulates for zinc-dependent phospho(ethanolamine)transferases. (For a general

review of computational insights into zinc-dependent metalloenzymes, see references 15,16, and references 17-22 for related work in the field). Application of Cartesian constraints to the model (4 and 6 frozen Cα atoms in the case of mono- and di-Zn respectively, see computational details) was necessary to approximate the geometric restriction imposed by active site residues not included in the model[10]. Using a model substrate, $[P(O)O(OMe)_2]^-$, in the place of phosphatidylethanolamine and $[P(O)(OMe)OO]^{2-}$ for the phosphorylated lipid, structures (energy minima) corresponding to all intermediates in the two mechanistic postulates (main text **Figure 4A** and **Supplementary Figure S9**) were located. We also ran geometry optimizations with unconstrained and less constrained models, but observed large structural changes when compared to the crystallographically observed reference geometries.

Comparison of Calculated and Crystallographically-Observed Active Site Geometries

Supplementary Table S6a: Structural comparison of Phosphorylated mono-Zn(II) active site geometries (see later discussion of possible protonation states, Scheme S1 for labelling used).

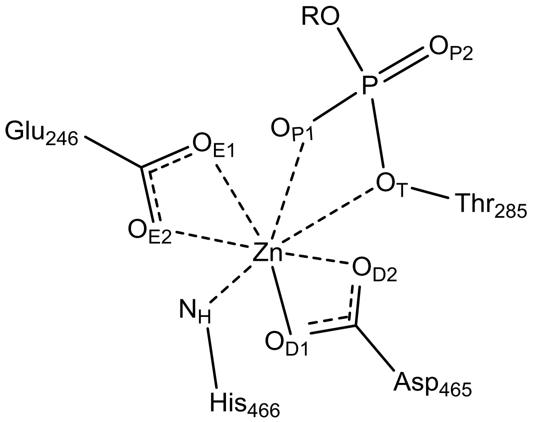

Species	O_{D1}	O_{D2}	O_T	O_{E1}	O_{E2}	N_H	O_{P1}	O_{P2}
1Zn-3D	3.698	2.395	3.456	2.128	2.139	2.110	1.958	4.503
1Zn-3E	3.236	2.011	3.094	3.282	2.103	2.060	1.946	3.094
1Zn-3B	3.283	2.050	3.261	2.242	2.160	2.222	1.999	4.368
XRD[a] (1Zn-3, *P*2$_1$ form)	3.061	1.999	2.352	2.554	1.912	2.068	2.125	3.861/4.003 [b]

[a] XRD = X-ray diffraction. Data from crystal structure.

[b] The calculations were performed for a methyl phosphate adduct ($R=CH_3$), whereas a phosphate group is observed crystallographically.

Supplementary Table S6b. Structural comparison of Di-Zn(II) active site geometry (see Scheme S2 for details of intermediates).

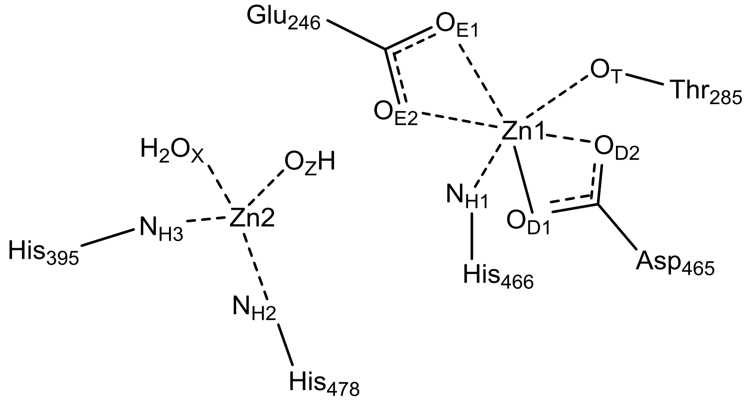

Species	O_{D1}	O_{D2}	O_T	O_{E1}-Zn1	O_{E2}-Zn1	O_{E2}-Zn2	N_{H1}	N_{H2}	N_{H3}	O_Z	O_X
2Zn-1	2.551	2.033	2.087	2.128	2.254	4.090	2.277	2.092	2.067	1.903	2.188
XRD (2Zn-1, $P4_12_12$)	2.990	1.989	2.098	2.042	2.624	4.540	2.100	2.041	1.870	N/A	1.906

Supplementary Tables S6a and S6b show comparisons between calculated geometries and the crystallographically observed active sites reported in this work, i.e. the $P2_1$ and $P4_12_12$ structures (**Figures 2C, D**). The DFT-optimized geometries are, overall, in satisfactory agreement with the crystal structures, albeit with some discrepancies, such as the Zn-O_T distances in the mono-Zn site and the Zn-O_{D1} and O_{E2}-Zn1/Zn2 distances in the di-Zn case. These differences may respectively reflect differences between the methylphosphate (modelled) and phosphate (observed) adducts, and that these more distant contacts are likely to be weak at best, and simply brought into proximity due to other, stabilizing interactions.

The data shown in **Supplementary Table S6a** also illustrate the effect of choosing different protonation states on the calculated structures, causing substantial variations in the Zn-residue distances. In terms of agreement with the crystal structure, deprotonation of both residues, Glu246 and Asp465, is perhaps most likely.

For both mono- and di-Zn structures, the calculations are thus reasonably consistent with the crystallographically-determined geometries around the active site, supporting the experimentally determined structural assignments and suggesting that residues Glu246 and Asp465 are most likely to be deprotonated.

For the second Zn ion (Zn2) in the di-Zn structure, we have explored the addition of water or hydroxide to fulfill a tetrahedral coordination environment around the metal ion. The calculated estimates of proton affinity are comparatively low (discussed below) and suggest that hydroxide may be present; this is the structure captured in **Supplementary Table S6b**.

DFT analysis of the mono-Zn(II) mechanism

Figure 4 in the main text shows the residues included in our optimisation of the mono-zinc structure, including the different protonation states explored for 1Zn_3 (Glu246 vs. Asp465 vs. doubly deprotonated). As discussed above, the doubly deprotonated state appears in best agreement with the crystal structure geometry. An extended version of **Figure 4A** is shown here as **Scheme S1**, highlighting the postulated mechanism for phosphorylation. **Supplementary Table S7** lists the key structural parameters around the Zn coordination site for all intermediates and the transition state found, while **Supplementary Table S8** shows the calculated potential energies for these pathways.

The optimised geometries (**Supplementary Table S7**) maintain a coordination geometry around the single zinc centre consistent with the crystal structure ($P2_1$ form) throughout the postulated reaction mechanism (**Scheme S1**). This shows that the (small) structural changes required by this reaction are achievable within the constraints of the active site, captured by the Cartesian constraints of the cluster model used here. The intermediates shown in **Scheme S1** thus appear to be feasible from a structural point of view. The data also suggest that the role of the metal centre is, in part, structural, holding the residues and substrate 1 in place throughout. These results therefore indicate that this single zinc reaction mechanism is structurally reasonable.

Scheme S1: Mechanistic postulate for mono-Zn mechanism. 1Zn-2 to 1Zn-3D and 1Zn-3E proceed via transition states 1Zn-TS1$_D$ and 1Zn-TS1$_E$ respectively.

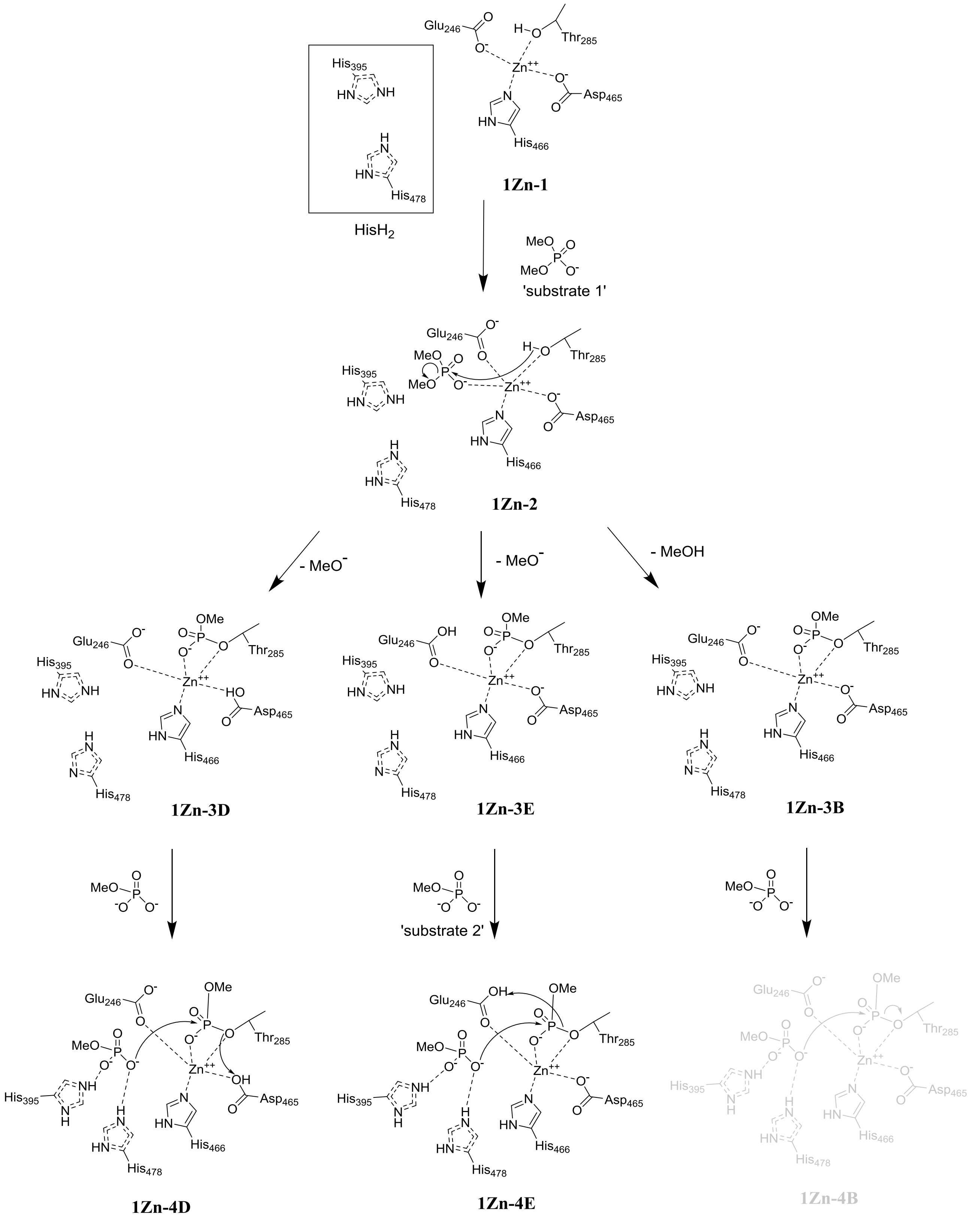

Supplementary Table S7: Key structural parameters for the mono-Zn mechanism active site (distances given in Å).

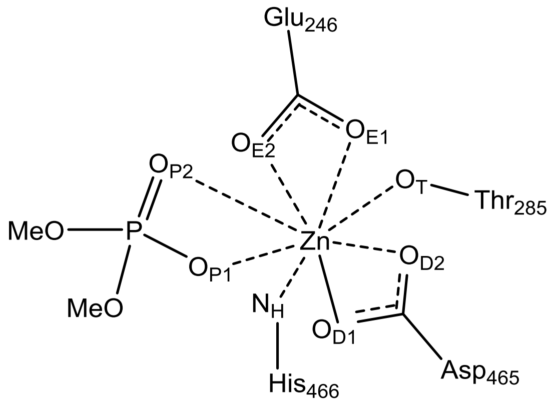

Species	O_{D1}	O_{D2}	O_T	O_{E1}	O_{E2}	N_H	O_{P1}	O_{P2}
1Zn-1	2.687	2.009	2.182	2.292	2.095	2.131	-	-
1Zn-2	3.654	2.198	2.287	2.165	2.227	2.205	2.054	3.692
1Zn-TS1	4.012	2.739	3.763	2.223	2.202	2.188	1.997	2.712
1Zn-3D	3.698	2.395	3.456	2.128	2.139	2.110	1.958	4.503
1Zn-3E	3.236	2.011	3.094	3.282	2.103	2.060	1.946	3.094
1Zn-3B	3.283	2.050	3.261	2.242	2.160	2.222	1.999	4.368
XRD (1Zn-3, $P2_1$ form)	3.061	1.999	2.352	2.554	1.912	2.068	2.125	3.861/4.003[a]
1Zn-4D	3.905	2.600	3.025	2.142	2.127	2.127	1.946	4.353
1Zn-4E	3.303	2.057	3.052	3.252	2.133	2.072	1.925	4.226

[a]The calculations were performed for a phosphate diester, whereas a phosphate group is observed crystallographically.

Supplementary Table S8: Calculated relative potential energies in kcal mol^{-1} for mono-Zn mechanism shown in Scheme S1.

No.	Species	ΔE(gp)	ΔE(sl1)[a]	ΔE(sl2)[b]
1	1Zn-1 + substrate 1 + [substrate 2.HisH_2]	0.0	0.0	0.0
2	1Zn-2 + [substrate 2.HisH_2]	-57.9	-26.1	-0.2
3	1Zn-TS1 + [substrate 2.HisH_2]	10.6	40.4	66.3
4	1Zn-3D + [substrate 2.HisH_2] + [MeO]-	50.7	42.5	33.1
5	1Zn-3E + [substrate 2.HisH_2] + [MeO]$^-$	48.2	41.5	32.0
6	1Zn-3B+ [substrate 2.HisH_2] + MeOH	-49.7	-21.6	3.1
7	1Zn-4D + [substrate 2.HisH_2][c] + [MeO]$^-$	61.2	54.8	45.3
8	1Zn-4E+ [substrate 2.HisH_2][c] + [MeO]$^-$	47.8	44.1	34.7

[a] CDM_protein

[b] CDM_protein for fragments inside protein environment and CDM_water for those external to the active site

[c] Included in cluster model

Repeated attempts to include His395 and His478 in the cluster model for the mono-Zn structure throughout the entire pathway were unsuccessful and optimisations failed to converge. As a consequence, during the **1Zn-TS1** transition state optimization, a structural change was observed in Glu246, which is unlikely to be feasible in the protein environment. More extensive calculations on larger cluster models or indeed QM/MM studies of the protein will be needed to determine if this structural change is necessary for a viable transition state to be located. The geometry of the transition state shows a concerted reaction and is consistent with the transition state for phosphate diester hydrolysis[23].

The calculated energy differences are shown in **Supplementary Table S8**. This shows that the relative energies are very sensitive to the fragments chosen to balance the reaction ($[MeO]^-$ vs. MeOH, [substrate2.$HisH_2$], see **Supplementary Table S8,** not present in early steps as discussed above), as well as the protonation state of the Glu246 and Asp465 residues (entries 4,5 vs. 6). Both the aspartate and glutamate residues are geometrically accessible to act as bases for the reactions; calculations performed also suggest that intermediates 1Zn-3D and E are almost isoenergetic. The intermediates generated by proton transfer to Glu246 and Asp465 (**1Zn-3E**, **1Zn-D**) were isoenergetic, within the limitations of the computational approach used. Comparison of **1Zn-3E** to the corresponding phosphorylated crystal structure shows closer agreement than the aspartate–protonated analogue. Optimization of the corresponding deprotonated species (**1Zn-3B**) gave the closest agreement with the crystal structure, as discussed above, and on this basis both Asp465 and Glu246 are postulated to be deprotonated in 1Zn-3. The energetic stabilization of this intermediate, however, is at least in part an artefact of balancing with MeOH, rather than an anionic fragment, as the product, so may be misleading.

Releasing the anionic model product $[MeO]^-$ into the gas-phase makes intermediates 1Zn-3 unfavourable (entries 4, 5), with TS1 lower in relative potential energy than the subsequent intermediates. This step remains energetically unfavourable, albeit to a lesser extent, when a low dielectric constant is used in the continuum model of solvation. A water model applied just to the mobile fragments, assuming that they will be transported out of the protein and fully solvated in water, (substrate 1 and $[MeO]^-$, Supplementary Table S8 column E(sl2)) can begin to address such issues.

We have also located intermediates and transition states for this cluster model without any constraints and note that in this scenario TS1 (**1Zn-TS1u**) lies within 45 kcal mol^{-1} of 1Zn-2 (**1Zn-2u**) in the gas phase. While still a substantial energy difference, this suggests that the

flexibility of the active site warrants further exploration[24] as the energies of this pathway are quite sensitive to the constraints used. In addition, the transition state (**1Zn-TS1**) lacks the stabilisation conferred by what may be one or two protonated histidine residues and as such, the energy barrier for this process is considerable. Modeling solvent explicitly has been demonstrated to lower the activation energy barrier for phosphate monoester hydrolysis[25,26], and may be worthy of exploration in future studies.

We note further that the introduction of the two protonated histidines (His395 and His478) is necessary to complete the reaction with substrate 2 (**Scheme S1**) and so complete turnover in this model system. In the intact enzyme this might be achieved by involvement either of other residues or of a second Zn centre (see discussion below).

These results suggest that a more extensive study, using QM/MM and MD simulations, will be necessary to fully test further whether a mono-Zn mechanism is indeed viable; the geometries found in this preliminary study will provide a good starting point for more extensive sampling.

DFT analysis of the Di-Zn(II) mechanism

The likely reaction pathway postulated in the presence of two zinc ions in the active site is shown as **Scheme S2**, with key structural and energetic features captured in **Supplementary Tables S9 and S10**.

Similar to the mono-Zn case, the gas-phase optimised geometries remain reasonable around both metal centres throughout the postulated reaction pathway (**Supplementary Table S9**), confirming that the mechanistic postulate can be accommodated within the active site observed crystallographically.

However, protonation of the geometrically accessible aspartate oxygen (O_{D2}) to generate **2Zn-3D** from **2Zn-2** resulted in a considerable structural change in the position of Asp465. Dissociation from the metal centre was observed, which is likely to be the cause of the approx. 8 kcal mol^{-1} drop in relative potential gas-phase energy (**Supplementary Table S10**) when compared to proton transfer to O_{E1}. A smaller structural change is required to give a geometrically sensible proton transfer intermediate. Consequently, Glu246 is more likely to be the base in the di-Zn mechanism on both energetic and geometric grounds.

Scheme S2: Mechanistic postulate for di-Zn mechanism. 2Zn-2 to 2Zn-3D and 2Zn-3E proceed via transition states 2Zn-TS1$_D$ and 2Zn-TS1$_E$ respectively.

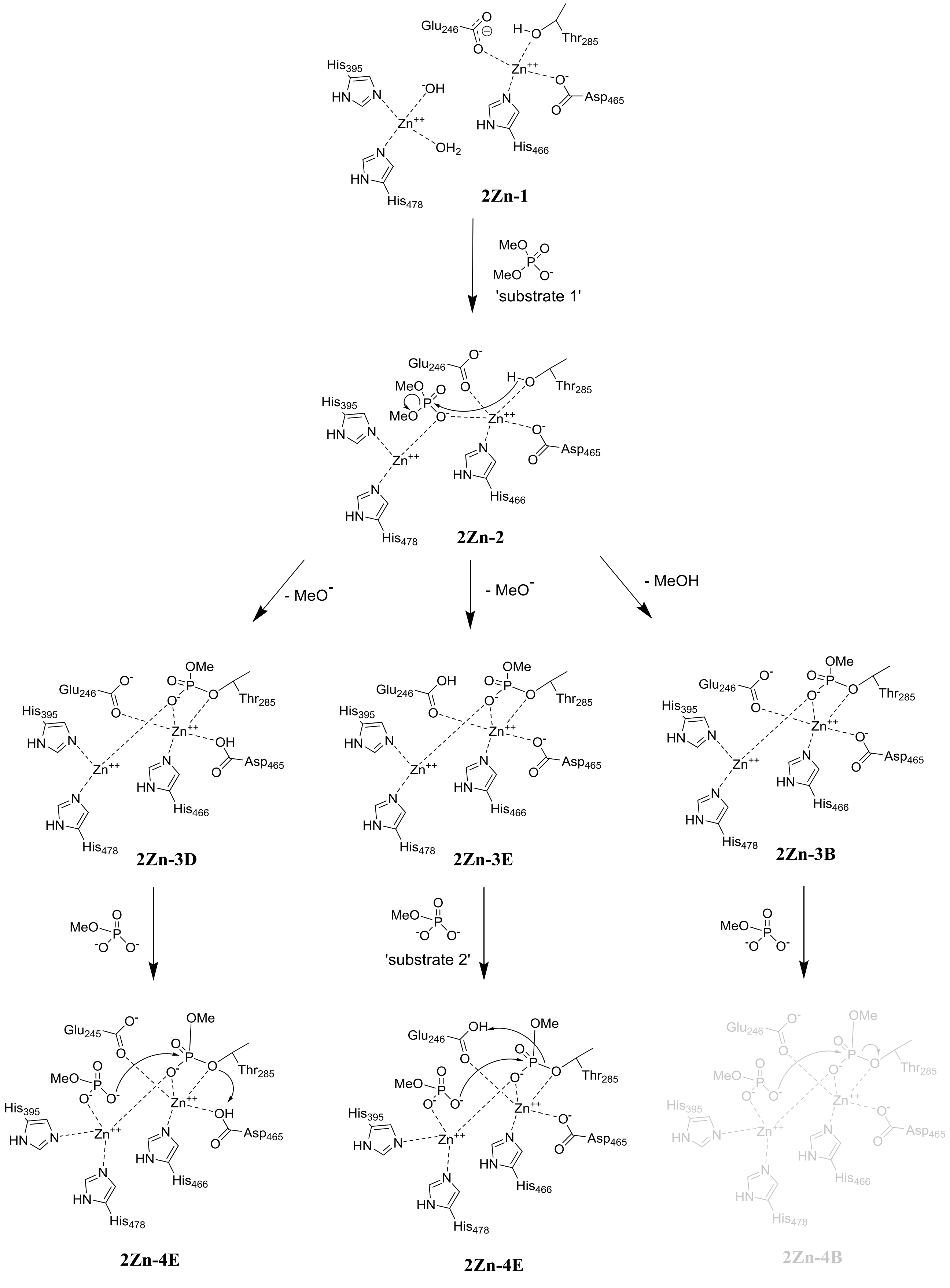

Supplementary Table S9: Important structural parameters for the di-Zn mechanism active site (distances given in Å, see Scheme S2 for species labelling).

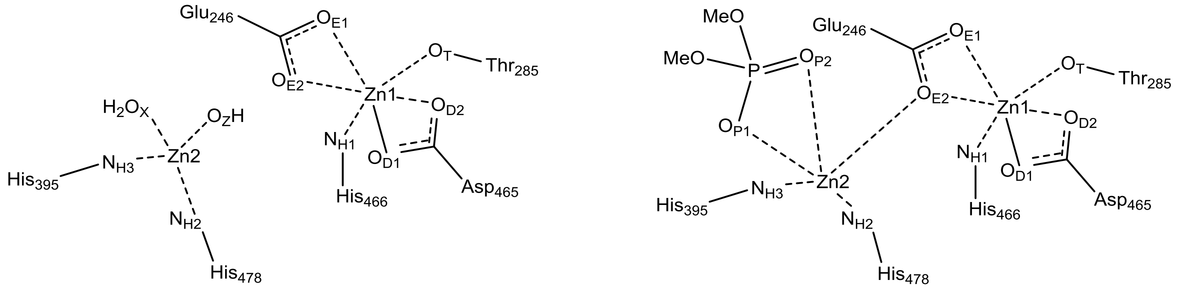

	O_{D1}	O_{D2}	O_T	O_{E1}-Zn1	O_{E2}-Zn1	O_{E2}-Zn2	N_{H1}	N_{H2}	N_{H3}
2Zn-1	2.551	2.033	2.087	2.128	2.254	4.09	2.277	2.092	2.067
XRD (2Zn-1, *P*4_12_12 form)	2.990	1.989	2.098	2.042	2.624	4.540	2.100	2.041	1.870
2Zn-2	2.723	1.995	2.077	2.012	3.153	2.035	2.084	2.095	2.098
2Zn-TS1$_E$	3.361	1.991	2.802	2.179	2.351	2.104	2.115	2.097	2.081
2Zn-3D	3.751	3.777	2.168	2.062	2.318	2.077	2.029	2.035	2.015
2Zn-3E	3.172	2.025	2.162	3.204	2.069	3.594	2.042	2.069	2.054
2Zn-3B	2.964	1.990	2.211	2.024	2.751	2.036	2.082	2.090	2.070
XRD (2Zn-3, PDB 4KAY[3])	3.143	1.990	1.951	2.305	1.795	4.547	1.997	2.465	2.224
2Zn-4D	5.563	4.661	2.178	2.079	2.224	4.406	2.123	2.166	2.167
2Zn-4E	3.494	2.020	2.343	3.348	2.206	4.301	2.102	2.122	2.202

Species	O_{P1}-Zn1	O_{P2}-Zn1	O_{P1}-Zn2	O_{P2} -Zn2	O_Z	O_X
2Zn-1	-	-	-	-	1.903	2.188
XRD (2Zn-1, *P*4_12_12 form)	-	-	-	-	-	1.906
2Zn-2	4.539	4.943	1.938	4.402	-	-
2Zn-TS1$_E$	2.109	3.882	2.569	2.513	-	-
2Zn-3D	2.270	4.064	1.993	4.433	-	-
2Zn-3E	2.559	4.229	1.923	3.994	-	-
2Zn-3B	3.400	4.376	1.937	4.617	-	-
XRD (2Zn-3, PDB 4KAY[3])	2.780	3.786	1.981	2.486		
2Zn-4D	2.046	3.930	2.324	4.135	-	-
2Zn-4E	2.102	3.847	2.213	3.901	-	-

Supplementary Table S10: Calculated relative potential energies in kcal mol^{-1} for di-Zn mechanism shown in Scheme S2.

No.	Species	ΔE(gp)	ΔE(sl1)[a]	ΔE(sl2)[b]
1	2Zn-1 + $[Zn(His)_2(H_2O)(OH^-)]^+$ +substrate 1 + substrate 2	0.0	0.0	0.0
2	2Zn-2 + H_2O + $[OH]^-$ + substrate 2	81.9	58.8	43.9
3	2Zn-TS1 + H_2O + $[OH]^-$ + substrate 2	114.1	93.3	78.5
4	2Zn-3D + H_2O + $[OH]^-$ + $[MeO]^-$ + substrate 2	310.9	172.7	122.5
5	2Zn-3E + H_2O + $[OH]^-$ + $[MeO]^-$ + substrate 2	318.2	172.0	121.7
6	2Zn-3B + H_2O + $[OH]^-$ + MeOH+ substrate 2	100.5	75.4	59.4
7	2Zn-4D+ H_2O + $[OH]^-$ + $[MeO]^-$	-459.4	55.5	-226.9
8	2Zn-4E+ H_2O + $[OH]^-$ + $[MeO]^-$	-463.5	52.3	-230.2

[a] CDM_protein
[b] CDM_protein for fragments inside protein and CDM_water for those external to the system

A transition state similar to that found for the mono-Zn pathway was located for the di-Zn mechanism (**2Zn-TS1$_E$**), indicating the reaction proceeding via attack of O_T on the phosphate diester facilitated by activation by Glu246. The early transition state shows marked differences from the mono-nuclear equivalent (**1Zn-TS1$_D$**), in O–P and O–H bond lengths, however still falls geometrically within the manifold for phosphate hydrolysis transition states[27].

The calculated relative potential energies (**Supplementary Table S10**) suffer from the same limitations as discussed for the mono-Zn mechanism above: relative energies vary considerably depending upon the balancing fragments, protonation states and solvation model used. Introducing substrate 1 to the metal cluster is unfavourable as a hydroxide anion is released into the gas phase. Gas-phase proton affinity for the coordinated hydroxide in **2Zn-1** (217 kcal mol^{-1}, calculated as the energy difference between **2Zn-1** and **2Zn-1p**, c.f. methyl amine: 214 kcal mol^{-1}) indicates that the oxygen atom observed in the crystal structure is likely to be coordinated as hydroxide.

The data shown in **Supplementary Table S10** would appear to rule out the di-Zn mechanism as too high in energy compared to that for 2Zn-1. These energies are nevertheless included here for completeness. We note that these values do not include corrections for entropy, dispersion and indeed quantum tunneling effects, and reiterate the sensitivity of the system to choices of protonation states, balancing fragments and structural constraints. Furthermore, use of model substrates will affect both the steric and electronic requirements of both reaction pathways. Within these limitations, these results indicate that the mono Zinc mechanism may be more likely, indicating that to be the catalytically active form of the enzyme.

While the relative energy differences suggest that the di-Zn pathway may be inaccessible, the "local" energy differences between 2Zn-2 and 2Zn-3 as well as 2Zn-TS1 are smaller than those observed for the corresponding mono-Zn pathway in the ΔE(sl2) case. Hence a direct quantitative comparison between the two pathways is not justified, due to the assumptions made and the intrinsic differences in the models. Nevertheless, these structures provide a good starting point for a more extensive exploration of the effects of protein environment using QM/MM and MD calculations, and so will help to define the structural requirements for the PEA transfer reaction.

Supplementary References

1. Eisenberg, D., Schwarz, E., Komaromy, M. & Wall, R. Analysis of membrane and surface protein sequences with the hydrophobic moment plot. *J Mol Biol* **179**, 125-42 (1984).
2. Baker, N.A., Sept, D., Joseph, S., Holst, M.J. & McCammon, J.A. Electrostatics of nanosystems: application to microtubules and the ribosome. *Proc Natl Acad Sci U S A* **98**, 10037-41 (2001).
3. Wanty, C. et al. The Structure of the Neisserial Lipooligosaccharide Phosphoethanolamine Transferase A (LptA) Required for Resistance to Polymyxin. *Journal of Molecular Biology* **425**, 3389-3402 (2013).
4. Wende, A. et al. Structural and Biochemical Characterization of a Halophilic Archaeal Alkaline Phosphatase. *Journal of Molecular Biology* **400**, 52-62 (2010).
5. Bochevarov, A.D. et al. Jaguar: A high-performance quantum chemistry software program with strengths in life and materials sciences. *International Journal of Quantum Chemistry* **113**, 2110-2142 (2013).
6. Becke, A.D. Density-functional exchange-energy approximation with correct asymptotic behavior. *Physical Review A* **38**, 3098-3100 (1988).
7. Lee, C., Yang, W. & Parr, R.G. Development of the Colle-Salvetti correlation-energy formula into a functional of the electron density. *Physical Review B* **37**, 785-789 (1988).
8. Slater, J.C. *The Self-consistent Field for Molecules and Solids. Vol. 4, Vol. 4,* (McGraw-Hill Book Company, New York, 1974).
9. Vosko, S.H., Wilk, L. & Nusair, M. Accurate spin-dependent electron liquid correlation energies for local spin density calculations: a critical analysis. *Canadian Journal of Physics* **58**, 1200-1211 (1980).
10. Siegbahn, P.E.M. The performance of hybrid DFT for mechanisms involving transition metal complexes in enzymes. *JBIC Journal of Biological Inorganic Chemistry* **11**, 695-701 (2006).
11. Marten, B. et al. New Model for Calculation of Solvation Free Energies: Correction of Self-Consistent Reaction Field Continuum Dielectric Theory for Short-Range Hydrogen-Bonding Effects. *The Journal of Physical Chemistry* **100**, 11775-11788 (1996).
12. Tannor, D.J. et al. Accurate First Principles Calculation of Molecular Charge Distributions and Solvation Energies from Ab Initio Quantum Mechanics and Continuum Dielectric Theory. *Journal of the American Chemical Society* **116**, 11875-11882 (1994).
13. Blomberg, M.R.A., Borowski, T., Himo, F., Liao, R.-Z. & Siegbahn, P.E.M. Quantum Chemical Studies of Mechanisms for Metalloenzymes. *Chemical Reviews* **114**, 3601-3658 (2014).
14. Lonsdale, R., Harvey, J.N. & Mulholland, A.J. Inclusion of Dispersion Effects Significantly Improves Accuracy of Calculated Reaction Barriers for Cytochrome P450 Catalyzed Reactions. *The Journal of Physical Chemistry Letters* **1**, 3232-3237 (2010).
15. Mujika, J.I., Mulholland, A.J. & Harvey, J.N. Computational Methods: Modeling of Reactivity in Zn-Containing Enzymes. in *Encyclopedia of Inorganic Chemistry* (John Wiley & Sons, Ltd, 2006).
16. van der Kamp, M.W. Zinc-Dependent Metalloenzymes – Computational Studies. in *Encyclopedia of Biophysics* (ed. Roberts, G.C.K.) 2795-2796 (Springer Berlin Heidelberg, Berlin, Heidelberg, 2013).

17. Pabis, A., Duarte, F. & Kamerlin, S.C.L. Promiscuity in the Enzymatic Catalysis of Phosphate and Sulfate Transfer. *Biochemistry* **55**, 3061-3081 (2016).
18. Park, H., Brothers, E.N. & Merz, K.M. Hybrid QM/MM and DFT Investigations of the Catalytic Mechanism and Inhibition of the Dinuclear Zinc Metallo-β-Lactamase CcrA from Bacteroides fragilis. *Journal of the American Chemical Society* **127**, 4232-4241 (2005).
19. Szeto, M.W.Y., Mujika, J.I., Zurek, J., Mulholland, A.J. & Harvey, J.N. QM/MM study on the mechanism of peptide hydrolysis by carboxypeptidase A. *Journal of Molecular Structure: THEOCHEM* **898**, 106-114 (2009).
20. Wu, R., Wang, S., Zhou, N., Cao, Z. & Zhang, Y. A Proton-Shuttle Reaction Mechanism for Histone Deacetylase 8 and the Catalytic Role of Metal Ions. *Journal of the American Chemical Society* **132**, 9471-9479 (2010).
21. Wu, S., Xu, D. & Guo, H. QM/MM Studies of Monozinc β-Lactamase CphA Suggest That the Crystal Structure of an Enzyme−Intermediate Complex Represents a Minor Pathway. *Journal of the American Chemical Society* **132**, 17986-17988 (2010).
22. Xu, D., Guo, H. & Cui, Q. Antibiotic Deactivation by a Dizinc β-Lactamase: Mechanistic Insights from QM/MM and DFT Studies. *Journal of the American Chemical Society* **129**, 10814-10822 (2007).
23. Kirby, A.J. & Nome, F. Fundamentals of Phosphate Transfer. *Accounts of Chemical Research* **48**, 1806-1814 (2015).
24. Roston, D., Demapan, D. & Cui, Q. Leaving Group Ability Observably Affects Transition State Structure in a Single Enzyme Active Site. *Journal of the American Chemical Society* **138**, 7386-7394 (2016).
25. Duarte, F., Åqvist, J., Williams, N.H. & Kamerlin, S.C.L. Resolving Apparent Conflicts between Theoretical and Experimental Models of Phosphate Monoester Hydrolysis. *Journal of the American Chemical Society* **137**, 1081-1093 (2015).
26. Duarte, F., Barrozo, A., Åqvist, J., Williams, N.H. & Kamerlin, S.C.L. The Competing Mechanisms of Phosphate Monoester Dianion Hydrolysis. *Journal of the American Chemical Society* (2016).
27. Chen, S.-L. & Liao, R.-Z. Phosphate Monoester Hydrolysis by Trinuclear Alkaline Phosphatase; DFT Study of Transition States and Reaction Mechanism. *ChemPhysChem* **15**, 2321-2330 (2014).

Supplementary Data

Coordinates for all DFT-optimised geometries

1Zn-1
E B3LYP/6-31G(d): -1991.70489728436
E B3LYP/6-31G(d) in PCM ε=4: -1991.74381299225

C	15.10180	-59.13950	10.01520
C	15.31750	-57.64970	9.68870
C	15.06490	-56.69080	10.87580
C	15.83720	-57.11460	12.10760
O	17.10290	-57.10430	12.11880
O	15.19500	-57.56110	13.12720
C	13.68620	-59.42540	10.52020
O	12.68990	-59.02340	9.91980
N	13.61580	-60.14920	11.67280
C	12.34300	-60.43800	12.32000
C	11.73090	-59.21000	13.02060
O	12.64970	-58.64540	13.95430
C	10.46740	-59.57810	13.78810
C	16.95100	-57.62600	19.06700
C	16.74540	-56.18570	19.53340
O	17.77120	-55.87610	20.46280
C	18.27410	-57.89000	18.35670
O	19.04050	-58.78410	18.69030
N	18.53950	-57.07440	17.28430
C	19.62100	-57.44000	16.38500
C	19.59950	-56.59570	15.11580
C	20.63210	-57.06630	14.09830
O	18.25310	-56.67690	14.55690
H	18.23980	-56.31260	13.64900
C	16.99670	-63.49390	13.69950
C	17.97330	-62.30570	13.61780
C	17.32410	-60.92390	13.58080
O	18.10040	-59.92990	13.84130
O	16.10600	-60.77870	13.31690
C	16.33270	-63.66370	15.07150
O	16.75110	-64.45160	15.91370
N	15.22400	-62.88310	15.27600
C	14.50000	-62.89700	16.53300
C	15.07890	-61.95160	17.62050
C	15.24850	-60.57070	17.08660
N	14.19450	-59.72040	16.77080
C	14.67470	-58.67670	16.06020
N	15.98820	-58.78690	15.91370
C	16.35030	-59.96260	16.55310
Zn	16.91350	-58.32300	14.05130
H	15.85300	-59.49320	10.73120
H	15.24360	-59.72020	9.09460
H	14.63800	-57.36480	8.87900

H	16.34390	-57.51400	9.33040
H	13.99870	-56.66030	11.11510
H	15.38350	-55.68110	10.59120
H	14.47220	-60.36400	12.17470
H	12.51730	-61.23230	13.05530
H	11.64020	-60.81090	11.56640
H	11.48390	-58.47310	12.24360
H	10.04500	-58.69160	14.27240
H	9.70980	-60.00150	13.11950
H	10.69160	-60.31910	14.56670
H	16.92020	-58.29420	19.93160
H	16.12770	-57.89720	18.39690
H	15.74610	-56.09980	19.99180
H	16.76450	-55.49680	18.67210
H	17.75920	-56.58580	16.86430
H	19.55850	-58.50470	16.11880
H	20.58350	-57.29870	16.88940
H	19.76510	-55.54120	15.37350
H	20.60580	-56.44490	13.19530
H	21.64140	-56.99770	14.51940
H	20.43990	-58.10530	13.81090
H	17.54950	-64.41910	13.51600
H	16.23410	-63.38600	12.92060
H	18.57450	-62.39320	12.70240
H	18.68900	-62.32590	14.44690
H	15.07090	-62.12910	14.61250
H	14.51810	-63.92090	16.91540
H	13.45570	-62.63170	16.32630
H	16.05780	-62.33110	17.92790
H	14.42530	-61.97750	18.50300
H	14.04530	-57.90490	15.64740
H	17.37590	-60.29700	16.58230
H	13.42190	-58.30380	13.45970
H	17.66990	-54.94950	20.72960
H	13.21150	-59.88690	16.93650

1Zn-2
E B3LYP/6-31G(d): -2713.98457163202
E B3LYP/6-31G(d) in PCM ε=4: -2714.05784663468

C	14.80280	-58.66870	10.00690
C	14.66590	-57.14440	9.81880
C	14.13730	-56.41020	11.06860
C	14.96580	-56.72010	12.30480
O	16.20180	-56.43720	12.31710
O	14.43090	-57.29550	13.30770
C	13.47730	-59.28830	10.44820
O	12.46000	-59.19210	9.75390
N	13.50260	-59.90370	11.65550
C	12.30310	-60.44230	12.26540

C	11.45620	-59.37550	12.98860
O	12.16260	-58.78800	14.06800
C	10.17890	-59.98330	13.56170
C	16.95100	-57.62600	19.06700
C	17.14900	-57.40660	20.57610
O	17.75460	-58.51660	21.21030
C	18.28010	-57.81730	18.36400
O	19.20840	-58.46660	18.87900
N	18.40460	-57.24820	17.14670
C	19.62100	-57.44000	16.38500
C	19.61080	-56.67510	15.06170
C	20.98870	-56.68180	14.39790
O	18.63140	-57.19210	14.15860
P	15.41340	-54.84200	16.06750
O	14.04100	-55.38270	16.25640
O	16.54620	-55.74520	15.52500
O	15.93130	-54.26530	17.51970
C	17.33260	-63.02380	13.97220
C	17.90750	-61.59670	13.86500
C	16.88310	-60.48540	13.55160
O	17.33510	-59.28990	13.63570
O	15.72130	-60.81070	13.22790
C	16.57650	-63.31240	15.27590
O	17.08200	-63.94860	16.20050
N	15.29840	-62.83020	15.31960
C	14.50000	-62.89700	16.53300
C	14.89320	-61.83760	17.60200
C	14.95760	-60.44570	17.05970
N	13.88060	-59.57520	16.93140
C	14.28910	-58.47030	16.25980
N	15.56920	-58.57770	15.94260
C	15.99130	-59.79110	16.44400
Zn	16.40380	-57.40410	14.26660
H	15.60130	-58.89160	10.72380
H	15.08350	-59.12090	9.04710
H	13.97530	-56.95340	8.98920
H	15.64470	-56.73280	9.54740
H	13.09500	-56.68120	11.25720
H	14.17700	-55.32780	10.89200
H	14.36690	-59.96730	12.19590
H	12.60870	-61.20620	12.99000
H	11.69620	-60.91820	11.48530
H	11.18410	-58.61240	12.24180
H	9.59270	-59.21200	14.07250
H	9.56140	-60.42910	12.77320
H	10.42330	-60.76340	14.29470
H	16.36370	-58.54070	18.91860
H	16.38830	-56.79430	18.63000
H	16.17350	-57.25090	21.05180
H	17.73530	-56.48230	20.73350
H	17.62950	-56.72670	16.72080

H	19.78340	-58.51110	16.18730
H	20.48000	-57.11750	16.98910
H	19.29120	-55.64400	15.23980
H	20.93450	-56.17820	13.42810
H	21.73120	-56.16850	15.02020
H	21.33830	-57.70930	14.23180
H	18.15270	-63.74730	13.93810
H	16.67470	-63.19780	13.11390
H	18.65160	-61.57340	13.05650
H	18.45500	-61.32680	14.77650
H	15.06080	-62.14620	14.60150
H	14.62130	-63.89390	16.96700
H	13.44970	-62.77130	16.24590
H	15.88330	-62.10660	17.98310
H	14.19420	-61.90860	18.44730
H	13.67160	-57.60650	16.05640
H	17.01270	-60.12220	16.33740
H	12.90600	-58.25350	13.71150
H	18.50260	-58.74750	20.62000
H	18.75550	-58.14840	13.97500
O	15.41430	-53.48630	15.14730
C	17.15660	-53.55070	17.60020
H	17.30180	-53.27720	18.65030
H	18.00540	-54.16370	17.27320
H	17.11950	-52.63400	16.99730
H	12.92740	-59.74700	17.21550
C	16.30820	-53.34280	14.04190
H	16.02540	-52.41320	13.53690
H	17.35100	-53.26150	14.37500
H	16.23420	-54.17940	13.34170

1Zn-3D

E B3LYP/6-31G(d): -2598.74245967702

E B3LYP/6-31G(d) in PCM ε=4: -2598.78172349237

C	14.42300	-58.27930	9.94990
C	13.94330	-56.81710	9.77820
C	13.07740	-56.27930	10.93280
C	13.66150	-56.47960	12.31810
O	14.91370	-56.52570	12.49450
O	12.87580	-56.62260	13.32410
C	13.28420	-59.18810	10.40220
O	12.35390	-59.49530	9.66130
N	13.33080	-59.54690	11.72560
C	12.34300	-60.43800	12.32000
C	10.93170	-59.82080	12.41400
O	10.87550	-58.68150	13.27310
C	9.93190	-60.83270	12.96460
C	16.95100	-57.62600	19.06700
C	16.76340	-58.68080	20.17690

O	16.28590	-59.91870	19.68670
C	17.93170	-58.09490	18.01430
O	17.92420	-59.27260	17.60430
N	18.79970	-57.17140	17.54920
C	19.62100	-57.44000	16.38500
C	19.39050	-56.41330	15.26980
C	20.14760	-56.74630	13.99180
O	17.97150	-56.38710	14.97270
C	17.69530	-61.97710	14.64040
C	17.78480	-60.43960	14.58830
C	16.54730	-59.77080	14.02450
O	16.45640	-58.42820	14.14500
O	15.63560	-60.35970	13.47260
C	16.81700	-62.48640	15.79390
O	17.28820	-62.82640	16.87270
N	15.47760	-62.53570	15.52100
C	14.50000	-62.89700	16.53300
C	14.18320	-61.76720	17.55760
C	13.81220	-60.47450	16.90720
N	12.56770	-60.20590	16.35190
C	12.61790	-59.02360	15.69560
N	13.83840	-58.51080	15.78780
C	14.58030	-59.39720	16.55530
Zn	14.59310	-56.97900	14.54830
H	15.25260	-58.30580	10.66210
H	14.78610	-58.64920	8.98500
H	13.34920	-56.74400	8.86050
H	14.82570	-56.18110	9.65050
H	12.08210	-56.73480	10.91690
H	12.91840	-55.20020	10.79960
H	14.22150	-59.49470	12.20800
H	12.70200	-60.69960	13.31970
H	12.26980	-61.36190	11.72820
H	10.63060	-59.53560	11.39920
H	8.94500	-60.36960	13.05970
H	9.84680	-61.70180	12.30360
H	10.23700	-61.18490	13.95960
H	15.98720	-57.45670	18.56870
H	17.26290	-56.66790	19.49710
H	16.02520	-58.31360	20.89770
H	17.71570	-58.80790	20.71960
H	18.57430	-56.18930	17.72750
H	19.39710	-58.45030	16.03920
H	20.68610	-57.42210	16.65700
H	19.68040	-55.42010	15.62550
H	19.94230	-55.99240	13.22680
H	21.22690	-56.76930	14.17970
H	19.85020	-57.72570	13.59760
H	18.69630	-62.37450	14.82440
H	17.34060	-62.36220	13.67830
H	18.62780	-60.14020	13.94760

H	17.98490	-60.03980	15.59010
H	15.16200	-62.09000	14.66830
H	14.88260	-63.76020	17.08470
H	13.58610	-63.20590	16.01310
H	15.06000	-61.59560	18.18580
H	13.38380	-62.11820	18.22460
H	11.79290	-58.63640	15.11130
H	15.61470	-59.22180	16.80660
H	11.52830	-58.00550	12.99840
H	16.90120	-60.16170	18.96680
H	11.74100	-60.78160	16.43410
P	17.05060	-55.16360	15.63030
O	17.18000	-54.08710	14.42610
O	15.63550	-55.75090	15.66100
O	17.65220	-54.66300	16.90480
H	17.23340	-57.92510	14.50640
C	16.74530	-52.74040	14.67270
H	17.04260	-52.15500	13.80040
H	15.65550	-52.70060	14.78130
H	17.22140	-52.34000	15.57280

1Zn-3E

E B3LYP/6-31G(d): -2598.74641580124

E B3LYP/6-31G(d) in PCM ε=4: -2598.78342224583

C	15.57220	-59.62580	10.43670
C	15.87420	-58.25700	9.80110
C	15.34130	-57.04300	10.60100
C	15.80050	-57.00380	12.03660
O	17.10310	-56.85870	12.18430
O	14.99950	-57.10460	12.98160
C	14.07280	-59.84950	10.65400
O	13.26320	-59.75060	9.73260
N	13.71840	-60.14070	11.93700
C	12.34300	-60.43800	12.32000
C	11.44650	-59.18480	12.30720
O	12.00010	-58.14850	13.12540
C	10.04750	-59.47970	12.83050
C	16.95100	-57.62600	19.06700
C	16.74970	-58.59630	20.24680
O	16.39250	-59.90280	19.83110
C	18.02180	-58.10900	18.10690
O	18.17940	-59.32180	17.86870
N	18.75500	-57.14900	17.51330
C	19.62100	-57.44000	16.38500
C	19.63040	-56.28940	15.35910
C	20.49030	-56.62500	14.14830
O	18.28480	-56.01580	14.86850
C	17.81210	-62.29690	14.74050
C	18.06360	-60.77990	14.83310

C	16.90540	-59.87590	14.40250
O	17.04620	-58.62900	14.70640
O	15.91760	-60.34390	13.79610
C	16.86610	-62.83930	15.81960
O	17.25000	-63.52560	16.75990
N	15.55280	-62.50050	15.62990
C	14.50000	-62.89700	16.53300
C	14.17390	-61.88410	17.66960
C	13.91960	-60.52070	17.12600
N	12.73390	-60.08660	16.54280
C	12.94460	-58.88890	15.94820
N	14.21160	-58.52840	16.11310
C	14.81770	-59.53140	16.85250
Zn	15.50660	-57.37070	15.00540
H	16.12580	-59.75710	11.37240
H	15.91800	-60.40670	9.74820
H	15.40630	-58.21430	8.81240
H	16.95590	-58.15500	9.66220
H	14.24880	-57.04230	10.60500
H	15.68110	-56.11930	10.11690
H	14.45100	-60.19440	12.64350
H	12.36870	-60.86510	13.32880
H	11.92430	-61.18550	11.63500
H	11.38230	-58.83530	11.26850
H	9.44140	-58.56840	12.82860
H	9.54850	-60.22850	12.20580
H	10.08530	-59.86070	13.85970
H	16.00860	-57.54530	18.50970
H	17.18370	-56.61940	19.43420
H	15.93850	-58.22690	20.88350
H	17.66540	-58.61270	20.86200
H	18.51200	-56.16830	17.67630
H	19.27230	-58.35720	15.91100
H	20.65710	-57.59710	16.71770
H	19.99730	-55.37710	15.84110
H	20.49230	-55.79090	13.44020
H	21.52370	-56.82260	14.45400
H	20.10670	-57.51570	13.63770
H	18.75970	-62.82460	14.87540
H	17.41870	-62.54270	13.74700
H	18.91750	-60.51770	14.19170
H	18.34880	-60.49150	15.85050
H	15.35410	-61.86000	14.86780
H	14.79830	-63.84270	16.99480
H	13.59280	-63.08230	15.94310
H	15.02040	-61.82070	18.35980
H	13.31710	-62.26140	18.24390
H	12.20900	-58.36450	15.35610
H	15.86200	-59.50230	17.11980
H	12.90470	-57.97090	12.81100
H	17.08460	-60.15100	19.18450

H	11.84530	-60.56700	16.57690
P	17.33150	-54.91980	15.63920
O	17.37480	-53.71890	14.54920
O	15.92730	-55.56130	15.58490
O	17.88490	-54.50870	16.96770
H	17.40210	-56.84760	13.12670
C	16.83510	-52.44400	14.92950
H	17.09760	-51.74510	14.13250
H	15.74420	-52.49850	15.02160
H	17.26930	-52.10900	15.87620

1Zn-3B
E B3LYP/6-31G(d): -2598.25940723173
E B3LYP/6-31G(d) in PCM ε=4: -2598.33141578330

C	15.16530	-58.96640	10.23020
C	15.00500	-57.48470	9.82290
C	14.20350	-56.63370	10.82830
C	14.74990	-56.72840	12.24720
O	15.97590	-56.57010	12.46270
O	13.94540	-57.01060	13.21130
C	13.81250	-59.60470	10.53600
O	12.95250	-59.76170	9.66380
N	13.61140	-59.91470	11.84390
C	12.34300	-60.43800	12.32000
C	11.21430	-59.38650	12.32410
O	11.47360	-58.30400	13.20820
C	9.88950	-60.00660	12.75870
C	16.95100	-57.62600	19.06700
C	16.79130	-58.53520	20.29790
O	16.49540	-59.87760	19.95710
C	18.04440	-58.12460	18.13410
O	18.21510	-59.34330	17.92530
N	18.77880	-57.17010	17.54230
C	19.62100	-57.44000	16.38500
C	19.77750	-56.16500	15.53940
C	20.72750	-56.38440	14.36720
O	18.51070	-55.77740	14.97740
C	17.67350	-62.36020	14.48760
C	17.95640	-60.85430	14.66450
C	16.82640	-59.89480	14.24950
O	17.03330	-58.67150	14.55070
O	15.81060	-60.34660	13.66390
C	16.77680	-62.96990	15.57180
O	17.19380	-63.77590	16.40140
N	15.47520	-62.55220	15.52070
C	14.50000	-62.89700	16.53300
C	14.28030	-61.78690	17.60410
C	14.06750	-60.43520	17.00290
N	12.89390	-60.00110	16.39740

C	13.13390	-58.80850	15.78960
N	14.39460	-58.45090	15.96460
C	14.97520	-59.45010	16.72430
Zn	15.60440	-57.20160	14.58110
H	15.83880	-59.04610	11.08960
H	15.61710	-59.51570	9.39540
H	14.49760	-57.43750	8.85190
H	16.00340	-57.04970	9.70340
H	13.14920	-56.92580	10.82840
H	14.24990	-55.58090	10.51930
H	14.38970	-59.86270	12.50410
H	12.49980	-60.81090	13.33820
H	12.03230	-61.27950	11.68550
H	11.11430	-59.01890	11.29290
H	9.10560	-59.24220	12.77690
H	9.58310	-60.80600	12.07440
H	9.97220	-60.42700	13.77010
H	16.01290	-57.61940	18.49870
H	17.14190	-56.59090	19.37270
H	15.96590	-58.16750	20.91970
H	17.70950	-58.47520	20.91110
H	18.50070	-56.19030	17.68580
H	19.15730	-58.22250	15.77860
H	20.61660	-57.78350	16.70160
H	20.14500	-55.35120	16.17750
H	20.81690	-55.46510	13.78040
H	21.72390	-56.67460	14.72200
H	20.34140	-57.17240	13.71180
H	18.61410	-62.91660	14.52920
H	17.21720	-62.52100	13.50340
H	18.82830	-60.58210	14.05330
H	18.23350	-60.61960	15.69890
H	15.27010	-61.81040	14.85090
H	14.85030	-63.80780	17.02550
H	13.54360	-63.12100	16.03970
H	15.16060	-61.72460	18.25130
H	13.43690	-62.08150	18.24610
H	12.39300	-58.29990	15.19010
H	16.02070	-59.43380	16.98700
H	12.34700	-57.89390	13.00570
H	17.13870	-60.09080	19.24730
H	12.00450	-60.47880	16.40130
P	17.42820	-54.86740	15.78550
O	17.44910	-53.53470	14.83910
O	16.06860	-55.55830	15.62030
O	17.89540	-54.54280	17.18090
C	16.52730	-52.49920	15.17190
H	16.70160	-51.68000	14.46800
H	15.49260	-52.84830	15.07140
H	16.69200	-52.14070	16.19570

1Zn-4D
E B3LYP/6-31G(d): -3891.89195723627
E B3LYP/6-31G(d) in PCM ε=4: -3891.95422950510

C	15.63340	-59.89360	10.44490
C	15.91630	-58.52530	9.78450
C	15.08540	-57.36290	10.35560
C	15.19090	-57.16890	11.85600
O	16.30730	-57.22520	12.44070
O	14.12820	-56.95820	12.55150
C	14.13830	-60.14340	10.64430
O	13.35630	-60.25940	9.70240
N	13.72900	-60.16840	11.95090
C	12.34300	-60.43800	12.32000
C	11.36370	-59.29350	11.98720
O	11.61210	-58.11900	12.75510
C	9.92470	-59.71500	12.26890
C	16.95110	-57.62600	19.06700
C	16.72140	-58.67220	20.17480
O	16.23920	-59.90800	19.67760
C	17.94700	-58.10970	18.03140
O	17.95700	-59.29510	17.63180
N	18.79680	-57.18460	17.55100
C	19.62110	-57.44000	16.38500
C	19.48990	-56.30650	15.35460
C	20.29360	-56.56900	14.08770
O	18.10240	-56.19030	14.99770
P	17.14810	-55.05170	15.71760
O	17.65480	-54.69460	17.08390
O	15.75140	-55.68910	15.58760
O	17.29080	-53.83710	14.66570
C	12.26000	-55.60700	9.49800
C	12.99140	-54.28150	9.20850
C	12.60450	-53.16580	10.13000
N	11.37910	-52.51630	10.08150
C	11.34820	-51.59080	11.08110
N	12.47370	-51.60350	11.76250
C	13.25970	-52.57530	11.18200
C	18.00770	-62.57650	15.07400
C	18.26670	-61.06510	14.95010
C	17.12180	-60.30800	14.29990
O	17.08170	-58.97740	14.50750
O	16.25450	-60.81460	13.61130
C	16.93010	-62.88370	16.12170
O	17.20010	-63.22430	17.26710
N	15.64840	-62.73030	15.67080
C	14.50000	-62.89700	16.53300
C	14.15890	-61.68830	17.45510
C	13.94850	-60.41050	16.71040
N	12.78020	-60.06020	16.04290

C	12.99600	-58.92110	15.33840
N	14.24880	-58.52020	15.50810
C	14.83630	-59.42500	16.37870
C	10.31300	-56.63700	19.27900
C	10.68970	-56.53770	17.78760
C	10.24140	-55.26960	17.12760
N	8.90490	-54.96090	16.90530
C	8.84780	-53.73770	16.30860
N	10.05550	-53.24300	16.14370
C	10.93200	-54.18330	16.64820
Zn	15.29550	-57.09670	14.32430
H	16.16230	-59.95870	11.39830
H	16.01870	-60.68850	9.79560
H	15.68900	-58.58830	8.71430
H	16.98490	-58.30540	9.88210
H	14.03550	-57.50810	10.10390
H	15.40720	-56.42630	9.88030
H	14.44510	-60.20270	12.66720
H	12.31870	-60.64930	13.39210
H	12.00360	-61.33870	11.78950
H	11.46830	-59.07670	10.91660
H	9.24460	-58.88700	12.04680
H	9.63740	-60.57700	11.65720
H	9.79400	-59.98150	13.32640
H	16.00270	-57.44090	18.54490
H	17.26910	-56.66990	19.49560
H	15.96870	-58.29370	20.87420
H	17.65630	-58.81080	20.74420
H	18.54660	-56.19960	17.71510
H	19.35330	-58.41120	15.95960
H	20.67800	-57.53130	16.67540
H	19.82220	-55.37210	15.82130
H	20.17600	-55.73150	13.39470
H	21.35870	-56.68730	14.31850
H	19.93960	-57.47520	13.58280
H	12.58350	-56.38850	8.80260
H	12.44300	-55.94020	10.52300
H	11.17510	-55.49100	9.37750
H	12.81990	-53.98710	8.16280
H	14.07290	-54.42780	9.30920
H	10.50020	-50.94860	11.27260
H	12.96150	-50.72750	13.15200
H	14.24710	-52.79760	11.56140
H	18.92540	-63.06350	15.41180
H	17.73260	-62.98810	14.09690
H	19.15830	-60.88940	14.32970
H	18.46760	-60.63010	15.93580
H	15.52060	-62.32730	14.75170
H	14.68310	-63.76290	17.17660
H	13.63940	-63.13120	15.89530
H	14.96910	-61.53960	18.17400

H	13.26780	-61.95440	18.04070
H	12.26720	-58.46840	14.67460
H	15.85480	-59.32690	16.71700
H	9.22600	-56.60480	19.42530
H	10.74890	-55.80640	19.84380
H	10.67990	-57.57520	19.71090
H	10.27850	-57.40670	17.25110
H	11.77840	-56.60010	17.68030
H	7.91980	-53.26180	16.02340
H	11.99830	-54.00080	16.61430
H	10.63630	-52.70160	9.42280
H	8.11470	-55.55170	17.11990
H	10.70730	-51.81780	15.45340
P	12.69130	-50.90950	15.31550
O	12.81570	-49.68460	16.37050
O	13.33150	-52.17300	15.78960
O	13.30400	-50.27530	13.98560
O	11.12160	-50.98160	15.06720
C	14.11620	-49.35230	16.86160
H	13.97530	-48.60590	17.64750
H	14.61330	-50.23500	17.27850
H	14.73460	-48.92450	16.06330
H	12.48400	-57.73940	12.51010
H	11.90020	-60.55520	16.08710
H	16.87960	-60.17320	18.98950
H	17.79280	-58.65830	15.09070
C	16.70590	-52.56420	15.03620
H	16.86850	-51.90010	14.18510
H	15.63180	-52.65990	15.22610
H	17.21180	-52.16790	15.92190

1Zn-4E

E B3LYP/6-31G(d): -3891.91340141870

E B3LYP/6-31G(d) in PCM ε=4: -3891.97116022729

C	15.51550	-60.11170	10.20600
C	15.89770	-58.78650	9.51870
C	15.52550	-57.51640	10.31880
C	15.97860	-57.55070	11.75590
O	17.24400	-57.85150	11.91630
O	15.19990	-57.32360	12.70470
C	14.02260	-60.18820	10.52840
O	13.16570	-60.11000	9.64930
N	13.72020	-60.32200	11.85270
C	12.34300	-60.43800	12.32000
C	11.54820	-59.12650	12.16820
O	12.17180	-58.04700	12.87200
C	10.13210	-59.26070	12.71490
C	16.95110	-57.62600	19.06700
C	16.73470	-58.61000	20.23150

O	16.32980	-59.89630	19.79360
C	18.00120	-58.12210	18.09040
O	18.12130	-59.33600	17.81940
N	18.75320	-57.17020	17.51790
C	19.62110	-57.44000	16.38500
C	19.70030	-56.20770	15.46580
C	20.64130	-56.43940	14.29130
O	18.39330	-55.90680	14.91550
P	17.34060	-54.93040	15.70840
O	17.83260	-54.55440	17.07360
O	15.99550	-55.69870	15.60940
O	17.27420	-53.68430	14.68490
C	12.26000	-55.60700	9.49800
C	12.57580	-54.14380	9.13250
C	12.31610	-53.16960	10.24110
N	11.07780	-52.97900	10.83890
C	11.22010	-52.05400	11.82880
N	12.46720	-51.63580	11.89900
C	13.15430	-52.32270	10.92270
C	17.89640	-62.41920	14.86180
C	18.15570	-60.90120	14.86790
C	17.06580	-60.02580	14.25870
O	17.26850	-58.73780	14.39880
O	16.08540	-60.50570	13.66440
C	16.89140	-62.88650	15.92250
O	17.22490	-63.49630	16.93170
N	15.58820	-62.57570	15.63820
C	14.50000	-62.89700	16.53300
C	14.17740	-61.81500	17.60590
C	13.99800	-60.47540	16.98000
N	12.84940	-60.03220	16.33450
C	13.13050	-58.88510	15.66780
N	14.40660	-58.57220	15.84530
C	14.94750	-59.54500	16.66740
C	10.31300	-56.63700	19.27900
C	10.39910	-56.50780	17.74610
C	9.91600	-55.19340	17.21290
N	8.58870	-54.78680	17.27200
C	8.50120	-53.54700	16.71390
N	9.68200	-53.13690	16.30210
C	10.57100	-54.14890	16.60860
Zn	15.74120	-57.41050	14.76630
H	16.12150	-60.26890	11.10440
H	15.73860	-60.93410	9.51610
H	15.38530	-58.72400	8.55300
H	16.97500	-58.78940	9.32310
H	14.44760	-57.34620	10.31060
H	15.99770	-56.64410	9.84720
H	14.47790	-60.36360	12.53060
H	12.37620	-60.73380	13.37400
H	11.83230	-61.22690	11.75360

H	11.50500	-58.89120	11.09760
H	9.60000	-58.30860	12.62350
H	9.57250	-60.02500	12.16470
H	10.14330	-59.54000	13.77690
H	16.00940	-57.51540	18.51280
H	17.21000	-56.63080	19.44570
H	15.94470	-58.22830	20.88760
H	17.65790	-58.66920	20.83300
H	18.50840	-56.18830	17.68820
H	19.22610	-58.29020	15.82660
H	20.63610	-57.69060	16.72510
H	20.03430	-55.34220	16.04930
H	20.67220	-55.55100	13.65400
H	21.65710	-56.65020	14.64450
H	20.30110	-57.28730	13.68630
H	12.61550	-56.29180	8.72020
H	12.71560	-55.88400	10.45450
H	11.17950	-55.76890	9.60140
H	12.01160	-53.86620	8.22990
H	13.63450	-54.05100	8.86500
H	10.42010	-51.73610	12.48270
H	12.90790	-50.65480	13.25040
H	14.21330	-52.17090	10.76770
H	18.83210	-62.93810	15.08440
H	17.55910	-62.73090	13.86600
H	19.07180	-60.68540	14.29960
H	18.34620	-60.53380	15.88350
H	15.42630	-61.98090	14.83210
H	14.75660	-63.82600	17.05040
H	13.60470	-63.08510	15.92650
H	15.00080	-61.74880	18.32300
H	13.28680	-62.12910	18.16680
H	12.43730	-58.36150	15.02370
H	15.98270	-59.53340	16.97500
H	9.27870	-56.55380	19.63540
H	10.89840	-55.85100	19.76670
H	10.70080	-57.60740	19.60900
H	9.83800	-57.33260	17.27950
H	11.44150	-56.62810	17.43000
H	7.57270	-52.99860	16.63450
H	11.62230	-54.03280	16.37840
H	10.22130	-53.45590	10.59670
H	7.82280	-55.31140	17.66940
H	10.33920	-51.75390	15.51220
P	12.36600	-50.93890	15.33690
O	12.43120	-49.84950	16.53050
O	13.00580	-52.24480	15.67330
O	12.98940	-50.13520	14.11060
O	10.80220	-51.00710	15.01610
C	13.69340	-49.62100	17.16690
H	13.49820	-48.97620	18.02720

H	14.13610	-50.56390	17.50400
H	14.38380	-49.11350	16.48290
H	13.08950	-57.95610	12.55720
H	11.94070	-60.47240	16.37480
H	17.00250	-60.14680	19.12810
H	17.42830	-58.07050	12.88390
C	16.40470	-52.58270	15.03780
H	16.47470	-51.86600	14.21710
H	15.36660	-52.90800	15.15170
H	16.75130	-52.11570	15.96510

1Zn-TS1
E B3LYP/6-31G(d): -2713.87547485691
E B3LYP/6-31G(d) in PCM ε=4: -2713.95152184311

O	18.04090	-56.76590	14.61370
P	16.41400	-55.63880	15.04100
O	16.05540	-56.62490	16.15850
O	17.42480	-54.39460	15.36620
O	14.90220	-54.66790	15.26250
O	15.97790	-55.92300	13.58700
C	17.25250	-53.16300	14.66290
C	14.57780	-54.19780	16.55350
H	17.77460	-53.20210	13.69900
H	16.19150	-52.96370	14.49610
H	17.69720	-52.37800	15.28330
H	15.19550	-53.32860	16.83680
H	13.52820	-53.87360	16.55110
H	14.70760	-54.97730	17.31480
C	12.95300	-58.06480	9.34560
C	12.20140	-56.72610	9.58600
C	11.69700	-56.45420	11.01580
C	12.69360	-56.66590	12.15080
O	13.90690	-56.90820	11.91100
O	12.25960	-56.63970	13.35630
C	12.31520	-59.25300	10.06530
O	11.41120	-59.92600	9.56850
N	12.80060	-59.45300	11.32630
C	12.30310	-60.44230	12.26540
C	10.79800	-60.29730	12.57060
O	10.47120	-59.11050	13.30440
C	10.28890	-61.47830	13.39270
C	16.95100	-57.62600	19.06700
C	16.97280	-57.04280	20.48920
O	17.69940	-57.85450	21.39300
C	18.36610	-57.73940	18.50910
O	19.32840	-58.11750	19.20540
N	18.45160	-57.36980	17.22620
C	19.62100	-57.44000	16.38500
C	19.34740	-56.59710	15.10970

C	20.37060	-56.93220	14.01820
C	17.72050	-62.06560	14.65550
C	17.75140	-60.52860	14.51320
C	16.51430	-59.93050	13.84310
O	16.43220	-58.61690	13.79170
O	15.61780	-60.63120	13.37760
C	16.81820	-62.59070	15.78200
O	17.27230	-62.95210	16.86490
N	15.48070	-62.64200	15.48730
C	14.50000	-62.89700	16.53300
C	14.26780	-61.64130	17.43640
C	13.85040	-60.39900	16.70060
N	12.55290	-60.19100	16.24160
C	12.51720	-59.04000	15.52670
N	13.71980	-58.49060	15.48110
C	14.54890	-59.31460	16.22780
Zn	14.31590	-56.90540	14.09640
H	13.99060	-57.94640	9.66500
H	12.94050	-58.28910	8.27420
H	11.32690	-56.68730	8.92430
H	12.87010	-55.91440	9.27650
H	10.81960	-57.07590	11.23490
H	11.33730	-55.41890	11.08770
H	13.59850	-58.89670	11.60770
H	12.90500	-60.36170	13.17260
H	12.45980	-61.45180	11.85660
H	10.27290	-60.28070	11.60900
H	9.24240	-61.31590	13.67120
H	10.35530	-62.40990	12.81960
H	10.87400	-61.60090	14.31350
H	16.52820	-58.63980	19.12660
H	16.31570	-57.03660	18.40130
H	15.94760	-56.96190	20.87350
H	17.38350	-56.01670	20.44980
H	17.56700	-57.13900	16.74710
H	19.84920	-58.48680	16.11940
H	20.49790	-57.06020	16.92450
H	19.47140	-55.54080	15.38740
H	20.20250	-56.29640	13.14300
H	21.40220	-56.78500	14.36790
H	20.26670	-57.97820	13.69970
H	18.72780	-62.41640	14.89520
H	17.41730	-62.50210	13.69740
H	18.62210	-60.23670	13.91030
H	17.89600	-60.04380	15.48700
H	15.19520	-62.12620	14.65680
H	14.87180	-63.71040	17.16090
H	13.56510	-63.21590	16.05820
H	15.20970	-61.44000	17.95610
H	13.52910	-61.89510	18.21120
H	11.62770	-58.69090	15.02700

H	15.57960	-59.04050	16.38140
H	11.02670	-58.36550	13.01040
H	18.51370	-58.08590	20.89320
H	17.22340	-58.02550	14.13930
H	11.75170	-60.77710	16.42690

2Zn-1
E B3LYP/6-31G(d): -2819.15878583397
E B3LYP/6-31G(d) in PCM ε=4: -2819.25452446173

C	90.76070	81.99860	100.79350
C	90.84520	83.13900	101.80440
O	90.67510	82.97260	103.01070
N	91.07960	84.37550	101.26080
C	91.18510	85.57050	102.08650
C	89.85470	85.95350	102.76260
O	88.81440	86.19250	101.80400
C	90.00330	87.21590	103.60320
C	89.35930	81.35320	100.75900
C	88.19950	82.35490	100.56770
C	88.38640	83.27940	99.37470
O	88.66320	82.80120	98.24320
O	88.29660	84.55680	99.53310
C	89.01140	89.96700	95.70880
C	88.72960	88.86720	94.69700
O	88.89050	89.01070	93.49890
C	87.75150	90.72910	96.13230
O	87.17270	91.30600	94.98280
N	88.25380	87.69510	95.26760
C	88.34680	86.48210	94.46810
C	87.33210	85.41530	94.88700
C	87.54620	84.10010	94.15130
O	87.40890	85.16480	96.32010
C	85.76180	83.78490	104.21910
C	84.99800	83.24270	102.99520
C	84.64620	84.30800	101.99800
N	83.36730	84.82210	101.83960
C	85.41400	85.01160	101.10520
C	83.38630	85.78310	100.89120
N	84.62240	85.92560	100.42580
C	94.23270	84.14120	97.34810
C	94.50140	85.61830	97.64680
O	95.24950	86.31260	96.96840
C	93.02480	83.91820	96.42000
C	91.65770	84.21560	97.02690
O	91.49200	84.32300	98.27280
O	90.68470	84.35330	96.20150
N	93.83990	86.10880	98.74700
C	93.92900	87.50700	99.11600
C	93.10260	88.46740	98.21540
C	91.67550	88.04300	98.18420

N	90.84740	88.10480	99.29980
C	90.97750	87.28110	97.28810
C	89.72770	87.38270	99.05410
N	89.77380	86.86230	97.83850
C	88.41060	92.69080	101.64180
C	88.84540	91.50300	100.76520
C	87.89500	90.34250	100.76000
N	87.71950	89.47140	101.82790
C	87.06210	89.85760	99.78700
C	86.83390	88.51510	101.48040
N	86.40440	88.72280	100.24060
Zn	89.11370	84.71940	97.43920
Zn	85.33200	87.33960	99.09530
H	89.18640	80.81770	101.69890
H	89.33810	80.61810	99.94830
H	88.07620	82.96040	101.47160
H	87.26940	81.79170	100.41190
H	89.49500	89.55870	96.60380
H	89.69860	90.67480	95.23690
H	88.03380	91.49550	96.87560
H	87.04840	90.03050	96.62750
H	86.46680	91.91300	95.25110
H	88.52390	87.55580	96.23720
H	89.35950	86.05600	94.52980
H	88.17790	86.76380	93.42620
H	86.31850	85.79210	94.70480
H	86.79850	83.36430	94.46220
H	87.45880	84.24880	93.06910
H	88.54200	83.69390	94.36190
H	86.01350	82.96650	104.90050
H	86.69430	84.26990	103.91150
H	85.16240	84.51700	104.77180
H	84.08390	82.72900	103.31880
H	85.61080	82.48510	102.49380
H	86.46600	84.90890	100.87730
H	82.51750	86.33250	100.56110
H	95.13030	83.75820	96.85500
H	94.08590	83.58260	98.27860
H	93.00180	82.86760	96.10030
H	93.12510	84.50520	95.50060
H	93.06050	85.55060	99.07650
H	94.97990	87.80450	99.05380
H	93.61820	87.60260	100.16340
H	93.51560	88.42950	97.20420
H	93.22090	89.49670	98.58130
H	91.28170	86.96430	96.30200
H	88.96920	87.21250	99.80080
H	89.14860	93.49650	101.57840
H	87.44380	93.08660	101.31460
H	88.31860	92.40630	102.69640
H	89.83980	91.15830	101.08310

H	88.96030	91.83620	99.72780
H	88.21320	89.49840	102.71090
H	86.90500	90.24390	98.79140
H	86.58180	87.67520	102.10860
H	91.05950	88.57380	100.16900
H	82.54320	84.50500	102.33370
H	86.76390	85.79550	96.77650
O	83.37620	88.13940	98.52810
O	85.80610	86.98140	97.28710
H	83.35350	87.91980	97.57810
H	83.07340	89.05850	98.60860
H	91.04090	82.34380	99.79350
H	91.48820	81.23730	101.09800
H	90.32040	88.06680	102.98190
H	90.75130	87.07850	104.39130
H	89.05250	87.46870	104.08850
H	91.52930	86.39000	101.44660
H	91.93620	85.40930	102.87050
H	88.78520	85.45180	101.15580
H	89.57080	85.11780	103.41260
H	91.20100	84.44490	100.25510
C	86.15280	86.70520	97.00610

2Zn-2
E B3LYP/6-31G(d): -3389.09189510721
E B3LYP/6-31G(d) in PCM ε=4: -3389.17266764346

C	89.63450	82.36400	100.68370
C	90.09050	83.37000	101.73640
O	89.96380	83.16860	102.94100
N	90.56740	84.55820	101.22790
C	91.18510	85.57050	102.08650
C	90.17680	86.20940	103.05600
O	89.02240	86.68040	102.33630
C	90.77300	87.39530	103.79920
C	88.10130	82.15680	100.72190
C	87.27860	83.44950	100.83580
C	87.50100	84.49050	99.75040
O	88.07150	84.12640	98.69480
O	87.13380	85.70490	99.97260
C	89.01140	89.96700	95.70880
C	89.24930	88.62320	95.06350
O	90.35660	88.27080	94.65900
C	87.85730	90.78010	95.12040
O	88.15000	91.00040	93.74890
N	88.17770	87.78790	95.04840
C	88.34680	86.48210	94.46810
C	87.19730	85.54470	94.79190
C	87.33150	84.19170	94.10480
O	87.19110	85.34870	96.23930

C	85.76180	83.78490	104.21910
C	84.25200	84.06430	104.08150
C	83.98850	84.90090	102.86770
N	82.76580	85.12940	102.25250
C	84.88690	85.56920	102.08800
C	82.96390	85.89550	101.14950
N	84.25200	86.17470	101.02030
C	93.97580	84.15000	97.29180
C	94.39670	85.58630	97.63180
O	95.30740	86.16670	97.05570
C	92.71160	84.04660	96.41820
C	91.39380	84.39200	97.10380
O	91.30100	84.49710	98.35150
O	90.38850	84.58310	96.31840
N	93.68140	86.15880	98.65570
C	93.92900	87.50700	99.11600
C	93.10650	88.61510	98.40000
C	91.65480	88.28640	98.42940
N	90.83030	88.40160	99.54330
C	90.92960	87.54630	97.54050
C	89.67510	87.73740	99.30480
N	89.70620	87.20050	98.09190
C	88.41060	92.69080	101.64180
C	86.91800	92.31800	101.73330
C	86.69890	90.84070	101.64200
N	87.07510	89.96180	102.64940
C	86.24420	90.03270	100.63370
C	86.84980	88.69520	102.24040
N	86.34610	88.70170	101.01320
Zn	88.91880	85.40250	97.39110
Zn	85.68100	87.12090	99.81000
H	87.84860	81.53960	101.59150
H	87.80250	81.59840	99.82880
H	87.48080	83.93050	101.79790
H	86.20510	83.21420	100.84080
H	88.82350	89.81440	96.78070
H	89.93900	90.53710	95.60630
H	87.78040	91.73190	95.67340
H	86.90020	90.25160	95.24820
H	87.38740	91.43460	93.33810
H	87.23520	88.10960	95.26260
H	89.29680	86.04960	94.79990
H	88.43320	86.54780	93.37190
H	86.25020	86.02780	94.53320
H	86.51100	83.52700	94.39550
H	87.30690	84.31000	93.01600
H	88.27790	83.71380	94.38040
H	85.95300	83.20730	105.12840
H	86.13500	83.20550	103.37340
H	86.33880	84.71120	104.29680
H	83.87820	84.56970	104.98300

H	83.70260	83.11580	104.01230
H	85.95520	85.62120	102.19890
H	82.17260	86.21580	100.48730
H	94.81540	83.71040	96.74750
H	93.82630	83.57670	98.21370
H	92.61110	83.01740	96.04710
H	92.79810	84.67400	95.52390
H	92.84580	85.66460	98.94840
H	94.99170	87.71430	98.96260
H	93.73290	87.54570	100.19570
H	93.42910	88.67590	97.35650
H	93.32580	89.58390	98.86800
H	91.20370	87.25250	96.53980
H	88.87910	87.65140	100.02520
H	88.54030	93.77640	101.70030
H	88.98860	92.24430	102.45930
H	88.83920	92.34720	100.69490
H	86.36350	92.79810	100.92040
H	86.49170	92.70710	102.66770
H	87.46620	90.22160	103.54490
H	85.85920	90.30280	99.66160
H	87.13290	87.82370	102.80910
H	91.04040	88.90690	100.39310
H	81.86880	84.78060	102.56480
H	86.29020	85.47700	96.60480
H	89.95000	82.67570	99.68580
H	90.12180	81.40900	100.90950
H	91.08880	88.18010	103.09950
H	91.64390	87.08760	104.38770
H	90.03870	87.82620	104.48860
H	91.60520	86.33820	101.42900
H	92.00450	85.13230	102.67220
H	88.80350	85.99800	101.67710
H	89.86580	85.43980	103.76980
H	90.81060	84.56920	100.23890
P	84.62990	87.70490	96.83070
O	85.25610	88.16940	95.55620
O	85.32430	88.06570	98.15590
O	84.54110	86.05320	96.93060
O	83.06250	88.08660	96.93850
C	82.52680	89.18770	96.17640
C	83.63100	85.35610	96.04840
H	83.83490	84.29070	96.16830
H	82.59980	85.57570	96.33400
H	83.79780	85.65170	95.00810
H	81.44440	89.04920	96.15590
H	82.76420	90.13680	96.66750
H	82.92990	89.18670	95.16120

2Zn-3D

E B3LYP/6-31G(d): -3273.65793666537
E B3LYP/6-31G(d) in PCM ε=4: -3273.82589951828

C	89.31370	82.61840	100.46160
C	89.44420	83.79950	101.42130
O	88.58500	84.04900	102.28390
N	90.57650	84.53020	101.23850
C	91.18510	85.57050	102.08650
C	90.27410	86.23290	103.13700
O	89.04560	86.68230	102.56060
C	90.97920	87.42670	103.77250
C	87.90900	82.01210	100.32700
C	86.75200	83.00680	100.04740
C	87.12910	84.15370	99.14310
O	87.60230	83.94080	97.99430
O	87.00340	85.38230	99.55030
C	89.01140	89.96700	95.70880
C	89.30910	88.58660	95.17360
O	90.42930	88.21510	94.85760
C	87.55410	90.29980	96.03860
O	86.77170	90.12530	94.86150
N	88.20750	87.75640	95.13070
C	88.34680	86.48210	94.46810
C	86.98830	85.77990	94.42890
C	87.04580	84.34220	93.94690
O	86.46750	85.77730	95.82120
C	85.76180	83.78490	104.21910
C	84.25340	84.00190	104.00430
C	84.00860	84.86640	102.80690
N	82.77650	85.21470	102.27330
C	84.91720	85.45200	101.97150
C	82.96580	85.96650	101.16430
N	84.26610	86.12700	100.94800
C	93.86810	83.95290	97.63790
C	93.77280	85.48160	97.65960
O	93.59720	86.11760	96.62470
C	92.92130	83.40980	96.56120
C	91.48470	83.81290	96.86080
O	91.04170	83.97830	97.97870
O	90.66050	83.99150	95.80130
N	93.83740	86.06400	98.89330
C	93.92900	87.50700	99.11600
C	92.89360	88.36840	98.36290
C	91.49530	87.87180	98.49350
N	90.73910	87.93960	99.65610
C	90.71280	87.18860	97.60700
C	89.56380	87.31190	99.45760
N	89.50740	86.86170	98.20680
C	88.41060	92.69080	101.64180
C	88.87270	91.38970	100.96870
C	87.88660	90.26450	101.05100

N	87.45910	89.69710	102.24340
C	87.24960	89.53940	100.07960
C	86.62750	88.67310	101.97490
N	86.47340	88.54560	100.66010
Zn	87.87100	85.91610	97.46840
Zn	85.63710	86.94280	99.65470
H	87.64600	81.46760	101.23930
H	87.95580	81.27450	99.51980
H	86.37440	83.41630	100.98090
H	85.93380	82.46260	99.55850
H	89.63980	90.11390	96.59750
H	89.38400	90.68350	94.96550
H	87.49500	91.33930	96.39160
H	87.18770	89.64690	96.84420
H	85.87820	89.82130	95.10040
H	87.35660	88.28010	94.90190
H	89.08300	85.85860	94.99460
H	88.72430	86.58560	93.44040
H	86.28130	86.37110	93.84020
H	86.04950	83.89090	93.94700
H	87.42970	84.31390	92.92130
H	87.70230	83.73760	94.58180
H	85.92220	83.14880	105.09420
H	86.23810	83.30130	103.36320
H	86.27760	84.73290	104.40330
H	83.80680	84.45910	104.89770
H	83.74840	83.03540	103.87070
H	85.99260	85.39370	102.01710
H	82.16270	86.36740	100.56330
H	94.89800	83.66740	97.39230
H	93.62670	83.51940	98.61340
H	92.97060	82.31390	96.51860
H	93.22680	83.79780	95.58470
H	94.13130	85.47700	99.66260
H	94.92740	87.87470	98.84220
H	93.82470	87.65520	100.19710
H	93.14110	88.38650	97.30180
H	92.98660	89.39510	98.74220
H	90.94560	86.93400	96.58760
H	88.84140	87.16350	100.24210
H	89.17500	93.46540	101.52990
H	87.48360	93.05990	101.19220
H	88.23530	92.55650	102.71560
H	89.82630	91.06870	101.41340
H	89.07630	91.57280	99.90800
H	87.74730	89.97910	103.17150
H	87.29540	89.66230	99.00850
H	86.17330	88.05170	102.73150
H	91.01210	88.37250	100.52760
H	81.87260	84.95020	102.64650
H	91.14940	83.88580	94.96620

H	89.69210	82.91350	99.47770
H	89.99440	81.83460	100.82240
H	91.19040	88.20520	103.02590
H	91.92770	87.13420	104.23490
H	90.34550	87.86630	104.54840
H	91.57840	86.33320	101.40810
H	92.04550	85.13430	102.61450
H	88.56310	85.83480	102.42670
H	90.05140	85.49570	103.91770
H	91.11620	84.28780	100.41380
P	85.32810	86.87150	96.37320
O	85.02490	87.92670	95.37640
O	86.03580	87.22830	97.72290
O	84.08390	85.97530	96.84380
C	83.04540	85.61010	95.89290
H	82.20030	85.26460	96.48800
H	82.76080	86.47730	95.29370
H	83.40260	84.79900	95.25270

2Zn-3E
E B3LYP/6-31G(d): -3273.64615829645
E B3LYP/6-31G(d) in PCM ε=4: -3273.82589951828

C	89.45660	82.24830	101.16890
C	89.49200	83.64330	101.79450
O	88.53950	84.07650	102.47230
N	90.65230	84.29900	101.56790
C	91.18510	85.57050	102.08650
C	90.35580	86.27550	103.17320
O	89.07090	86.67320	102.68480
C	91.08350	87.51400	103.68830
C	88.07270	81.59420	101.04620
C	86.98940	82.40820	100.28740
C	87.53730	83.18510	99.12410
O	88.15120	82.47510	98.21610
O	87.41900	84.43170	99.06100
C	89.01140	89.96700	95.70880
C	89.30710	88.57970	95.18920
O	90.44400	88.16530	94.99860
C	87.60470	90.22750	96.25340
O	86.65610	90.10850	95.19510
N	88.19400	87.80310	95.00930
C	88.34680	86.48210	94.46810
C	87.08620	85.65120	94.68490
C	87.22180	84.20110	94.26230
O	86.76820	85.67740	96.15740
C	85.76180	83.78490	104.21910
C	84.28220	83.71750	103.81000
C	84.01130	84.61800	102.64620
N	82.77020	84.85840	102.07550

C	84.88030	85.37160	101.90620
C	82.90680	85.71340	101.04060
N	84.18780	86.04590	100.90780
C	93.31600	84.25440	97.13800
C	93.51980	85.75070	97.39210
O	93.51210	86.56120	96.47170
C	91.95890	83.99090	96.46330
C	90.77550	84.09900	97.41340
O	90.89920	84.03300	98.63600
O	89.58280	84.24260	96.82960
N	93.66020	86.13310	98.69920
C	93.92900	87.50700	99.11600
C	92.93750	88.56710	98.56760
C	91.51950	88.13010	98.69390
N	90.75980	88.20650	99.85200
C	90.75080	87.41350	97.82110
C	89.59730	87.54260	99.66640
N	89.55770	87.05790	98.42990
C	88.41060	92.69080	101.64180
C	88.84220	91.21560	101.61580
C	87.70640	90.24060	101.53820
N	86.98330	89.80280	102.63850
C	87.15970	89.56870	100.47820
C	86.07400	88.89370	102.24530
N	86.15210	88.72420	100.92500
Zn	88.34020	85.62110	97.64040
Zn	85.32490	87.26380	99.77500
H	87.67220	81.37590	102.04100
H	88.21480	80.63310	100.54260
H	86.50250	83.12240	100.95020
H	86.22960	81.71510	99.90500
H	89.76100	90.18830	96.47780
H	89.20730	90.67710	94.89330
H	87.56760	91.24150	96.67680
H	87.37860	89.51300	97.05950
H	85.85730	89.65680	95.51920
H	87.33570	88.32430	94.82430
H	89.20850	85.99590	94.93610
H	88.55720	86.49130	93.38710
H	86.23140	86.12900	94.19970
H	86.30520	83.64170	94.47340
H	87.40120	84.15640	93.18240
H	88.06400	83.72080	94.76910
H	85.93860	83.12140	105.07070
H	86.43680	83.48310	103.41590
H	86.03720	84.79810	104.53140
H	83.64060	84.00150	104.65520
H	84.00570	82.68640	103.55000
H	85.95260	85.44170	102.01190
H	82.08740	86.05910	100.42750
H	94.11280	83.94120	96.45500

H	93.40280	83.66030	98.05290
H	91.93720	82.97850	96.03680
H	91.81560	84.67900	95.62400
H	93.71880	85.40980	99.40040
H	94.93760	87.81080	98.80730
H	93.91270	87.50830	100.21130
H	93.14710	88.72720	97.50970
H	93.12130	89.51110	99.09650
H	90.98770	87.16130	96.80010
H	88.89490	87.37810	100.46780
H	89.29130	93.33860	101.68620
H	87.84140	92.94980	100.74380
H	87.78720	92.91490	102.51430
H	89.45030	90.98810	102.50190
H	89.48500	91.04190	100.74510
H	87.13950	90.08940	103.59740
H	87.42720	89.62200	99.43370
H	85.39780	88.38310	102.91470
H	91.03310	88.65390	100.71670
H	81.89180	84.45670	102.38290
H	88.66530	83.06850	97.57380
H	89.94910	82.28460	100.19080
H	90.08480	81.60130	101.79610
H	91.27150	88.23680	102.88220
H	92.04700	87.25610	104.13910
H	90.47330	88.01100	104.44790
H	91.33050	86.24370	101.23750
H	92.17620	85.35140	102.50710
H	88.60680	85.80510	102.60820
H	90.22040	85.58030	104.01280
H	91.20550	83.91420	100.80970
P	85.59490	86.67850	96.75930
O	85.16790	87.73800	95.81200
O	86.27610	87.05680	98.11700
O	84.39430	85.71660	97.24740
C	83.38570	85.28490	96.29330
H	82.57450	84.85650	96.88210
H	83.02720	86.13600	95.71040
H	83.80390	84.51770	95.63590

2Zn-3B

E B3LYP/6-31G(d): -3273.34942966683

E B3LYP/6-31G(d) in PCM ε=4: -3273.42725211355

C	-0.15340	-1.51840	0.26280
C	-0.44840	-1.63190	1.75690
O	0.09320	-2.50660	2.45890
N	-1.32100	-0.70450	2.21990
C	-1.66690	-0.34330	3.60440
C	-1.28360	-1.35450	4.69990

O	-1.75700	-2.66580	4.39370
C	-1.86520	-0.93330	6.04620
C	0.27580	-2.82860	-0.42180
C	-0.62510	-4.06550	-0.16080
C	-2.09130	-3.71550	-0.03290
O	-2.60950	-3.01420	-0.94260
O	-2.76220	-4.05700	1.00920
C	-9.04070	-2.55840	1.26980
C	-8.44420	-1.97750	0.00720
O	-8.31720	-0.76460	-0.16150
C	-8.75280	-4.03230	1.57340
O	-9.33760	-4.86730	0.57420
N	-7.99300	-2.89870	-0.88130
C	-7.21270	-2.49090	-2.01330
C	-6.26200	-3.58820	-2.47790
C	-5.30760	-3.13130	-3.57030
O	-5.46790	-4.02680	-1.29740
C	1.40140	-5.57690	3.01790
C	1.21690	-7.06610	2.67880
C	-0.21640	-7.32980	2.34750
N	-0.76850	-8.49000	1.82660
C	-1.26210	-6.45560	2.41070
C	-2.08700	-8.28050	1.58410
N	-2.41500	-7.04380	1.92580
C	-4.20680	2.98930	-0.46090
C	-5.01250	3.13730	0.83570
O	-5.96380	3.89960	0.94450
C	-4.58020	1.75010	-1.29650
C	-4.08930	0.40490	-0.76100
O	-3.21990	0.32990	0.13550
O	-4.64860	-0.62690	-1.30730
N	-4.56640	2.35650	1.87650
C	-5.24200	2.32540	3.15710
C	-6.38900	1.27830	3.26400
C	-5.91660	-0.06700	2.82800
N	-5.05510	-0.88310	3.55370
C	-5.94690	-0.62600	1.58320
C	-4.58950	-1.86570	2.74490
N	-5.12320	-1.74120	1.53820
C	-7.12400	-4.22280	7.31440
C	-6.37850	-3.34980	6.29590
C	-5.41730	-4.11670	5.44330
N	-4.16460	-4.53090	5.87420
C	-5.50060	-4.55940	4.15100
C	-3.53760	-5.15930	4.85960
N	-4.32370	-5.19690	3.79170
Zn	-4.41670	-2.34220	-0.32610
Zn	-3.87040	-5.57770	1.78690
H	1.29040	-3.09460	-0.10750
H	0.31650	-2.62970	-1.49720
H	-0.31310	-4.58280	0.74220

H	-0.52120	-4.76360	-1.00150
H	-8.68670	-1.93160	2.09720
H	-10.12910	-2.41050	1.23020
H	-9.18500	-4.27910	2.55510
H	-7.67010	-4.19650	1.62900
H	-8.69500	-5.54660	0.29510
H	-8.38150	-3.83690	-0.81750
H	-6.66840	-1.57880	-1.75410
H	-7.84380	-2.22180	-2.87650
H	-6.83110	-4.46250	-2.79930
H	-4.61680	-3.93350	-3.84770
H	-5.88370	-2.85590	-4.46100
H	-4.72820	-2.25560	-3.26190
H	2.44820	-5.38200	3.27080
H	1.13520	-4.91810	2.19100
H	0.80160	-5.28790	3.88730
H	1.54250	-7.69110	3.52190
H	1.85440	-7.34160	1.82750
H	-1.24400	-5.42160	2.71340
H	-2.75070	-9.02540	1.17040
H	-4.41470	3.88680	-1.04870
H	-3.13430	2.96480	-0.23700
H	-4.15850	1.85470	-2.30570
H	-5.66490	1.68290	-1.43640
H	-3.89840	1.63190	1.63490
H	-5.66260	3.31950	3.32990
H	-4.49450	2.13560	3.93850
H	-7.20790	1.58590	2.60690
H	-6.77650	1.27380	4.29160
H	-6.51300	-0.29730	0.72660
H	-3.83570	-2.57210	3.05380
H	-7.81930	-3.61640	7.90400
H	-7.69820	-5.00640	6.80990
H	-6.43260	-4.70990	8.01180
H	-5.84490	-2.54380	6.82000
H	-7.09960	-2.86080	5.63160
H	-3.75380	-4.33990	6.77820
H	-6.31320	-4.45160	3.44880
H	-2.53140	-5.54260	4.92290
H	-4.80540	-0.77130	4.52640
H	-0.27590	-9.35650	1.65150
H	-1.01690	-1.08330	-0.24990
H	0.66150	-0.78870	0.15570
H	-2.96370	-0.94020	6.02040
H	-1.53820	0.07260	6.32920
H	-1.54360	-1.62930	6.82720
H	-2.74430	-0.16930	3.62510
H	-1.18320	0.61510	3.84460
H	-1.14420	-2.93650	3.67150
H	-0.18990	-1.38030	4.78120
H	-1.80660	-0.16190	1.50700

P	-5.84890	-5.49910	-0.59230
O	-7.19390	-5.96170	-1.03350
O	-5.56980	-5.29500	0.90190
O	-4.62950	-6.46550	-1.06230
C	-4.64740	-7.05390	-2.37900
H	-3.90280	-7.85160	-2.37350
H	-5.63590	-7.46610	-2.59750
H	-4.37260	-6.30780	-3.13170

2Zn-4D

E B3LYP/6-31G(d): -3956.36389047902

E B3LYP/6-31G(d) in PCM ε=4: -3956.43090056175

C	-1.02130	-0.05310	-0.15080
C	-0.71230	-0.08380	1.34110
O	0.42710	0.01540	1.78710
N	-1.79320	-0.30300	2.15610
C	-1.66690	-0.34330	3.60440
C	-1.15750	-1.69200	4.15930
O	-2.02040	-2.78430	3.83750
C	-1.04710	-1.63510	5.67760
C	-0.36490	-1.24580	-0.88180
C	-0.60110	-2.60990	-0.20690
C	-2.06000	-2.94730	0.04530
O	-2.93500	-2.67800	-0.83850
O	-2.41670	-3.51140	1.13630
C	-9.04070	-2.55840	1.26980
C	-8.40180	-1.98950	0.02000
O	-8.20390	-0.76850	-0.14590
C	-8.74400	-4.01360	1.64630
O	-9.43640	-4.88330	0.77610
N	-8.02850	-2.89170	-0.89800
C	-7.21270	-2.49090	-2.01330
C	-6.37870	-3.66460	-2.53490
C	-5.43200	-3.25470	-3.65410
O	-5.59590	-4.18630	-1.42930
C	1.40140	-5.57690	3.01790
C	1.21070	-7.02970	2.56550
C	-0.21750	-7.35720	2.24740
N	-0.65540	-8.65680	2.02880
C	-1.34650	-6.58740	2.12140
C	-1.98920	-8.64000	1.79640
N	-2.43970	-7.39360	1.84460
C	-6.80940	3.74520	0.00710
C	-6.61120	3.44380	1.48960
O	-7.45870	3.71710	2.33460
C	-7.17280	2.45900	-0.75040
C	-5.95550	1.60470	-1.07860
O	-4.79880	1.93940	-0.90940
O	-6.22420	0.39590	-1.60640
N	-5.44450	2.80110	1.80500

C	-5.24200	2.32540	3.15710
C	-6.11690	1.08820	3.51420
C	-5.67170	-0.23110	2.95330
N	-5.08850	-1.20590	3.75540
C	-5.69110	-0.79290	1.69880
C	-4.78930	-2.28470	3.00220
N	-5.13260	-2.06600	1.74260
C	-7.12400	-4.22280	7.31440
C	-6.85730	-3.87170	5.84290
C	-5.86090	-4.73710	5.13010
N	-4.48700	-4.73250	5.36470
C	-6.02470	-5.61030	4.08360
C	-3.89460	-5.56180	4.46580
N	-4.80330	-6.10850	3.67870
Zn	-4.47770	-3.48110	0.30140
Zn	-4.60310	-7.26810	1.86010
H	0.71670	-1.07920	-0.93130
H	-0.74560	-1.27610	-1.90740
H	-0.05910	-2.67030	0.74180
H	-0.19920	-3.40360	-0.85170
H	-8.76460	-1.88630	2.09090
H	-10.13250	-2.47240	1.16490
H	-9.09000	-4.16980	2.68160
H	-7.66280	-4.20630	1.63190
H	-8.79070	-5.52610	0.41390
H	-8.29900	-3.86820	-0.78460
H	-6.52810	-1.70790	-1.69600
H	-7.81820	-2.08740	-2.84130
H	-7.04560	-4.46340	-2.87390
H	-4.85190	-4.11990	-3.98800
H	-5.99670	-2.86520	-4.50950
H	-4.73570	-2.48180	-3.31250
H	2.44980	-5.38680	3.26870
H	1.11310	-4.87530	2.22750
H	0.79180	-5.35640	3.90070
H	1.56940	-7.70510	3.35650
H	1.84740	-7.22650	1.69010
H	-1.44410	-5.51320	2.17600
H	-2.62930	-9.49890	1.63140
H	-7.62250	4.47090	-0.06300
H	-5.90700	4.18670	-0.42930
H	-7.65670	2.70320	-1.70620
H	-7.89490	1.85290	-0.18600
H	-4.85480	2.47670	1.04620
H	-5.53300	3.13160	3.83620
H	-4.17620	2.11910	3.30160
H	-7.14220	1.30910	3.20220
H	-6.14830	1.00220	4.60920
H	-6.12490	-0.40860	0.79240
H	-4.28930	-3.15550	3.38470
H	-7.87160	-3.54360	7.73780

H	-7.49910	-5.24710	7.40810
H	-6.21580	-4.14290	7.92470
H	-6.54760	-2.81800	5.77130
H	-7.79480	-3.94060	5.27980
H	-3.97520	-4.16190	6.02470
H	-6.93230	-5.91770	3.58310
H	-2.82780	-5.71690	4.40850
H	-4.89160	-1.11720	4.74200
H	-0.08240	-9.48850	2.05320
H	-7.15320	0.14710	-1.41910
H	-2.10050	-0.04560	-0.33750
H	-0.61860	0.88250	-0.55480
H	-2.01390	-1.36340	6.12400
H	-0.30460	-0.89740	5.99750
H	-0.77160	-2.61740	6.07460
H	-2.64650	-0.11650	4.03710
H	-0.96140	0.43750	3.91280
H	-1.98550	-2.95290	2.87200
H	-0.16300	-1.87590	3.72910
H	-2.71770	-0.34010	1.75040
P	-5.95860	-5.70180	-0.72940
O	-7.44440	-5.82330	-0.77360
O	-5.09430	-5.41630	0.54500
O	-5.17530	-6.74450	-1.64200
C	-5.87070	-7.85450	-2.29590
H	-5.19220	-8.17760	-3.08700
H	-6.04710	-8.65810	-1.57290
H	-6.81410	-7.50510	-2.72350
O	-4.65780	-9.33550	1.83200
P	-6.10580	-9.33450	1.21590
O	-6.34550	-10.08750	-0.06240
O	-6.43090	-7.77650	1.16460
O	-7.03280	-9.95830	2.43480
C	-8.38940	-10.25010	2.12610
H	-8.81270	-10.77870	2.98730
H	-8.96660	-9.32950	1.95670
H	-8.46310	-10.88230	1.23420

2Zn-4E
E B3LYP/6-31G(d): -3956.37035730048
E B3LYP/6-31G(d) in PCM ε=4: -3956.43614633002

C	-0.38960	-0.53070	-0.00190
C	-0.33150	-0.54100	1.52760
O	0.72080	-0.72850	2.13800
N	-1.53480	-0.36580	2.15060
C	-1.66690	-0.34330	3.60440
C	-1.32520	-1.71160	4.22810
O	-2.09310	-2.76150	3.62260
C	-1.60310	-1.74340	5.72540
C	0.16060	-1.83470	-0.60860

C	-0.53480	-3.12600	-0.11240
C	-2.02160	-3.17410	-0.37030
O	-2.33750	-3.41290	-1.63050
O	-2.85700	-3.00190	0.52850
C	-9.04070	-2.55840	1.26980
C	-8.31430	-2.00190	0.05930
O	-8.43400	-0.82640	-0.27140
C	-8.53080	-3.88980	1.84610
O	-8.68390	-4.90360	0.87840
N	-7.51540	-2.89750	-0.64550
C	-7.21270	-2.49090	-2.01330
C	-6.16440	-3.35420	-2.75360
C	-5.45410	-2.56210	-3.83980
O	-5.14300	-3.87370	-1.82380
C	1.40140	-5.57690	3.01790
C	1.07900	-6.87980	2.26830
C	-0.38080	-7.06840	1.96110
N	-0.89210	-8.27550	1.50090
C	-1.47530	-6.24060	2.02430
C	-2.22990	-8.14720	1.31540
N	-2.61380	-6.91920	1.62040
C	-5.14610	2.61760	-0.63660
C	-5.66800	2.84160	0.78800
O	-6.69610	3.46380	1.03890
C	-5.36250	1.18630	-1.16670
C	-4.50940	0.08180	-0.52660
O	-3.55690	0.37210	0.23200
O	-4.85310	-1.11170	-0.85190
N	-4.88250	2.28840	1.76160
C	-5.24200	2.32540	3.15710
C	-6.42480	1.40500	3.56540
C	-6.12140	-0.00960	3.22370
N	-5.33270	-0.85850	3.99220
C	-6.25810	-0.65520	2.02970
C	-5.02670	-1.95570	3.25600
N	-5.57470	-1.86020	2.05650
C	-7.12400	-4.22280	7.31440
C	-7.03800	-3.80200	5.85090
C	-6.00140	-4.56110	5.09440
N	-4.64220	-4.54030	5.39400
C	-6.10220	-5.35840	3.98900
C	-3.98660	-5.28080	4.46260
N	-4.84700	-5.77550	3.59390
Zn	-5.03600	-2.79370	0.25240
Zn	-4.80020	-6.66540	1.66770
H	1.22010	-1.92720	-0.34760
H	0.09330	-1.78180	-1.70110
H	-0.38220	-3.23240	0.96460
H	-0.08030	-3.98950	-0.61040
H	-9.04290	-1.77780	2.03760
H	-10.08740	-2.69380	0.96070

H	-9.11470	-4.10990	2.75850
H	-7.48290	-3.77280	2.15370
H	-8.09180	-5.68420	1.06670
H	-7.80640	-3.87960	-0.50120
H	-6.86860	-1.46070	-1.97040
H	-8.12590	-2.50600	-2.62630
H	-6.65430	-4.23110	-3.17610
H	-4.76400	-3.20260	-4.39930
H	-6.19050	-2.16080	-4.54630
H	-4.90100	-1.72440	-3.40480
H	2.47390	-5.51520	3.22640
H	1.12620	-4.69640	2.42790
H	0.86430	-5.52650	3.97110
H	1.43140	-7.73420	2.86440
H	1.65950	-6.91020	1.33400
H	-1.52030	-5.21030	2.34700
H	-2.91000	-8.93060	1.00470
H	-5.68690	3.32010	-1.27620
H	-4.07830	2.86260	-0.68050
H	-5.14340	1.16600	-2.24330
H	-6.41420	0.88910	-1.07560
H	-4.14060	1.67190	1.43900
H	-5.51870	3.35310	3.41990
H	-4.34970	2.06640	3.73970
H	-7.31300	1.73280	3.01870
H	-6.62800	1.53620	4.63670
H	-6.80810	-0.32660	1.16340
H	-4.37220	-2.73880	3.59870
H	-7.90740	-3.66530	7.84020
H	-7.35240	-5.29020	7.40000
H	-6.18250	-4.04180	7.84900
H	-6.84620	-2.72070	5.78110
H	-7.99880	-3.97030	5.35310
H	-4.20520	-4.07270	6.17540
H	-6.96970	-5.68450	3.43450
H	-2.91490	-5.40000	4.43720
H	-5.02660	-0.68810	4.93970
H	-0.36210	-9.12210	1.34840
H	-3.31760	-3.49530	-1.76570
H	-1.40620	-0.32720	-0.35100
H	0.24360	0.29770	-0.34320
H	-2.66240	-1.53680	5.93480
H	-1.00010	-0.99580	6.25220
H	-1.35720	-2.72760	6.13920
H	-2.69980	-0.06610	3.83630
H	-0.99750	0.41430	4.03180
H	-2.13100	-2.59870	2.66000
H	-0.25910	-1.89280	4.05020
H	-2.35640	-0.21410	1.56310
P	-5.56220	-5.34070	-1.04400
O	-6.94890	-5.67420	-1.43360

O	-5.10330	-4.89050	0.38170
O	-4.45780	-6.41360	-1.49200
C	-4.88560	-7.57390	-2.27250
H	-4.00080	-8.20900	-2.33550
H	-5.70250	-8.09170	-1.76000
H	-5.18750	-7.25370	-3.27370
O	-5.08250	-8.61790	1.28670
P	-6.63800	-8.47380	1.05650
O	-7.18420	-8.93090	-0.26200
O	-6.87140	-6.93970	1.44140
O	-7.28560	-9.35140	2.29740
C	-8.68270	-9.61150	2.23920
H	-8.91590	-10.31490	3.04570
H	-9.26670	-8.69200	2.39180
H	-8.96190	-10.04970	1.27470

2Zn-TS1

E B3LYP/6-31G(d): -3389.04061872

E B3LYP/6-31G(d) in PCM ε=4: -3389.11760535781

O	86.67860	85.96420	96.21380
P	85.24140	87.35610	97.50170
O	86.60370	87.85670	98.01170
O	84.86640	87.91470	96.03930
O	84.25020	88.39480	98.48770
O	84.67610	86.06900	98.03270
C	83.56800	87.58130	95.48130
C	84.31610	89.82430	98.34520
H	83.47850	86.49820	95.37550
H	82.77020	87.96540	96.12210
H	83.53350	88.06310	94.50400
H	83.99780	90.11400	97.33910
H	83.62910	90.25680	99.07550
H	85.32930	90.19570	98.52810
C	88.95910	82.79140	100.62310
C	89.33240	83.85160	101.66310
O	88.72200	83.94290	102.74430
N	90.37780	84.62940	101.29360
C	91.18510	85.57050	102.08650
C	90.57690	86.01210	103.42770
O	89.28320	86.58630	103.23400
C	91.46210	87.04800	104.11370
C	87.45810	82.47360	100.45270
C	86.47550	83.66380	100.47230
C	86.91420	84.84420	99.64420
O	87.36970	84.67960	98.48950
O	86.89450	86.03630	100.17390
C	89.01140	89.96700	95.70880
C	89.31750	88.55140	95.29450
O	90.44710	88.07060	95.41930

C	87.63670	90.30500	96.27980
O	86.61830	90.13990	95.28030
N	88.27140	87.88410	94.76720
C	88.34680	86.48210	94.46810
C	87.03050	85.76320	94.82440
C	87.08570	84.27580	94.49350
C	85.76180	83.78490	104.21910
C	84.27310	83.62370	103.86530
C	83.87800	84.61040	102.81160
N	82.93560	84.39410	101.81670
C	84.44160	85.81330	102.48970
C	82.97570	85.42150	100.93740
N	83.88620	86.30710	101.32220
C	93.12320	84.82800	96.53170
C	93.79420	86.07960	97.10740
O	94.62060	86.74670	96.49770
C	91.62850	85.01630	96.20490
C	90.69800	85.18810	97.40990
O	91.03750	84.76510	98.53870
O	89.58610	85.77820	97.14760
N	93.39140	86.39040	98.38210
C	93.92900	87.50700	99.11600
C	93.21340	88.86840	98.87620
C	91.76890	88.77000	99.23140
N	91.26140	88.74500	100.52580
C	90.73200	88.35300	98.44690
C	89.98240	88.29550	100.49030
N	89.62590	88.05560	99.23250
C	88.41060	92.69080	101.64180
C	88.32670	91.45250	102.55050
C	87.20610	90.51380	102.20530
N	86.12440	90.27890	103.03860
C	86.99710	89.68510	101.13140
C	85.33030	89.33810	102.48130
N	85.83960	88.95200	101.31820
Zn	88.29240	86.64960	98.38480
Zn	85.24250	87.33520	100.12260
H	87.13720	81.79170	101.24840
H	87.35010	81.92820	99.50950
H	86.32120	84.01360	101.48920
H	85.50680	83.32540	100.07880
H	89.78470	90.26950	96.42320
H	89.17290	90.60450	94.82570
H	87.64940	91.35680	96.59960
H	87.42300	89.68380	97.15120
H	85.90430	89.59660	95.64970
H	87.41970	88.41620	94.61520
H	89.16830	86.05980	95.04080
H	88.53770	86.31200	93.39650
H	86.21200	86.23350	94.26390
H	86.13120	83.79070	94.72320

H	87.29740	84.12240	93.42980
H	87.87790	83.78210	95.06920
H	86.02170	83.10130	105.03460
H	86.43310	83.57300	103.38770
H	85.97580	84.79700	104.57980
H	83.65560	83.74970	104.76550
H	84.07950	82.60500	103.50410
H	85.24950	86.32720	102.98680
H	82.37500	85.48270	100.04220
H	93.66060	84.58650	95.61120
H	93.24120	83.99220	97.23140
H	91.27460	84.13480	95.65070
H	91.47960	85.88000	95.54990
H	92.67910	85.79860	98.79610
H	94.97870	87.62870	98.82870
H	93.90380	87.26230	100.18540
H	93.29820	89.12030	97.81510
H	93.73050	89.65050	99.44800
H	90.73780	88.21510	97.37340
H	89.40090	88.08420	101.37850
H	89.25400	93.32220	101.93960
H	88.55670	92.40310	100.59540
H	87.49770	93.29300	101.70200
H	88.22550	91.76310	103.59810
H	89.27130	90.89720	102.49360
H	85.95620	90.73720	103.92470
H	87.60110	89.55630	100.24650
H	84.42200	88.96650	102.93240
H	91.76870	88.97960	101.36870
H	82.31650	83.59610	101.75470
H	89.40750	83.05360	99.65990
H	89.46480	81.86910	100.94190
H	91.56560	87.95120	103.49670
H	92.46420	86.65260	104.31060
H	91.01450	87.34780	105.06590
H	91.36250	86.44630	101.46040
H	92.16280	85.10510	102.28350
H	88.71580	85.79830	103.08900
H	90.48410	85.13550	104.07880
H	90.66990	84.55120	100.31640
H	86.89630	85.19140	96.78220

1Zn-2u

E B3LYP/6-31G(d): -2713.99462739064

C	16.60010	-60.57600	9.58720
C	16.67690	-59.11540	9.10080
C	15.65490	-58.18450	9.78100
C	15.78830	-58.17010	11.29850
O	16.89690	-57.84840	11.81810
O	14.79170	-58.49090	12.02660
C	15.19840	-61.15130	9.39020

O	14.67320	-61.21600	8.27530
N	14.57660	-61.54580	10.53270
C	13.20070	-62.00600	10.53310
C	12.18980	-60.84890	10.65760
O	12.41560	-60.08950	11.83840
C	10.75540	-61.36540	10.71540
C	18.38820	-55.54320	19.10070
C	19.10110	-54.83260	20.26310
O	19.89900	-55.71720	21.02490
C	19.39820	-56.13770	18.12320
O	20.42400	-56.71580	18.53250
N	19.10910	-55.98680	16.81930
C	19.90920	-56.58760	15.76730
C	19.03780	-56.93400	14.55570
C	19.85400	-57.46070	13.37750
O	18.01450	-57.87320	14.94240
C	16.95840	-63.74240	14.82710
C	17.50500	-62.30080	14.81970
C	16.77470	-61.30240	13.89590
O	17.12950	-60.08590	14.04180
O	15.92630	-61.73500	13.08130
C	15.62910	-63.89710	15.57370
O	15.55290	-64.38770	16.70040
N	14.53830	-63.45220	14.88180
C	13.23150	-63.36730	15.50260
C	13.07970	-62.17970	16.49330
C	13.43380	-60.85830	15.89210
N	12.65880	-60.19340	14.94850
C	13.35280	-59.11640	14.51190
N	14.52070	-59.02680	15.12200
C	14.57350	-60.10180	15.99060
Zn	16.12590	-58.16270	13.82580
H	16.90810	-60.64380	10.63640
H	17.29710	-61.18440	8.99670
H	16.50050	-59.09620	8.01910
H	17.68730	-58.73360	9.28540
H	14.63520	-58.47430	9.51090
H	15.81430	-57.15900	9.42270
H	15.04690	-61.42800	11.43210
H	13.06800	-62.69240	11.37910
H	13.01940	-62.55310	9.60170
H	12.30880	-60.21000	9.76840
H	10.05290	-60.52930	10.79940
H	10.50610	-61.94250	9.81750
H	10.61790	-62.01390	11.59090
H	17.79660	-56.37180	19.51370
H	17.69940	-54.86660	18.58250
H	18.35580	-54.39650	20.94030
H	19.69910	-53.99480	19.85910
H	18.22690	-55.53060	16.54450
H	20.40420	-57.48070	16.16580

H	20.70460	-55.89890	15.44140
H	18.48170	-56.04190	14.25430
H	19.19320	-57.69320	12.53810
H	20.58670	-56.71260	13.05190
H	20.40620	-58.36890	13.65770
H	17.67130	-64.40030	15.33280
H	16.84380	-64.08000	13.79100
H	18.55480	-62.31820	14.49550
H	17.51730	-61.88460	15.83430
H	14.74540	-62.87840	14.06210
H	13.04760	-64.29760	16.04930
H	12.48510	-63.29190	14.70260
H	13.74930	-62.36640	17.33820
H	12.05290	-62.17960	16.88780
H	12.99870	-58.46000	13.73410
H	15.43450	-60.27350	16.62030
H	13.27290	-59.61410	11.75210
H	20.37540	-56.24590	20.34760
H	11.84140	-60.54520	14.46960
H	18.30990	-58.79910	14.82730
P	15.53020	-55.08350	15.18350
O	16.70290	-54.75400	16.06160
O	15.09680	-53.74670	14.36220
O	14.16900	-55.28480	16.08150
O	15.63220	-56.22610	14.17670
C	14.09250	-53.84930	13.35550
C	14.24360	-56.09570	17.25120
H	14.04940	-52.87910	12.85070
H	14.34050	-54.63180	12.63100
H	13.11310	-54.06590	13.80080
H	13.27850	-56.00910	17.76080
H	14.41760	-57.14700	16.99460
H	15.03930	-55.74590	17.91840

1Zn-2u

E B3LYP/6-31G(d): -2713.92274008592

O	19.89600	-58.07340	14.64170
P	19.02930	-56.89090	16.05140
O	19.11870	-58.07840	17.00810
O	20.41720	-55.98590	15.91530
O	18.30560	-55.72380	17.15520
O	17.93720	-56.57040	14.97640
C	20.35060	-54.69730	15.31770
C	18.87190	-55.63550	18.44460
H	20.13430	-54.77300	14.24190
H	19.58890	-54.07540	15.80040
H	21.33700	-54.23380	15.44590
H	19.88560	-55.18900	18.42940
H	18.23050	-54.98210	19.05810
H	18.94400	-56.62350	18.92370
C	17.70340	-60.47910	10.54320

C	17.30190	-59.21890	9.74670
C	16.02010	-58.50410	10.23120
C	16.11080	-57.94230	11.64690
O	17.17900	-57.39380	12.03000
O	15.10880	-58.04230	12.44470
C	16.59140	-61.52570	10.53140
O	16.21690	-62.06450	9.47590
N	16.01680	-61.77970	11.73330
C	14.86970	-62.65920	11.89200
C	13.54680	-61.94650	11.56750
O	13.32390	-60.82790	12.44170
C	12.36350	-62.89540	11.73320
C	21.84980	-60.33320	18.78670
C	21.89800	-61.71870	19.44170
O	21.00000	-62.66940	18.87950
C	22.32800	-60.34650	17.33320
O	23.32350	-61.00180	16.98490
N	21.60080	-59.59000	16.48490
C	21.88480	-59.51250	15.06080
C	21.28560	-58.24000	14.42870
C	21.57840	-58.25950	12.92300
C	18.67020	-62.45480	16.42890
C	18.89490	-61.11030	15.71170
C	17.81160	-60.68370	14.73720
O	17.97210	-59.52300	14.13020
O	16.83450	-61.39960	14.48440
C	17.72790	-62.33830	17.62130
O	18.13390	-62.22530	18.78960
N	16.40850	-62.37100	17.29940
C	15.33940	-62.08110	18.24460
C	15.14260	-60.57250	18.54620
C	14.95570	-59.72510	17.32460
N	13.86450	-59.80610	16.46740
C	14.09130	-58.99410	15.40880
N	15.26510	-58.39000	15.53100
C	15.80980	-58.83630	16.72360
Zn	16.36380	-57.28530	14.07750
H	17.98570	-60.19870	11.56360
H	18.58480	-60.92790	10.06570
H	17.16860	-59.50310	8.69580
H	18.13750	-58.51030	9.78940
H	15.14370	-59.15870	10.14740
H	15.83040	-57.64700	9.56680
H	16.40290	-61.39450	12.59710
H	14.85240	-62.98650	12.93560
H	14.98720	-63.53650	11.24180
H	13.60060	-61.60390	10.52380
H	11.42280	-62.38110	11.51220
H	12.45870	-63.75810	11.05770
H	12.31310	-63.26670	12.76320
H	20.84410	-59.90360	18.86660

H	22.52510	-59.67060	19.34740
H	21.72220	-61.60730	20.52320
H	22.90140	-62.13580	19.31000
H	20.76820	-59.08170	16.80680
H	21.49660	-60.40130	14.53880
H	22.97180	-59.52300	14.91970
H	21.79940	-57.38630	14.87990
H	21.23760	-57.32950	12.46510
H	22.64960	-58.38010	12.71660
H	21.04680	-59.08760	12.44220
H	19.63080	-62.80240	16.81620
H	18.29040	-63.19810	15.72020
H	19.81980	-61.18310	15.13130
H	19.04190	-60.28660	16.41850
H	16.20600	-62.29020	16.30480
H	15.55520	-62.60470	19.18110
H	14.41870	-62.51300	17.83750
H	16.02820	-60.20560	19.07380
H	14.29510	-60.47440	19.23930
H	13.40940	-58.89390	14.57720
H	16.78480	-58.50330	17.05850
H	14.03940	-60.17520	12.30420
H	20.07360	-62.34950	18.97350
H	13.01130	-60.33460	16.61800
H	18.89440	-58.96990	14.35640

2Zn-2p

E B3LYP/6-31G(d): -2819.50379369929

C	0.25380	-1.57670	-0.15750
C	0.05590	-1.59720	1.35790
O	0.75110	-2.27820	2.11240
N	-0.97140	-0.81320	1.80580
C	-1.25010	-0.53430	3.21050
C	-1.42760	-1.78210	4.10080
O	-2.25690	-2.78420	3.48790
C	-2.03810	-1.41680	5.44680
C	0.49940	-2.96440	-0.77800
C	-0.52640	-4.05380	-0.37360
C	-1.95330	-3.55170	-0.41710
O	-2.44530	-3.15370	-1.52220
O	-2.64570	-3.44540	0.65880
C	-9.04070	-2.55840	1.26980
C	-8.81150	-2.87870	-0.19700
O	-9.68480	-3.33250	-0.91810
C	-8.12170	-3.26690	2.26210
O	-7.91360	-4.66810	1.95070
N	-7.53630	-2.62280	-0.61320
C	-7.21270	-2.49090	-2.01330
C	-6.24790	-3.57670	-2.50110
C	-5.69130	-3.28770	-3.88530
O	-5.13260	-3.68400	-1.52190

C	1.40140	-5.57690	3.01790
C	1.14700	-6.82010	2.15330
C	-0.30490	-7.03280	1.84660
N	-0.78840	-8.14530	1.17110
C	-1.41840	-6.27670	2.10400
C	-2.12620	-8.04560	1.04070
N	-2.54780	-6.91390	1.59920
C	-3.62330	2.76430	-0.30810
C	-4.74780	2.59610	0.71670
O	-5.93200	2.61520	0.39280
C	-3.84030	1.85240	-1.52960
C	-3.79210	0.37650	-1.18100
O	-2.94670	-0.06610	-0.33350
O	-4.62460	-0.43160	-1.71490
N	-4.34350	2.37190	2.00690
C	-5.24200	2.32540	3.15710
C	-6.45880	1.38230	3.01700
C	-6.02340	0.05750	2.50510
N	-5.34390	-0.88370	3.26290
C	-5.92050	-0.39330	1.21930
C	-4.84790	-1.83720	2.44110
N	-5.18740	-1.57090	1.18620
C	-7.12400	-4.22280	7.31440
C	-5.81220	-3.63200	6.77230
C	-5.16000	-4.47440	5.71080
N	-3.90000	-5.03030	5.86030
C	-5.56050	-4.86550	4.45730
C	-3.56620	-5.69630	4.74070
N	-4.56040	-5.62110	3.85830
Zn	-3.93770	-2.00150	-0.50170
Zn	-4.45730	-6.23100	1.89430
H	1.49020	-3.32750	-0.48760
H	0.49830	-2.85040	-1.86650
H	-0.31170	-4.40430	0.63490
H	-0.43100	-4.90020	-1.06480
H	-8.95520	-1.47670	1.43930
H	-10.08300	-2.82490	1.47890
H	-8.54170	-3.17990	3.27210
H	-7.11930	-2.84700	2.27320
H	-8.76020	-5.05710	1.67310
H	-6.92310	-2.15620	0.03790
H	-6.77460	-1.50460	-2.19780
H	-8.14050	-2.57910	-2.58650
H	-6.75340	-4.54880	-2.47410
H	-5.02390	-4.08850	-4.22610
H	-6.51040	-3.21790	-4.60940
H	-5.14350	-2.33920	-3.89400
H	2.46770	-5.50660	3.25090
H	1.11480	-4.64510	2.52230
H	0.86220	-5.64670	3.96950
H	1.53710	-7.71190	2.66370

H	1.70560	-6.74160	1.21010
H	-1.48030	-5.30730	2.57500
H	-2.74600	-8.78980	0.56270
H	-3.63990	3.80800	-0.64370
H	-2.64050	2.57380	0.13440
H	-3.05650	2.04790	-2.27270
H	-4.80300	2.06760	-1.99790
H	-3.35850	2.47450	2.20700
H	-5.62510	3.32980	3.38450
H	-4.63150	2.01870	4.01370
H	-7.16760	1.81630	2.31210
H	-6.94980	1.31010	3.99590
H	-6.26350	0.09690	0.32050
H	-4.20640	-2.63010	2.78880
H	-7.54810	-3.56570	8.07980
H	-7.86890	-4.33720	6.51960
H	-6.95920	-5.20620	7.76660
H	-5.10390	-3.48720	7.59710
H	-6.00600	-2.63130	6.36460
H	-3.30870	-4.93340	6.67660
H	-6.50130	-4.69120	3.95750
H	-2.62330	-6.20000	4.59590
H	-5.18600	-0.84170	4.26100
H	-0.22600	-8.91660	0.83130
H	-4.30940	-3.92280	-1.99790
O	-5.52030	-8.06840	1.61680
O	-5.71160	-5.37280	0.56760
H	-5.42900	-4.76910	-0.17500
H	-6.22460	-8.07990	0.94670
H	-5.76980	-8.70380	2.30870
H	-0.59540	-1.08790	-0.64690
H	1.13110	-0.94760	-0.35750
H	-3.02970	-0.96020	5.32540
H	-1.40240	-0.70570	5.98470
H	-2.14580	-2.30890	6.07290
H	-2.16900	0.06410	3.22460
H	-0.44830	0.07390	3.65190
H	-2.02240	-2.85030	2.53950
H	-0.43300	-2.21170	4.25920
H	-1.52140	-0.31150	1.11440
H	-6.58230	-5.03060	0.92620
